# Supplementary material for: Population differences of chromosome 22q11.2 duplication structure predispose differentially to microdeletion and inversion
Source: Nat Commun. 2026 Apr 18;17:3701. doi: 10.1038/s41467-026-71905-y (PMC13103302; doi:10.1038/s41467-026-71905-y)
Supplement: Supplementary file 1 — Supplementary Information [file 41467_2026_71905_MOESM1_ESM.pdf]

Supplementary Information for:

Population differences of chromosome 22q11.2 duplication structure predispose differentially to microdeletion and inversion

Supplementary Notes 1-2, Supplementary Figures 1-58, & Supplementary References

Porubsky et al.

Supplementary Notes

Supplementary Note 1:

Evaluation of 22q11.2 assembly accuracy of human diversity panel. To ensure the quality of assembled haplotypes analyzed in this study, we set to evaluate the assembly accuracy using multiple orthogonal methods and datasets.

1) From the total of 220 haplotypes, we selected only those haplotypes with a region corresponding to the 22q11.2 region (chr22:18000000-23000000, T2T-CHM13 coordinates) assembled in a single continuous contig with no N's within a sequence that would point to scaffolded assembly gaps.
2) We re-aligned PacBio HiFi reads back to each assembly using minimap2 (Methods) in order to evaluate each haplotype assembly for the presence of assembly collapses in highly identical LCR regions (especially in LCRA and D)[1]. The presence of an increased coverage over a defined region along with an increased count of the second most abundant allele is indicative of a collapsed assembly of a piece of DNA present in a given region in more than one copy (Supplementary Fig. 47, Supplementary Data 1).
3) To further evaluate the accuracy of HPRC1 (release 1)[2] assemblies generated using HiFi reads only, we obtained access to HPRC2 (release 2) assemblies completed using both HiFi and ONT reads. For a subset of these samples, we were able to compare assembly accuracy for 35 haplotypes. Of the 35 haplotypes, there are 30 for which the sequence identity between the HPRC1 and HPRC2 assemblies is more than 99.99% (Supplementary Fig. 48). We noticed, however, five haplotypes with a lower sequence accuracy (>99.5% but <99.99%) due to an inversion in a distal part of the LCRD. We predict this is a misassembly within HPRC1 assemblies, so we now report affected haplotypes in Supplementary Data 1. We do still use these five haplotypes in our subsequent analysis as these low-frequency discrepancies have no effect on our prediction of deletion predisposition at 22q11.2 because of the location of the putative misassembly.
4) As another metric of accuracy, we searched for biological replication—i.e., evidence that the same structure had been observed in two independent human genomes. Specifically, we took advantage of available HPRC2 data (Data Availability) to evaluate the accuracy of the higher order structure of LCRA among all singleton haplotypes (n=47) defined among a total of 63 unique LCRA structures. For each singleton structure we searched the 418 HPRC2 structures of LCRA in order to find the best matching one. We did this by encoding each repeat unit by a unique number and comparing them against all HRPC2 structures. We calculated the distance between these numeric encodings of each structure using R package stringdist and its function 'seq_dist' (function parameter: method='osa'). We consider a singleton structure to be validated by HPRC2 if there is a matching HPRC2 structure with less ≤5% divergence. Based on this threshold, we confirm 23 singleton structures are seen at least once in the HPRC2 dataset (Supplementary Fig. 49).
5) Lastly, we compared the organization of LCRA to previous fiber-FISH and optical-mapping-based analyses of the same samples where applicable. To validate the assemblies, the structure of LCRA among the 133 haplotypes has been investigated using the sequence of

the probes previously used to characterize the region by fiber-FISH and optical genome mapping[3].The probe content of each haplotype was inferred using BLAST and selecting matches with at least 87% identity compared to the fiber-FISH probe and with length of at least 50% of the fiber-FISH probe. LCRAs are represented using the repeat units defined by Demaerel et al. (2019)[3]. Most assemblies have a structure that matches what has been previously described. Haplogroups 13* and 14 differ slightly from previously described structures as one probe is only partially included (42% of the probe length is included in the assembly) (Supplementary Fig. 50). A detailed comparison of the structures is summarized in Supplementary Data 2. Similarly, we also validated the assembly-based structure of LCRA and D using fiber-FISH for three family duos (AD009, AD010, and AD013) (Demaerel et al. 2019)[3]. All family duos are in line with the fiber-FISH observed LCRA and D structure (Supplementary Fig. 51). The structural validity of the Coriell trio was confirmed using local reassembly with NOVOLoci[4] (Methods). We, however, observe a putative haplotype switch error downstream from LCRD, which has no effect on the proper mapping of the 22q11.2DS breakpoint (Supplementary Fig. 44).

We note that the chromosome-scale phasing accuracy of all our assembled haplotypes was ensured by using trio-based phasing in HPRC1 assemblies[2] or using Strand-seq phasing information in HGSVC assemblies[5].

Supplementary Note 2:

Gene annotation at 22q11.2. There are a number of protein-coding genes and transcribed pseudogenes overlapping our defined repeat units (25 and 105 kbp). This includes protein-coding genes (*GGTLC3*, *USP18*) and transcribed pseudogenes (*GGT2P*, *GGT3P*, *GGT8P_1*, *POM121L15P*, and *POM121L8P*) overlapping the 105 kbp repeat unit while the 25 kbp repeat unit is occupied by long noncoding RNA (lncRNA) represented by *FAM230* genes. There are also two newly characterized genes (LOC*) at 22q11.2 in the T2T-CHM13 reference (Supplementary Fig. 15).

We performed *de novo* gene annotation and expression analysis on selected human haplotypes of the 22q11.2 region using a database of 1.4 billion Iso-Seq reads[6]. We compared alignments of Iso-Seq reads from *FAM230*, *POM121*, and *GGT* gene family members within the 22q11.2 region to the T2T-CHM13 reference versus the rest of the assemblies (Supplementary Fig. 52). Highly divergent Iso-Seq reads generally correspond to structural variants (SVs), with transcription extending across SV breakpoints. For example, the >10% divergent *GGTLC3* and *GGT3P* reads correspond to *PI4KAP2-GGTLC3* read-through fusion transcripts. We did not observe any novel protein-coding gene models for these families but did observe usage of an isoform of *CLTCL1* corresponding to the *Pan paniscus* gene model XP_063459430.1, which had not been documented in humans and encodes an additional 44 amino acids relative to the canonical human gene model.

FAM230 lncRNA paralogs co-localize with the 25 kbp repeat unit and adjacent palindromic AT-rich repeats (PATRRs) (Supplementary Fig. 15) are associated with structural variation,

including the most common non-Robertsonian translocation, constitutional t(11;22)[7,8]. We observe preferential expression of *FAM230B*, *E*, *F*, *G*, *H*, and *J* in testis and sperm (Supplementary Fig. 53), raising the possibility that paralog transcription may modulate structural variation frequency as has been documented in yeast[9], given the bias towards parental origin for this translocation[10].

With Iso-Seq data, we find embryonic stem cell and fetal striatum expression of *USP41* in LCRB (Supplementary Fig. 54, Supplementary Fig. 55), and despite its annotation as a pseudogene[11], it encodes a 295 amino acid open reading frame. Previous annotations may have been misled by extension into segmental duplication (SD) sequence for *FAM230G*, preventing unique short-read alignment. Our Iso-Seq data indicate that *USP41* has an additional 70 amino acids in three coding exons on its 3' end, relative to previous lncRNA annotations and its ancestral paralog, *USP18* (Supplementary Fig. 56). Recent work provides proteomic support for *USP41* translation and suggests a function distinct from *USP18*[12], which may increase breast cancer cell migration[13]. Investigating the phylogeny of this gene, we predict that *USP18* and *USP41* were likely to be present in African great ape ancestors, although the chimpanzee *USP41* was lost (Supplementary Fig. 55b-c). Constructing the phylogenetic tree, we observed that the expansion of USP genes indeed predates speciation of chimpanzees and gorillas (Supplementary Fig. 57). However, we find that the divergence time between *USP18* and *USP41* is 6.68 [5.5-7.81] MYA, which is earlier than the reported speciation time of gorilla (8.6 [8.3-9.3] MYA[14]). Predictably, the divergence times of the USP gene tree are underestimated, considering relatively short divergence times to gorilla (5.99 MYA) and chimpanzee (4.64 MYA) orthologs, which is approximately one-third shorter than the expectation. Performing a test of positive selection, we find that the rate of nonsynonymous mutation is greater than the rate of synonymous mutation at *USP18*, *USP41*, and the African great ape ancestor branches; however, we also note that the test was statistically insignificant (Supplementary Fig. 58).

Supplementary Figures

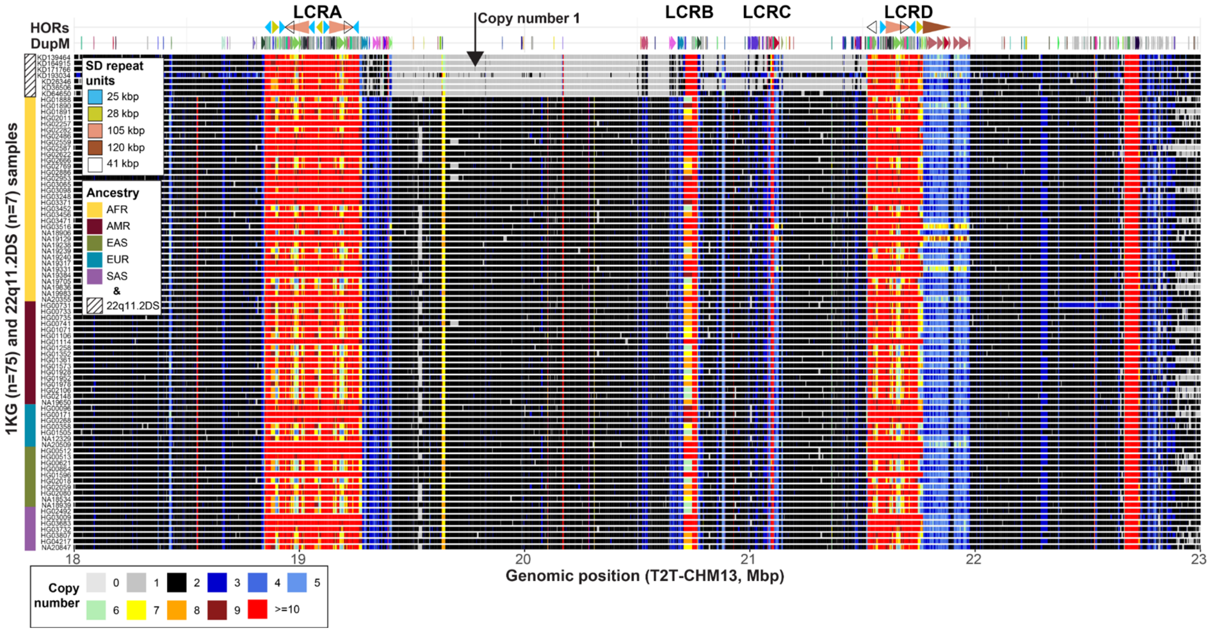

Supplementary Figure 1: Copy number profiles for 75 samples reported in this paper's human diversity panel.

A copy number (CN) heatmap based on high-coverage Illumina read-depth profiles across the 22q11.2 region (T2T-CHM13 coordinates; chr22:18000000-23000000) plotted separately for the 75 samples from the 1KG panel presented in this study. In addition, there are seven samples with 22q11.2DS. Of those, there are five samples with LCRA-D and two with LCRA-B deletions (one sample per row). Color designates the estimated copy number (see legend). The left colored bar highlights samples of the same ancestry (AFR - African, AMR - American, EAS - East Asian, EUR - European, and SAS - Southeast Asian) and 22q11.2DS samples. At the top there are annotations of common SD repeat units (25, 28, 105, 120, and 41 kbp in size) as well as DupMasker annotation. We also mark positions of LCRA and LCRD where the highest copy number (≥10) is reported, which prevents accurate estimates of copies of each common repeat unit.

Analysis note: Read-depth-based copy number estimates were generated using the FastCN[15] software package, which uses known copy number stable regions to correct for Illumina sequencing GC bias and convert read depth to diploid copy number over 1,000 base-pair windows.

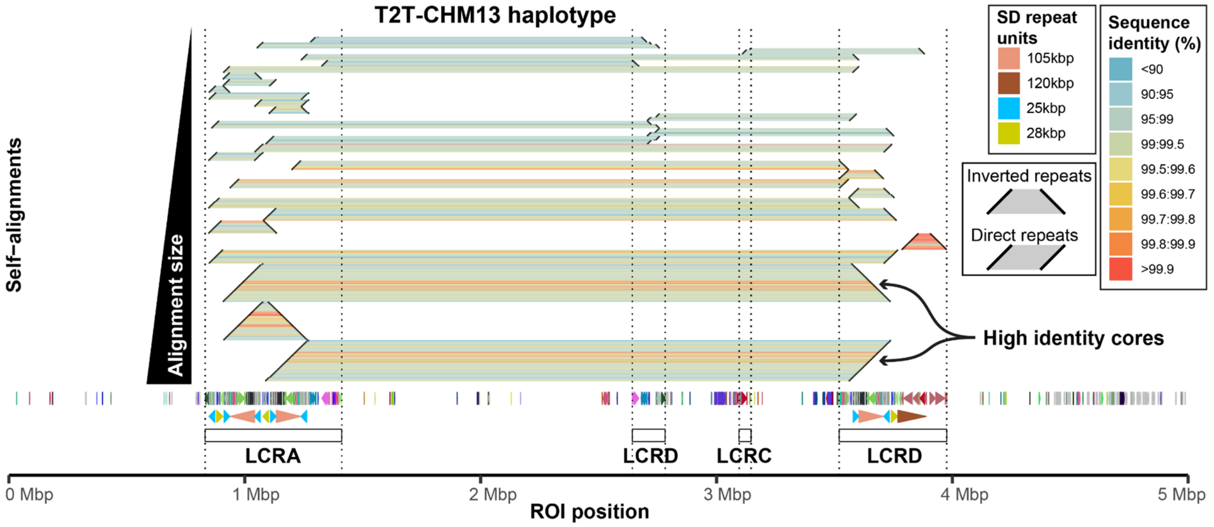

Supplementary Figure 2: T2T-CHM13 self-alignments.
A "horizontal dotplot" visualization showing self-alignments within the T2T-CHM13 reference for the 22q11.2 region. Alignments (diagonally oriented black lines) are connected by horizontal ribbons colored by percentage of matched bases per 2 kbp long bin. Below is a DupMasker annotation of T2T-CHM13 colored by a unique duplicon ID. Further below is the SD repeat unit annotation shown as direction pointing arrowheads. Last, there is annotation of LCR regions A-D. ROI - region of interest (T2T-CHM13, chr22:18-23Mbp).

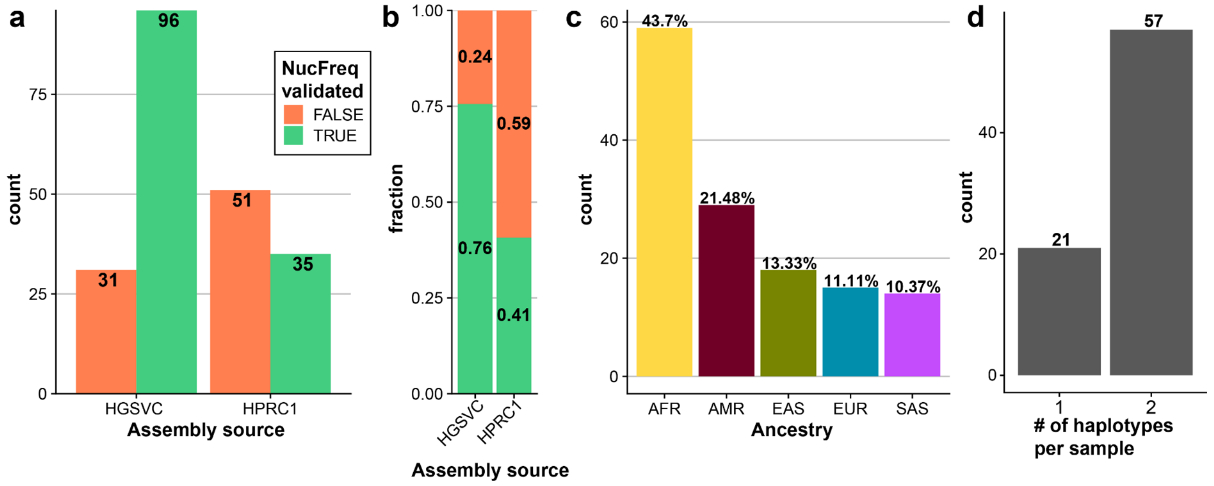

Supplementary Figure 3: Summary of complete assemblies of the 22q11.2 region.
a) Barplot showing the number of complete assemblies (assembled in a single contig) and NucFreq validated assemblies (Valid, TRUE - green; invalid, FALSE - orange) stratified by assembly source (HGSVC, HPRC1 - release 1). **b**) A stacked barplot showing the proportion of all evaluated assemblies being assembled in a single contig and validated by NucFreq[1]. **c**) Barplot showing the counts for all NucFreq validated assemblies stratified by ancestry. **d**) A barplot showing the number of samples with both maternal and paternal haplotypes assembled (n=57) and those with only one haplotype assembled.
Note: Counts in a) and b) do not include four haplotypes from two samples (HG01888 and HG03471) that carry a large-scale inversion over the 22q11.2 loci; thus, the total count reported here is 131. Counts in c) and d) consider the full panel of 135 diverse haplotypes.

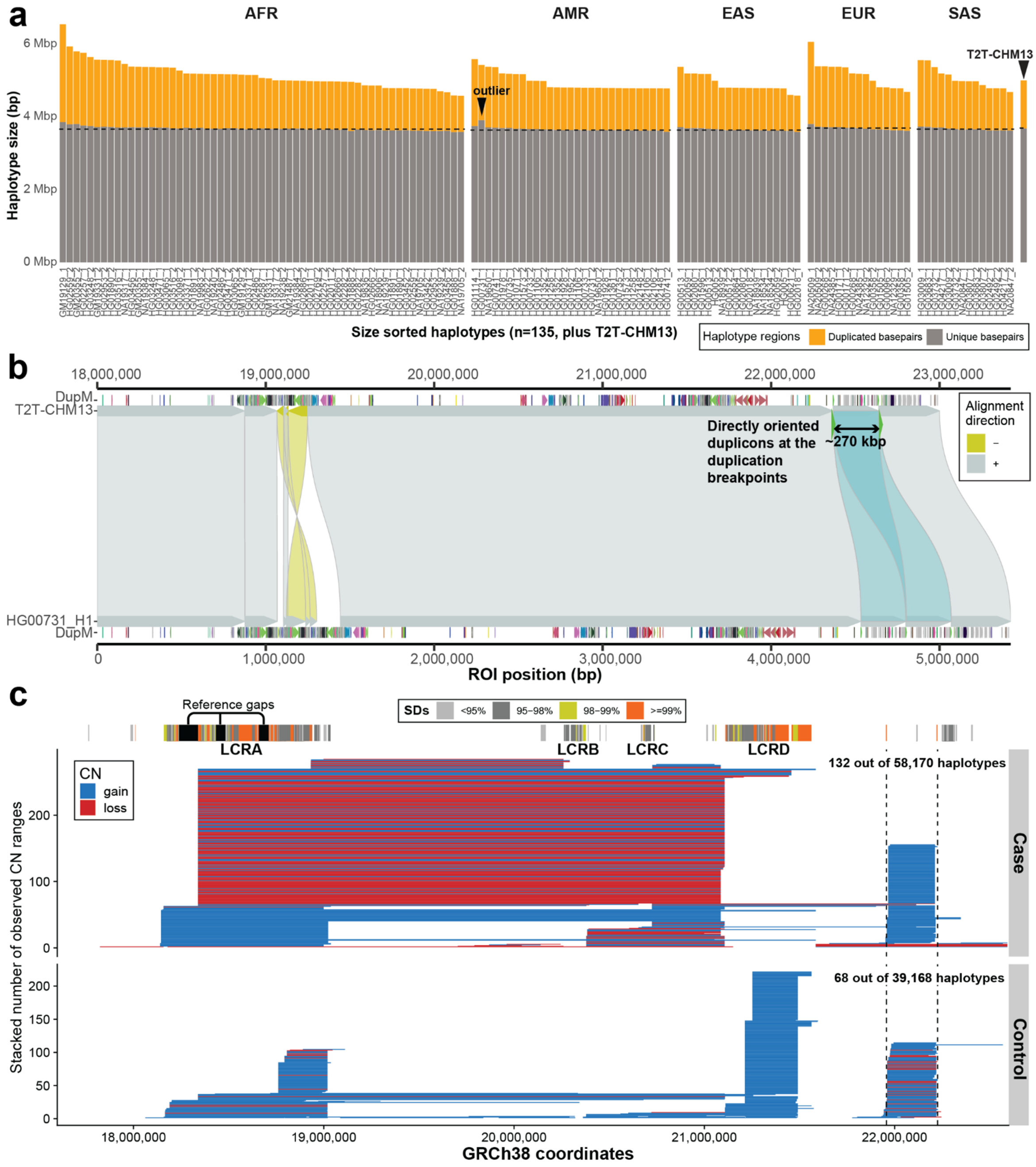

Supplementary Figure 4: Summary of complete assemblies of 22q11.21 region.

a) Barplot showing the proportion of base pairs that are marked as duplicated (orange) and those that are unique (gray) stratified by major ancestries (AFR - African, AMR - American, EAS - East Asian, EUR - European, SAS - Southeast Asian). The median value of unique bases is highlighted by a dashed line per Ancestry. T2T-CHM13 proportions of unique and duplicated bases are shown separately. Each unique haplotype is marked by a sample ID and haplotype-specific (1 or 2) label. A haplotype with an excess of unique bases (HG00731_1) is marked as outlier. **b**) Miropeats-style plot showing alignments (direct - gray, '+' and inverted - yellow, '-') between query (bottom, HG00731-H1) and target (T2T-CHM13, Region of interest - ROI, chr22:18-23Mbp) sequences. There is a duplicon annotation ('DupM' track) specific to query and target sequences shown as directional arrowheads colored by unique duplicon ID. The duplicated sequence (~270 kbp) in HG00731 haplotype 1 is marked by light blue colored alignments. As expected, there are directly oriented duplicons (green arrowheads) at the duplication breakpoints supporting a duplication event via NAHR. **c**) Presentation of previously published data of 29,085 cases with developmental delay along with healthy controls (n=19,584) over the 22q11.2 region[16,17]. Each vertical line represents a defined copy number (CN) region marked either as a loss (red) or gain (blue) of a given region. Only CN

changes of 200 kbp and longer are shown. The duplication defined in panel b) is highlighted by vertical dashed lines. We observe the CN gain among 132 cases and 68 controls.

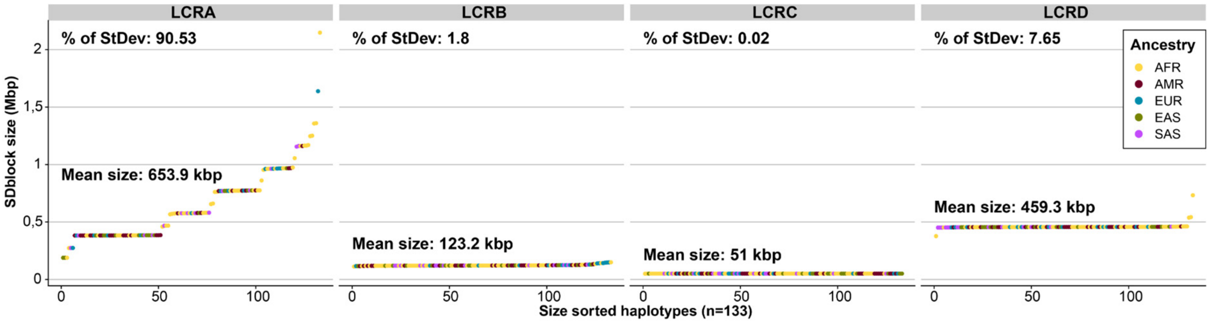

Supplementary Figure 5: Summary of LCRA-D sizes.
Distribution of LCR sizes per haplotype (n=133) colored by Ancestry (AFR - African, AMR - American, EAS - East Asian, EUR - European, SAS - Southeast Asian). For LCRA-D we also report a mean size as well as percentage of standard deviation per LCR (‘% of StDev’).

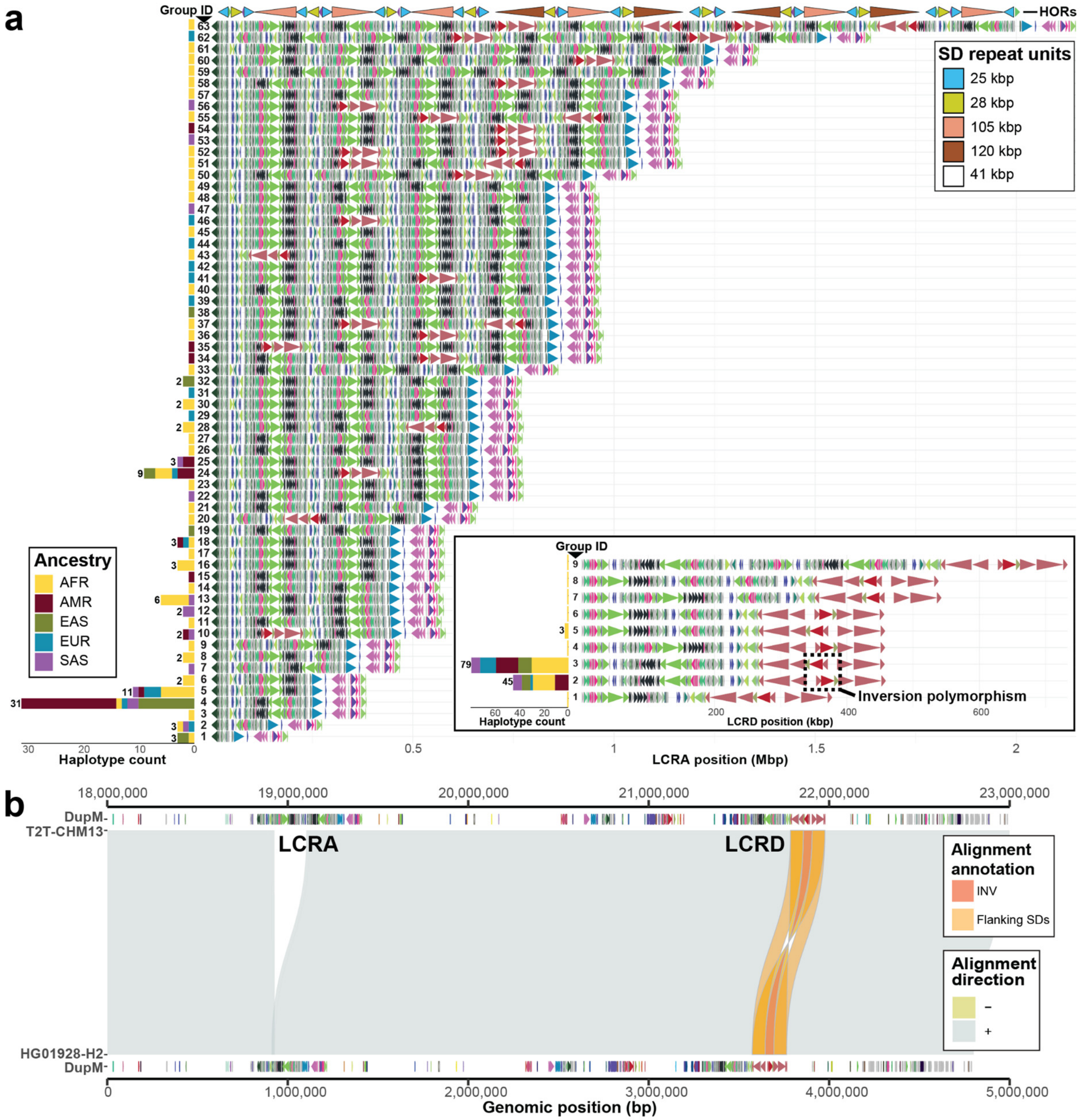

Supplementary Figure 6: Duplicon structure of LCRA and LCRD.

a) Duplicon architecture of 63 distinct LCRA haplogroups (colored arrowheads show size and orientation of individual duplicons as defined by DupMasker[18]). Stacked barplot shows the frequency of each haplogroup among continental population groups. On top there is an annotation of common SD repeat units (25, 28, 105 and 120 kbp in length) along with PATRRs. **Inset**: Shows the duplicon architecture of nine distinct LCRD haplogroups (defined by DupMasker) and their population distribution (left stacked bar plot). A 48.65 kbp inversion distinguishing haplogroups 2 and 3 is highlighted by a dashed rectangle. **b**) Miropeats-style plot showing alignments (direct - gray, '+' and inverted - yellow, '-') between query (bottom, HG01928-H2) and target (T2T-CHM13) sequences. There is a DupMasker annotation ('DupM' track) specific to query and target sequences shown as directional arrowheads colored by a unique duplicon ID. Inverted sequence in HG01928-H2 is highlighted as red colored alignment (48.65 kbp in size) while flanking inverted duplications (~71 kbp in size) are visible as orange-colored alignments between query and target sequences. ROI - region of interest (T2T-CHM13, chr22:18-23Mbp).

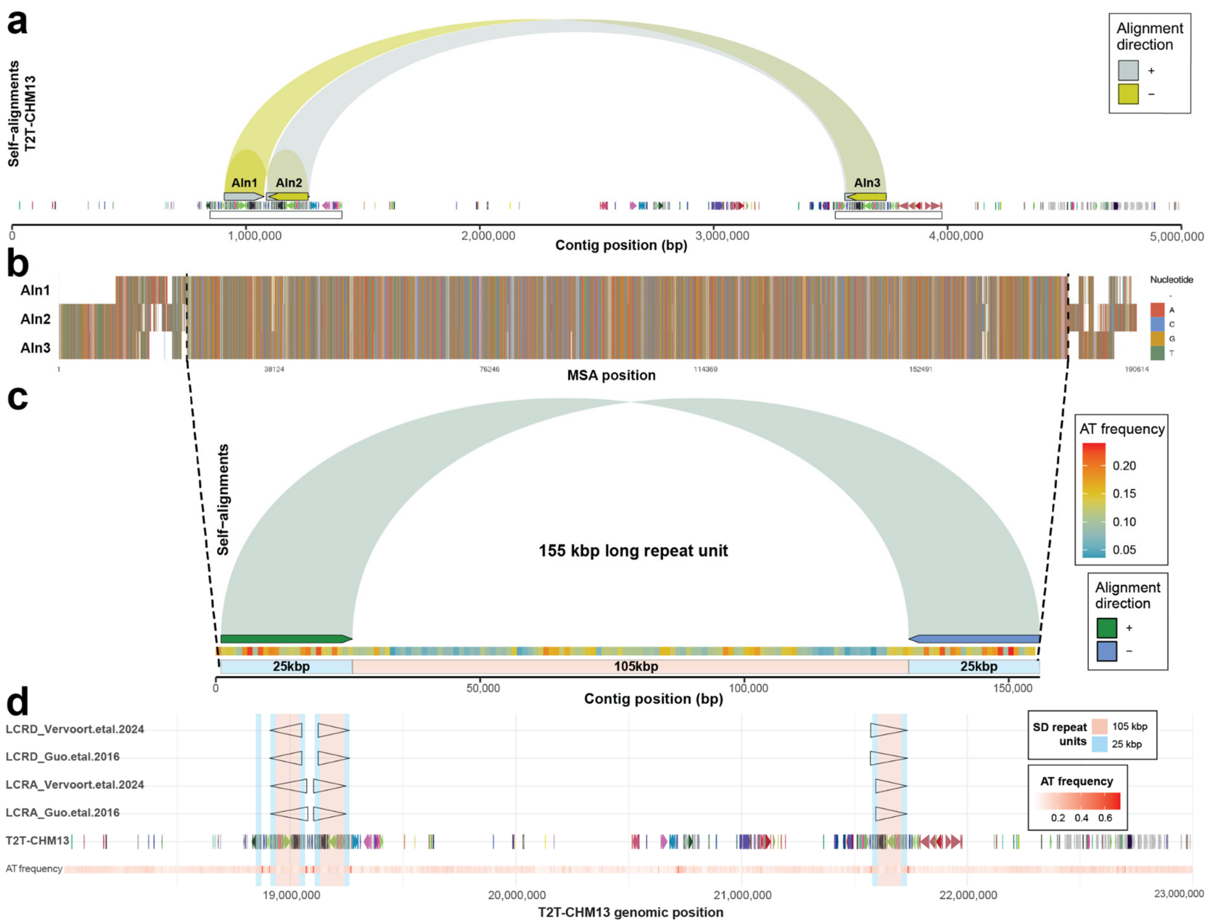

Supplementary Figure 7: Definition of common repeat unit within T2T-CHM13 at 22q11.2.
a) Visualization of long (≥110 kbp) self-alignments within the T2T-CHM13 reference shown as gray and yellow arcs for direct and reverse oriented alignments, respectively. Below is a DupMasker annotation shown as directional arrowheads colored by a unique duplicon ID. Further below are white rectangles delineating LCRA (left) and LCRD (right). **b**) Multiple sequence alignment (MSA) between self-aligning regions (Aln 1-3) from (a). Vertical dashed lines highlight a region that can be continuously aligned between the three sequences including gaps ≤500 bp. **c**) Self-alignment of the consensus sequence (~155 kbp), obtained from the MSA, is shown as an arc connecting flanking 25 kbp long regions in inverted orientation. Below is a binned (bin size 1 kbp) frequency of AT dinucleotides to show there are no extended regions of AT richness. Lastly, we show colored bars highlighting 105 kbp long core and 25 kbp long flanking inverted repeats that compose the 155 kbp long common repeat unit. **d**) Mapping of previously defined common repeat units[8,19] (~160 kbp) to the T2T-CHM13 reference are shown as empty arrowheads. Below is a DupMasker annotation of T2T-CHM13 shown as directional arrowheads colored by a unique duplicon ID. At the bottom there is a binned (bin size 1 kbp) frequency of AT dinucleotides. Mappings of our redefined repeat unit (~155 kbp) are shown as vertical rectangles colored by 105 kbp long core (red) and 25 kbp long inverted repeats (blue).
Note: See Methods section ‘Definition of shared repeat segments between LCRA and D’ for more details.

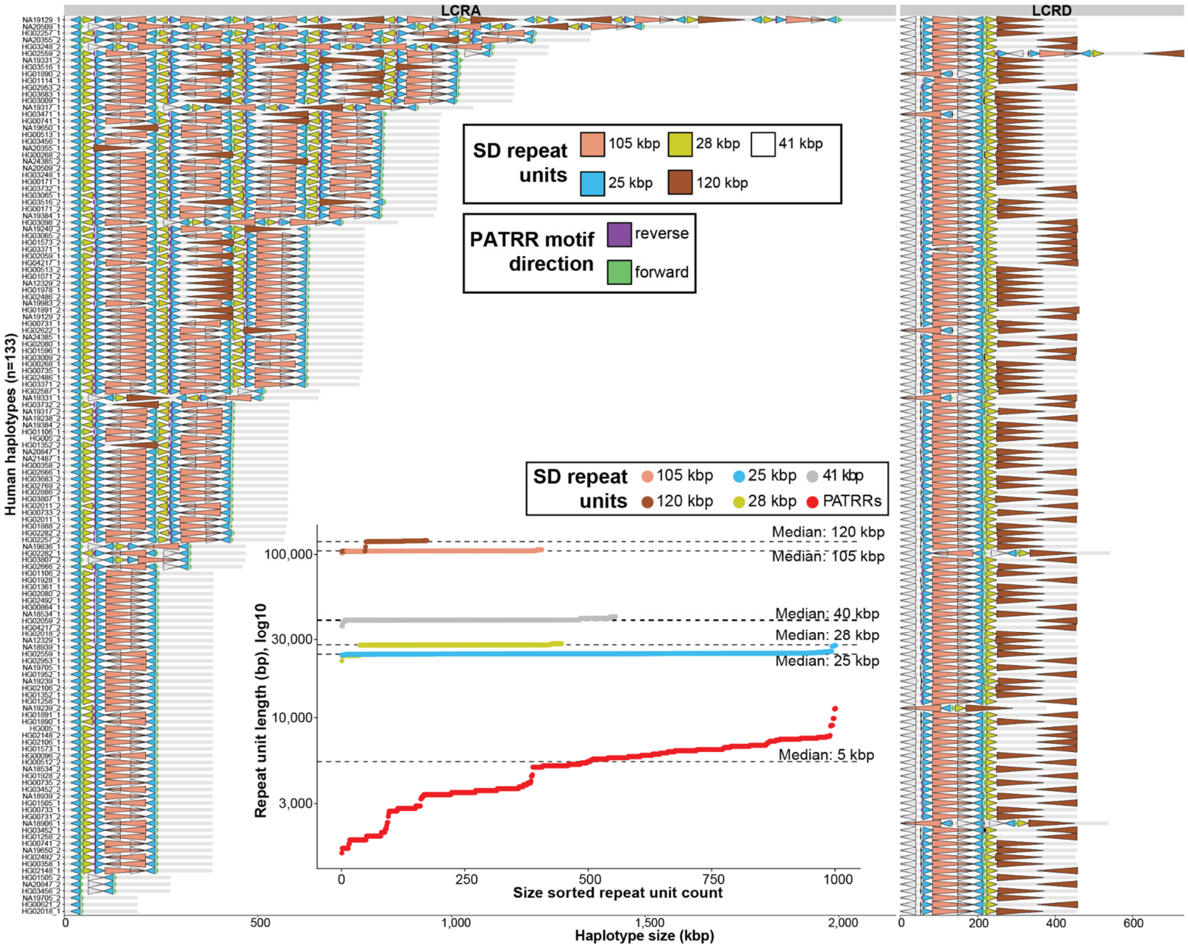

Supplementary Figure 8: Higher order structure of LCRA and D among human haplotypes (n=133). Higher order units in this plot were defined based on self-aligning regions between LCRA and D at base-pair resolution using the T2T-CHM13 haplotype as a reference to define sequence of each unit (25, 28, 41, 105 and 120 kbp in length). Here, each repeat unit in each human haplotype (in rows) is shown as an arrowhead to reflect the directionality of each repeat in LCRA and D (in columns). **Inset**: Median size across all paralogous copies of each repeat unit in LCRA and D.

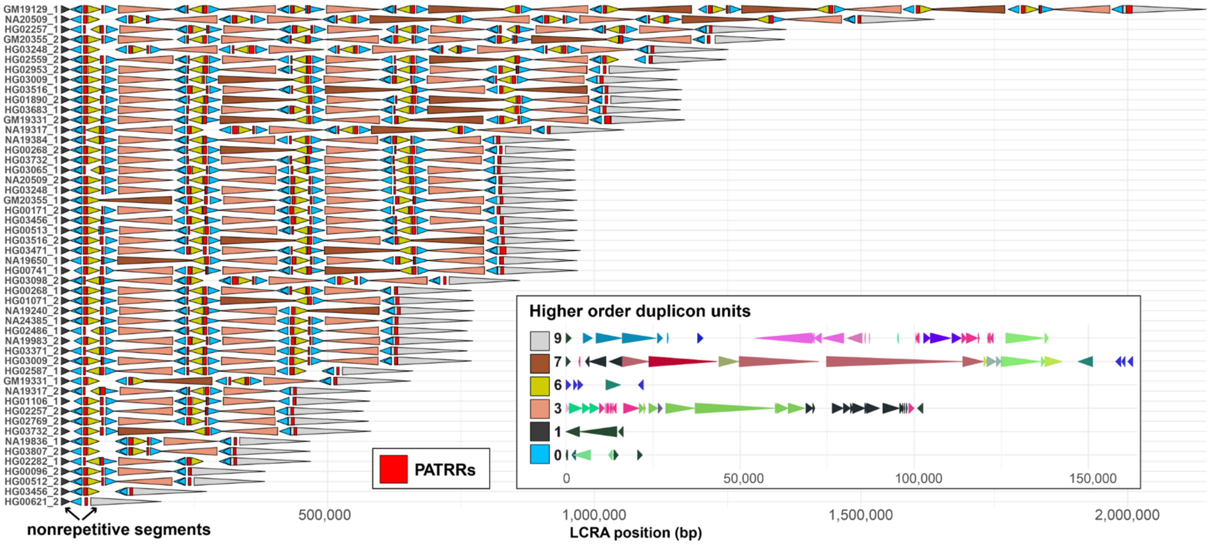

Supplementary Figure 9: Higher order duplicon structure of each representative haplotype of LCRA (n=50). Higher order structure was defined based on the repeated duplicon units as defined by DupMasker (**Methods**). We defined a total of six nonredundant units of which four (unit ID: 0, 3, 6, and 7) correspond to common SD repeat units (25 kbp, 105 kbp, 28 kbp, and 120 kbp, respectively) between LCRA and D as shown in **Supplementary Figure 8**. The remaining two units (shades of gray, nonrepetitive segments) are the flanking units of each LCRA haplotype and are not present in LCRD. We also highlight positions of palindromic AT-rich repeats (PATRRs) as red rectangles in each haplotype. **Inset**: Representation of six nonredundant duplicon units.

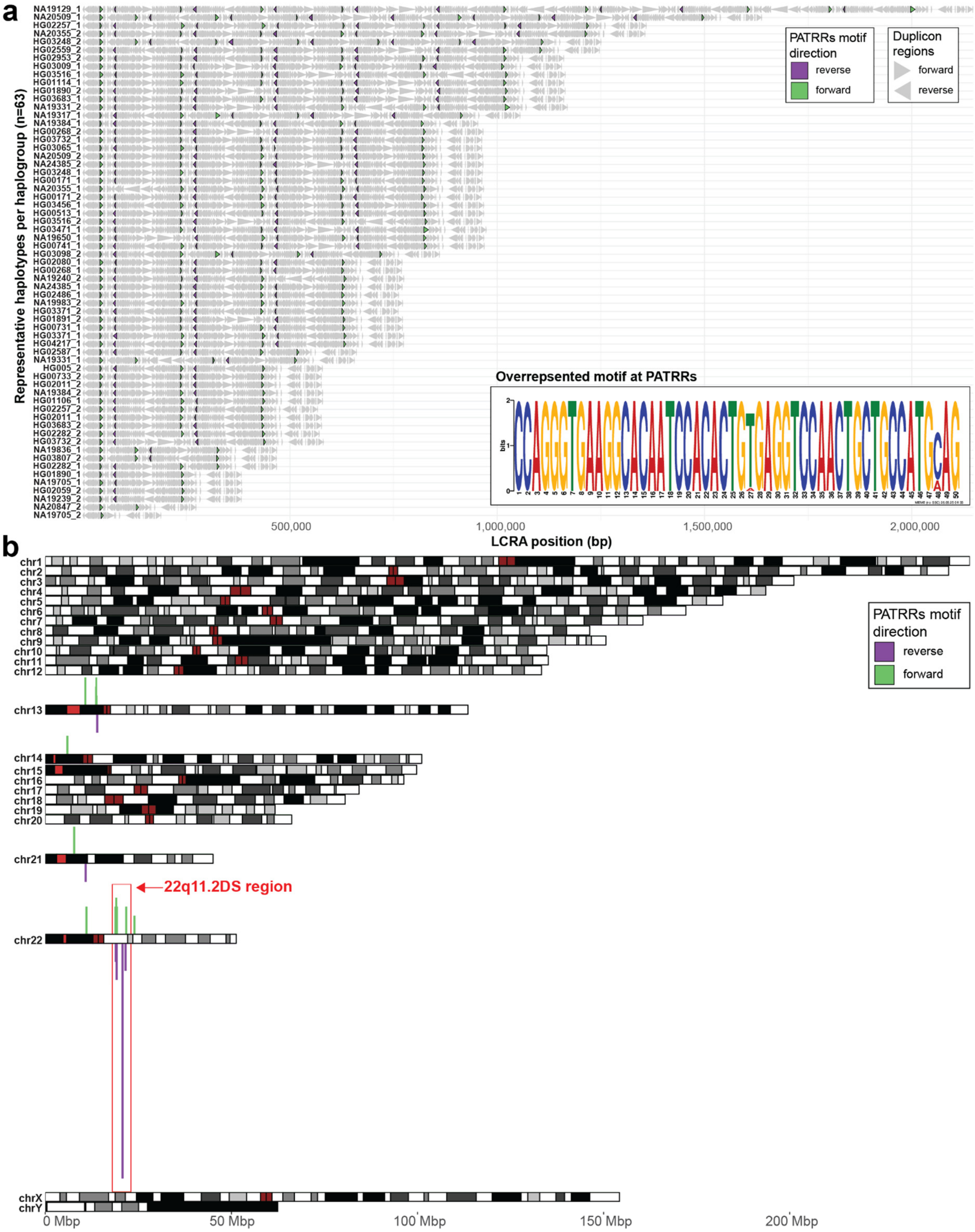

Supplementary Figure 10: Position of PATRRs in each representative haplotype (n=63).

a) DupMasker structure shown as a set of directional arrowheads all colored in gray. Pockets of PATRRs are highlighted as arrowheads colored by the motif orientation (see inset) (forward - purple, reverse - green) within each haplotype as defined using a FASTA sequence for each haplotype (**Methods**). **Inset**: Sequence logo of the overrepresented motif defined among PATRRs. **b**) Frequency of PATRR motif sequence (FIMO score ≥ 50) counted in 200 kbp long bins along each chromosome from the T2T-CHM13 reference. Direction of each matched motif is recorded and counted separately as forward (purple) and reverse (green) oriented motifs.

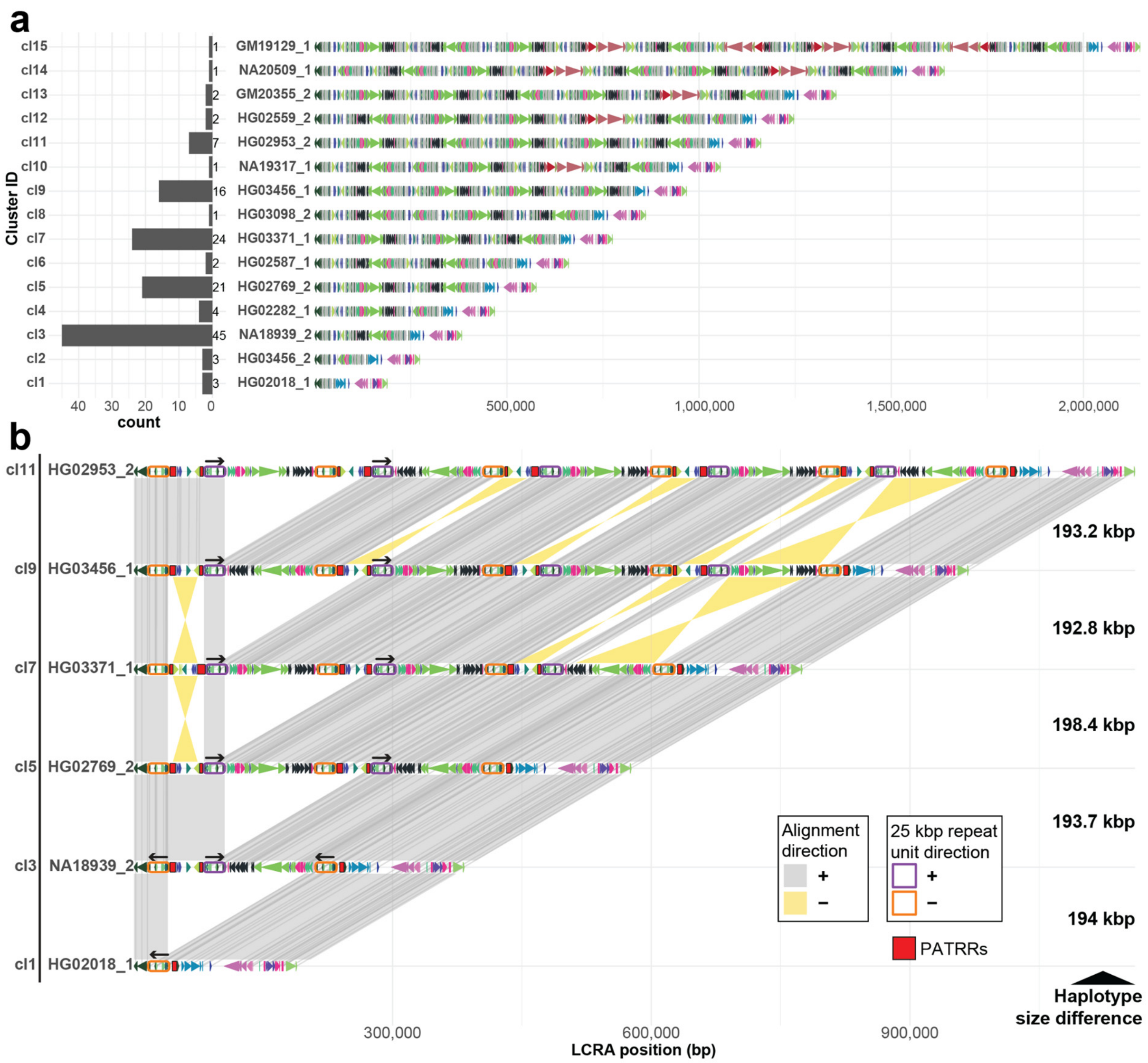

Supplementary Figure 11: Gradual size expansion of SD block A.

a) Left barplot shows a count summary of all 133 haplotypes clustered by their size as shown in **Figure 2b**. On the right we show DupMasker annotation of manually selected representative haplotypes for each size group from (a). They are shown as directional arrowheads colored by a unique duplicon ID. **b**) Selected haplotypes from (a) that belong to size groups with the highest haplotype count. Their DupMasker structure is reported along with putative alignments between haplotypes. Position and direction of the 25 kbp long repeat unit is shown for direct (purple) and reverse (orange) oriented repeats. PATRR positions are shown as red rectangles. The number in between in each subsequent pair of haplotypes reports their size difference.

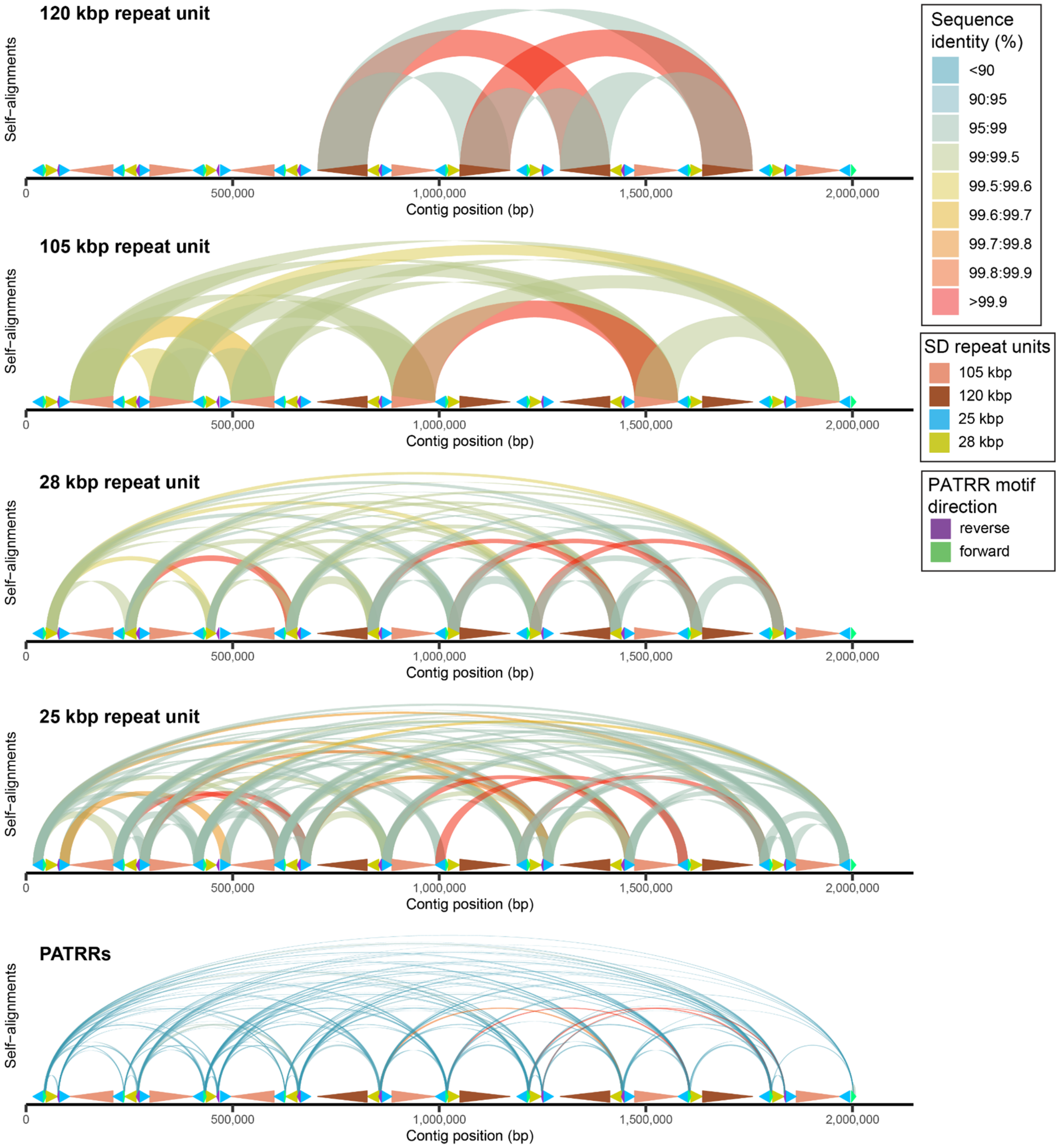

Supplementary Figure 12: Levels of palindrome complexity at LCRA.
Visualization of alignments between defined repeat units (25, 28, 105 and 120 kbp in size) and PATRRs (~5 kbp in size) for the longest LCRA haplotype in sample NA19129-H1. Each alignment is colored by the percentage of matched bases. There is colored annotation of all repeat units and PATRRs (see legend) with arrowhead orientation reflecting the orientation of each repeat unit and PATRRs within the haplotype.

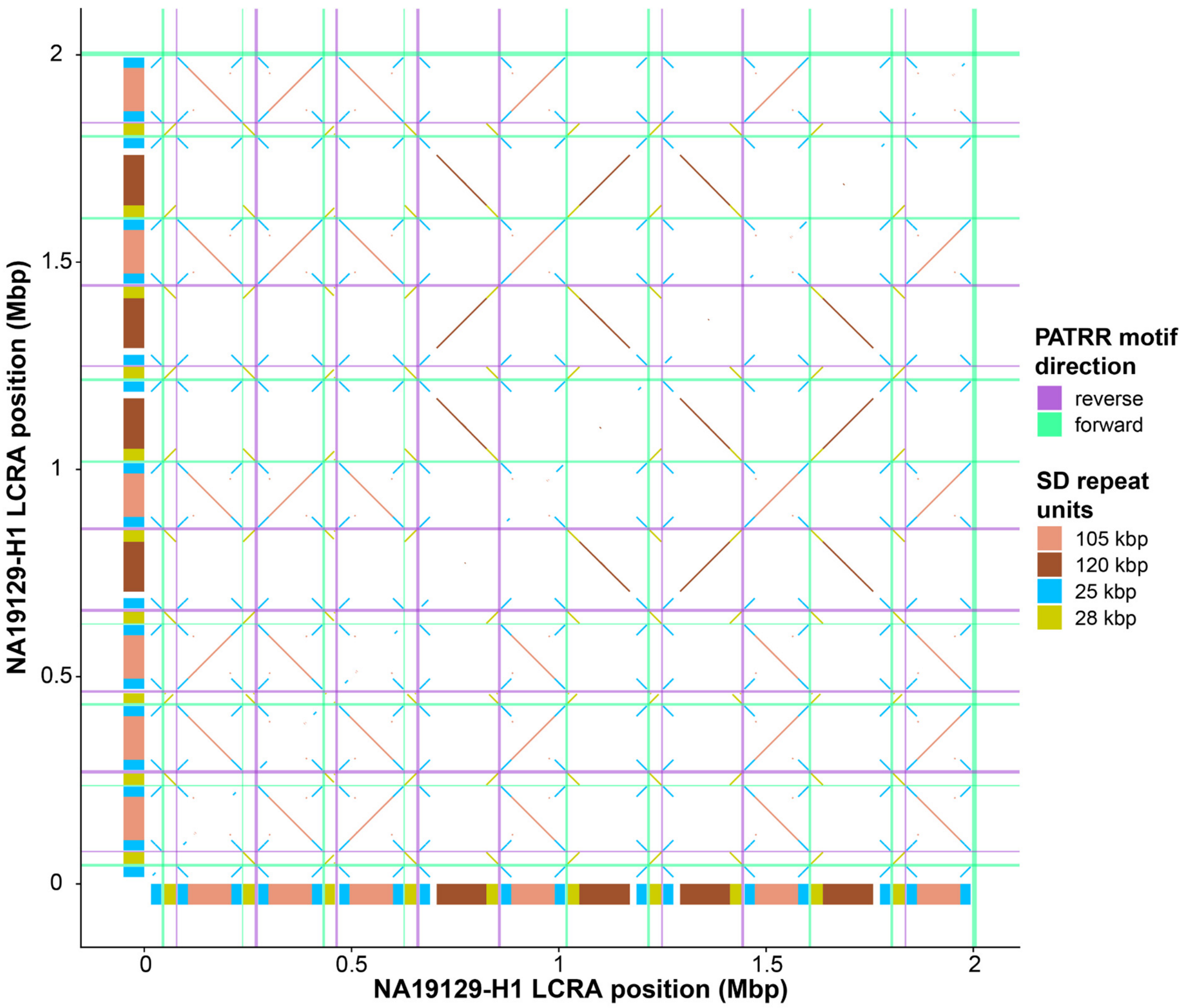

Supplementary Figure 13: Levels of palindrome complexity at LCRA.
Visualization of alignments between defined repeat units (25, 28, 105 and 120 kbp in size) and PATRRs (~5 kbp in size) for the longest LCRA haplotype in sample NA19129-H1 as an alignment dotplot. Each alignment (diagonal lines) is colored by the repeat unit identity. PATRR positions are shown as horizontal and vertical rectangles colored by PATRR motif direction (see legend).

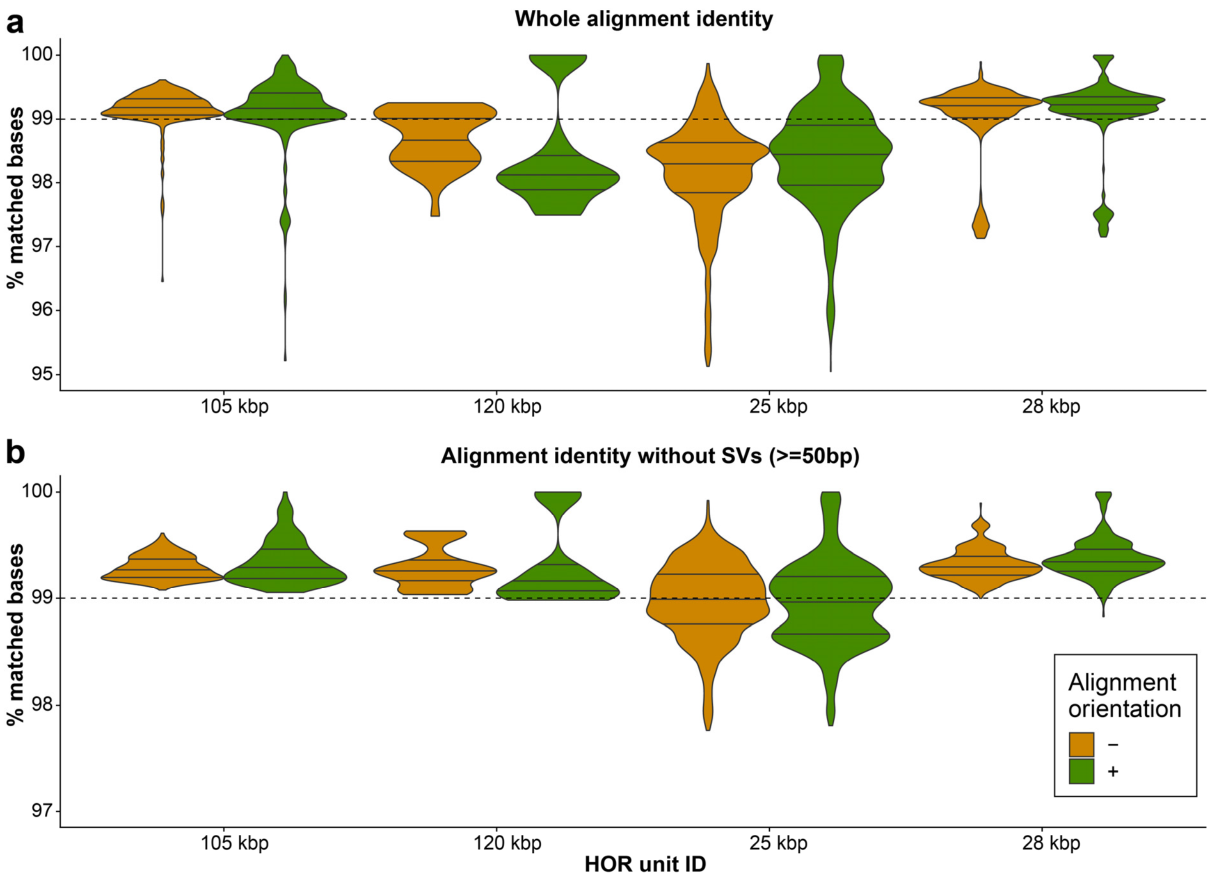

Supplementary Figure 14: Sequence identity of higher-order repeat (HOR) units.
Distribution of sequence identity (measured as a percentage of matched bases in an alignment) for all alignments between paralogous copies of each HOR unit (25, 28, 105, and 120 kbp) within each human haplotype (n=133). Panels **a**) and **b**) show the alignment identity calculated before and after removal of SVs from identity calculation, respectively. Alignment identity distributions are stratified by the alignment orientation (orange - reverse, '-' and green - direct, '+'). Three horizontal lines within each mark the distribution quantiles (0.25, 0.5, and 0.75).

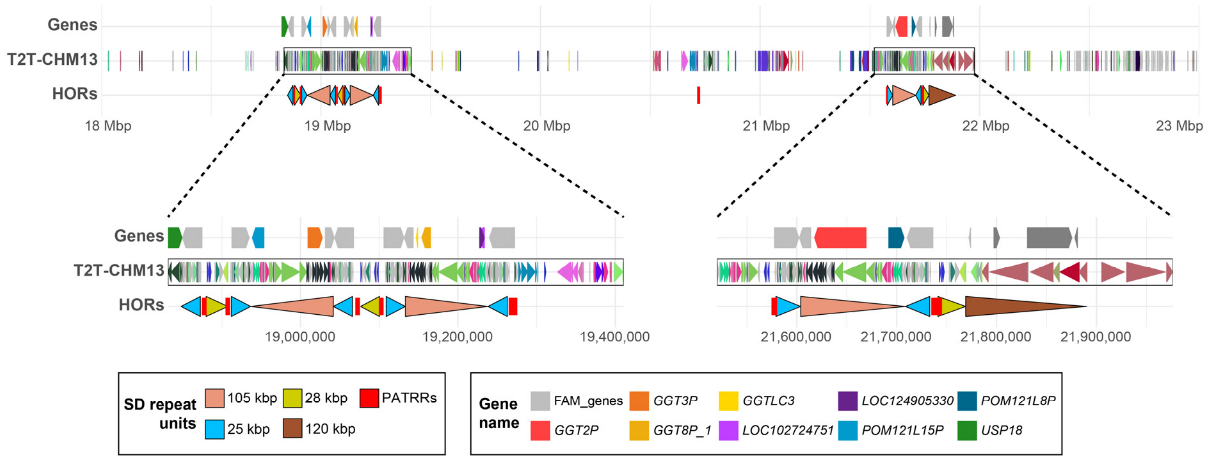

Supplementary Figure 15: Genes overlapping defined repeat units in T2T-CHM13 coordinates.
From top to bottom row we show gene boundaries colored by gene name. Then there is a DupMasker annotation shown as a set of directional arrowheads colored by a unique duplicon ID. Last, there is an SD repeat unit (or HOR) annotation colored by unit ID (25, 28, 105, and 120 kbp) along with PATRR positions colored as red rectangles.

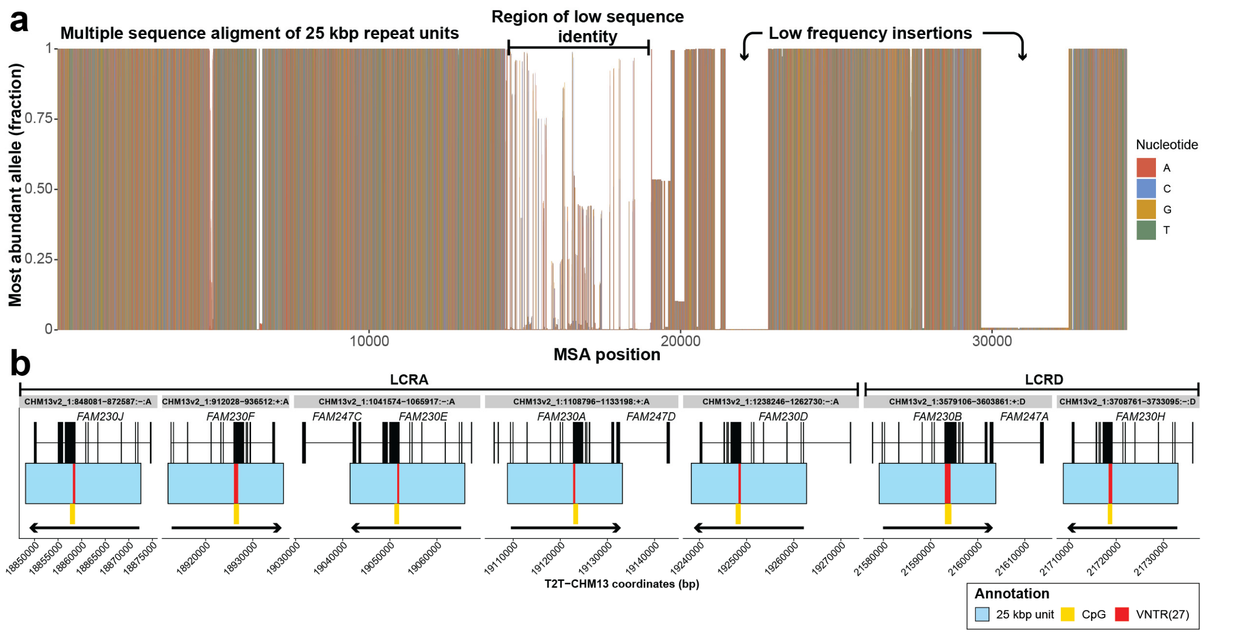

Supplementary Figure 16: Sequence identity and structure of 25 kbp long repeat unit.
a) MSA using all copies (n=1021) of 25 kbp long repeat units among 134 human and 4 nonhuman primate haplotypes (chimpanzee and bonobo). The height of each bar represents the fraction of the most abundant nucleotide (allele) at any given position in the MSA. There are low-frequency insertions in the distal part of the repeat unit but those have only a marginal effect on overall sequence identity among all alignments between copies of this repeat unit. In the middle we observe an extended region of low sequence identity likely caused by the difficulty to align this region. **b**) Visualization of all copies of 25 kbp repeat units (blue rectangles) present in the T2T-CHM13 reference with respect to positions of *FAM230* genes (black rectangles - exons connected by black line). The VNTR position is marked within each repeat unit by a red rectangle followed by the annotation of the known CpG islands in T2T-CHM13 (yellow rectangles). The orientation of each 25 kbp repeat unit is shown by black arrows.

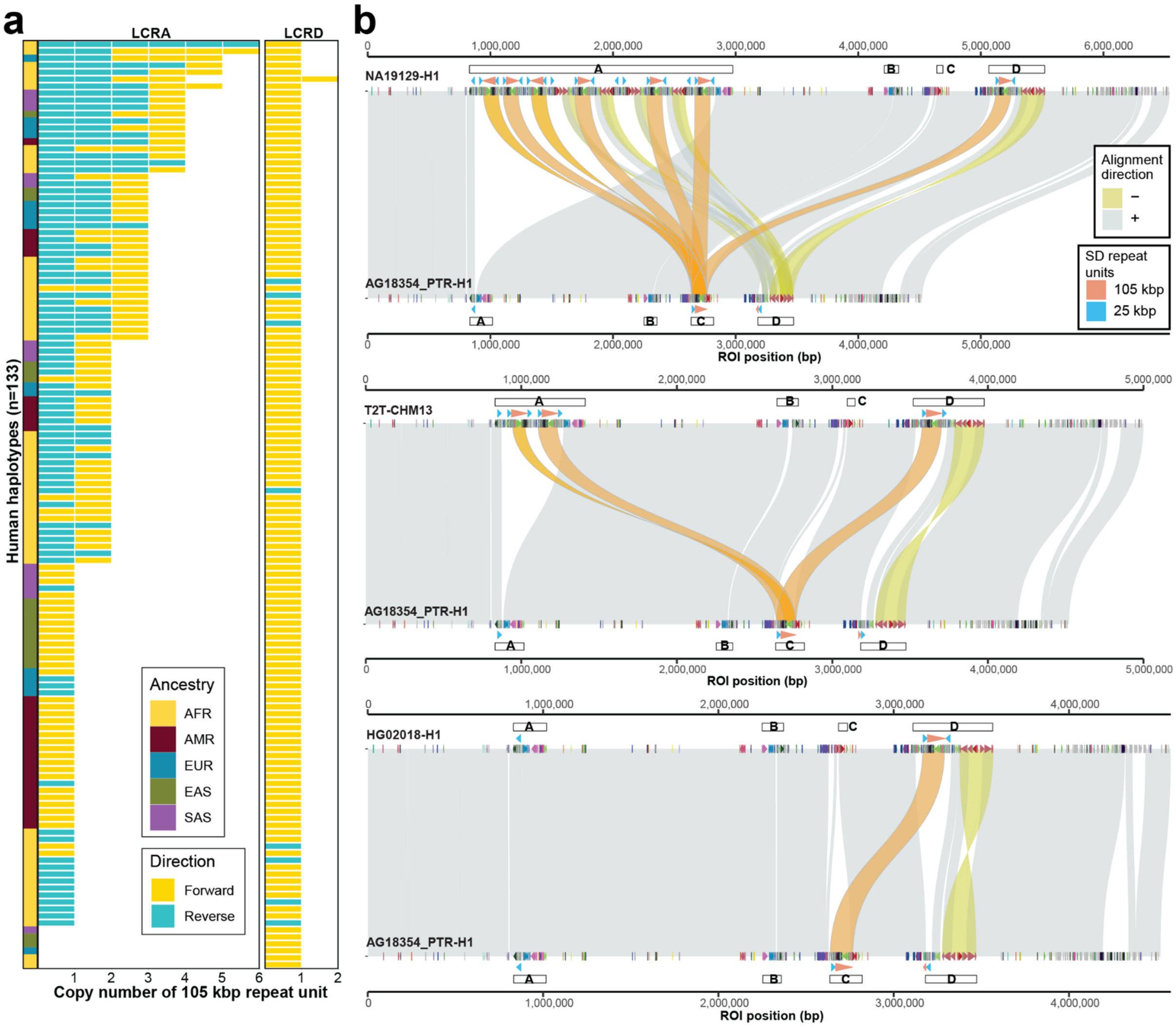

Supplementary Figure 17: Copy number of 105 kbp repeat unit in humans.

a) Counts of main repeat unit (105 kbp) in each human haplotype (n=133) stratified by SD block identity (A or D) and alignment orientation (forward '+' - yellow; reverse '-' - blue). The ancestry of each haplotype is defined in the leftmost panel. **b**) Miropeats-style plot showing alignments (direct - gray, '+' and inverted - yellow, '-') between query (bottom) and target (top) sequences. The top plot shows comparison of chimpanzee haplotype 1 (AG18354_PTR-H1, query) to the NA19129 haplotype 1 (target), which is a human haplotype with the highest number of 105 kbp repeat units. The middle plot shows the copy number of the 105 kbp repeat unit in the T2T-CHM13 reference while the bottom shows the haplotype with the least number of copies of the 105 kbp unit (HG02018-H1). Alignments of the 105 kbp repeat unit are highlighted by light orange color. There is a duplicon annotation track specific to query and target sequences shown as directional arrowheads colored by unique duplicon ID. Positions of LCRA-to-D are depicted by white rectangles positioned above the target and below the query sequences, respectively. ROI - region of interest (T2T-CHM13, chr22:18-23Mbp).

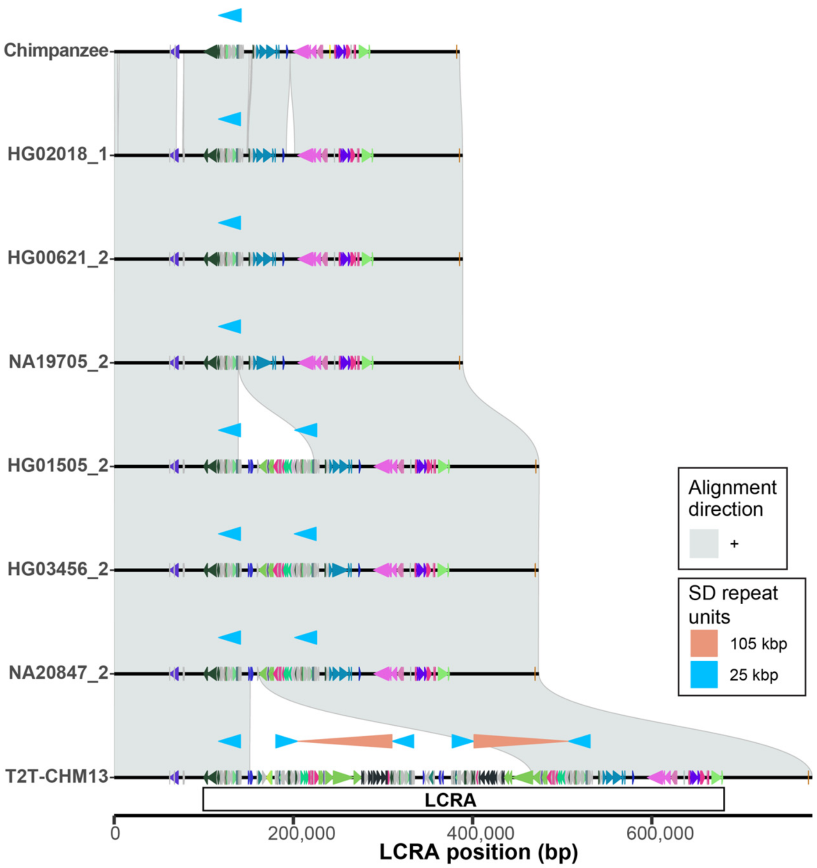

Supplementary Figure 18: Ancestral structure of LCRA in humans.
Progressive alignments ('+' - directly oriented alignments) between ancestral LCRA structure (chimpanzee) and multiple human haplotypes most similar to the ancestral haplotype, including a human haplotype that carries a single copy of the 105 kbp repeat unit as well as the T2T-CHM13 reference that carry two copies of the 105 kbp repeat unit in LCRA. On top of each haplotype is a DupMasker annotation shown as directional arrowheads colored by a unique duplicon ID. Above each haplotype we show an annotation of various repeat units, namely 25, 28, and 105 kbp repeat units, including PATRR positions.

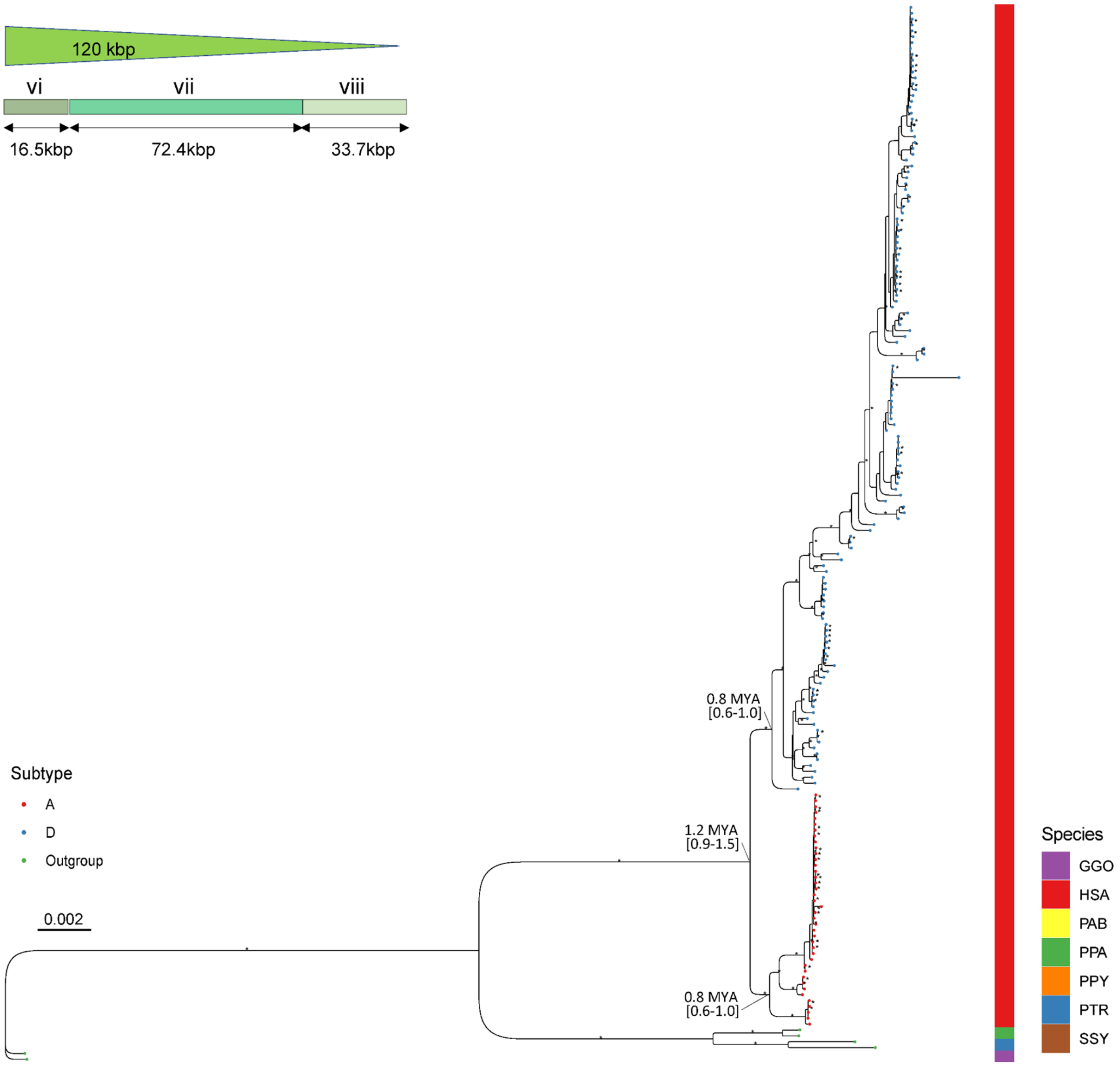

Supplementary Figure 19: Phylogenetic tree of duplicon (vi-viii) of length 120 kbp (n=180).
The tree was outgrouped with gorilla orthologs. The human copies located in LCRA and D are indicated as red and blue, respectively. The corresponding species is indicated on the right annotation track; chimpanzee (PTR), bonobo (PPA), human (HSA), and gorilla (GGO). Notable divergence times among human copies are indicated as million years ago (MYA) with the 95% confidence interval. The bootstrap value of >95 is indicated as an asterisk.

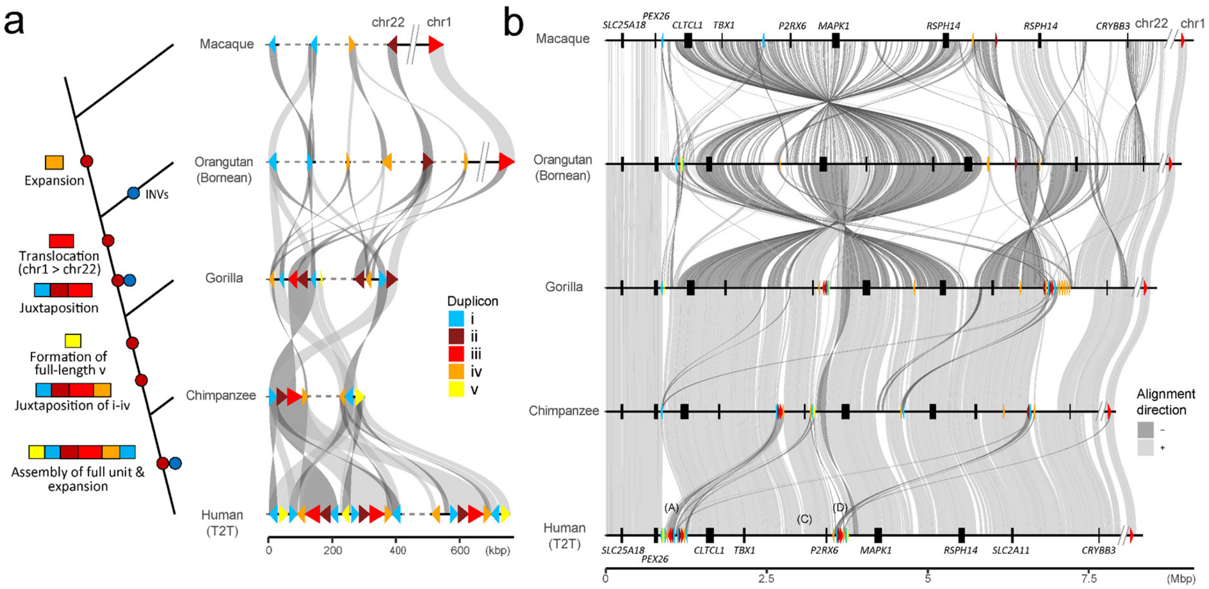

Supplementary Figure 20: Evolutionary model for formation of complex duplication.
a) Schematics of the evolution model, illustrating the emergence of complex duplication observed in human, through multiple steps: 1) expansion of duplicon iv, 2) translocation of duplicon iii from chr1 to chr22, 3) juxtaposition/transposition of complex duplication i-ii and i-iii, 4) formation of full-length duplicon v, 5) juxtaposition of complex duplication i-iv, and 6) formation of full-length human duplication and further expansion of the complex duplication. **b**) Syntenic view of chromosome 22 18-26 Mbp (T2T-CHM13 reference coordinates) region as well as original position of duplicon iii located in chr1. Syntenic genes are indicated along with the duplicons in their respective colors.

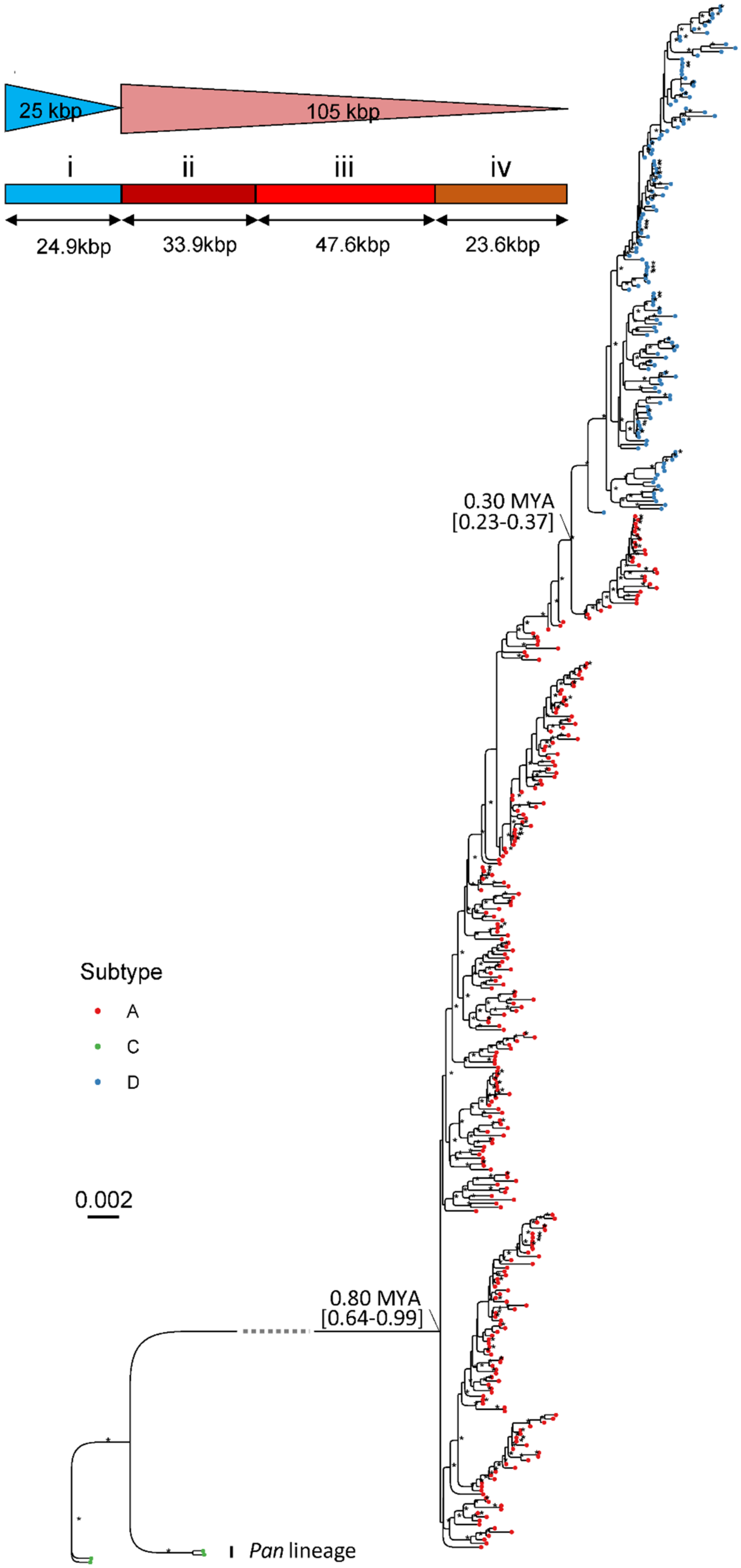

Supplementary Figure 21: Phylogenetic tree of duplicons (i-iv) of length 105 kbp (n=413).
The tree was outgrouped with chimpanzee and bonobo (*Pan* lineage) orthologs. The copies located in LCRA, C, and

D are indicated as red, green and blue, respectively. Notable divergence times among human copies are indicated as million years ago (MYA) with the 95% confidence interval. The bootstrap value of >95 is indicated as an asterisk.

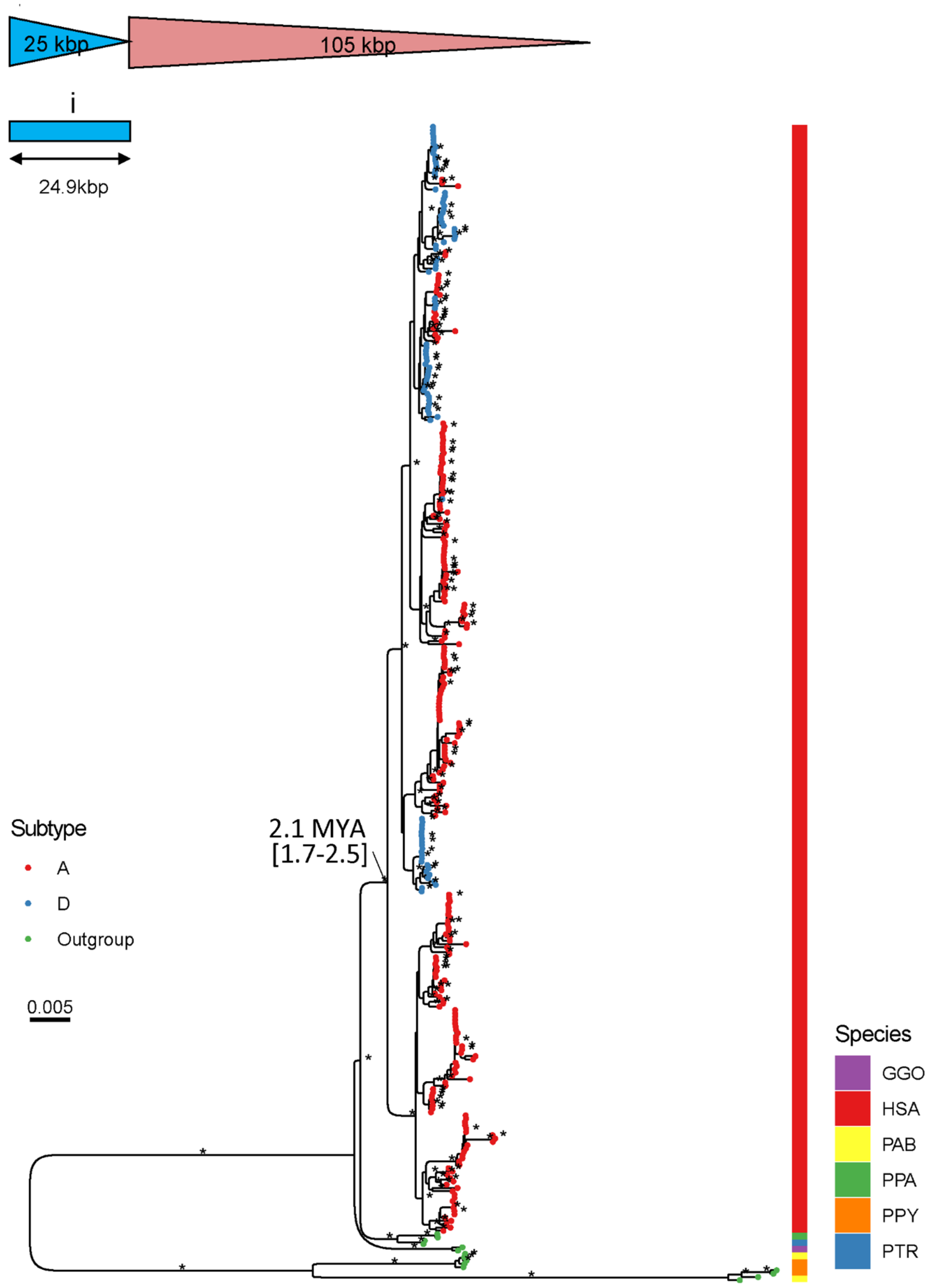

Supplementary Figure 22: Phylogenetic tree of duplicon (i) of length 25 kbp (n=1024).
The tree was outgrouped with macaque and orangutan orthologs. The human copies located in LCRA and D are indicated as red and blue, respectively. The corresponding species is indicated on the right annotation track; chimpanzee (PTR), bonobo (PPA) and human (HSA), gorilla (GGO), Sumatran orangutan (PAB), Bornean orangutan (PPY). Notable divergence times among human copies are indicated as million years ago (MYA) with the 95% confidence interval. The bootstrap value of >95 is indicated as an asterisk.

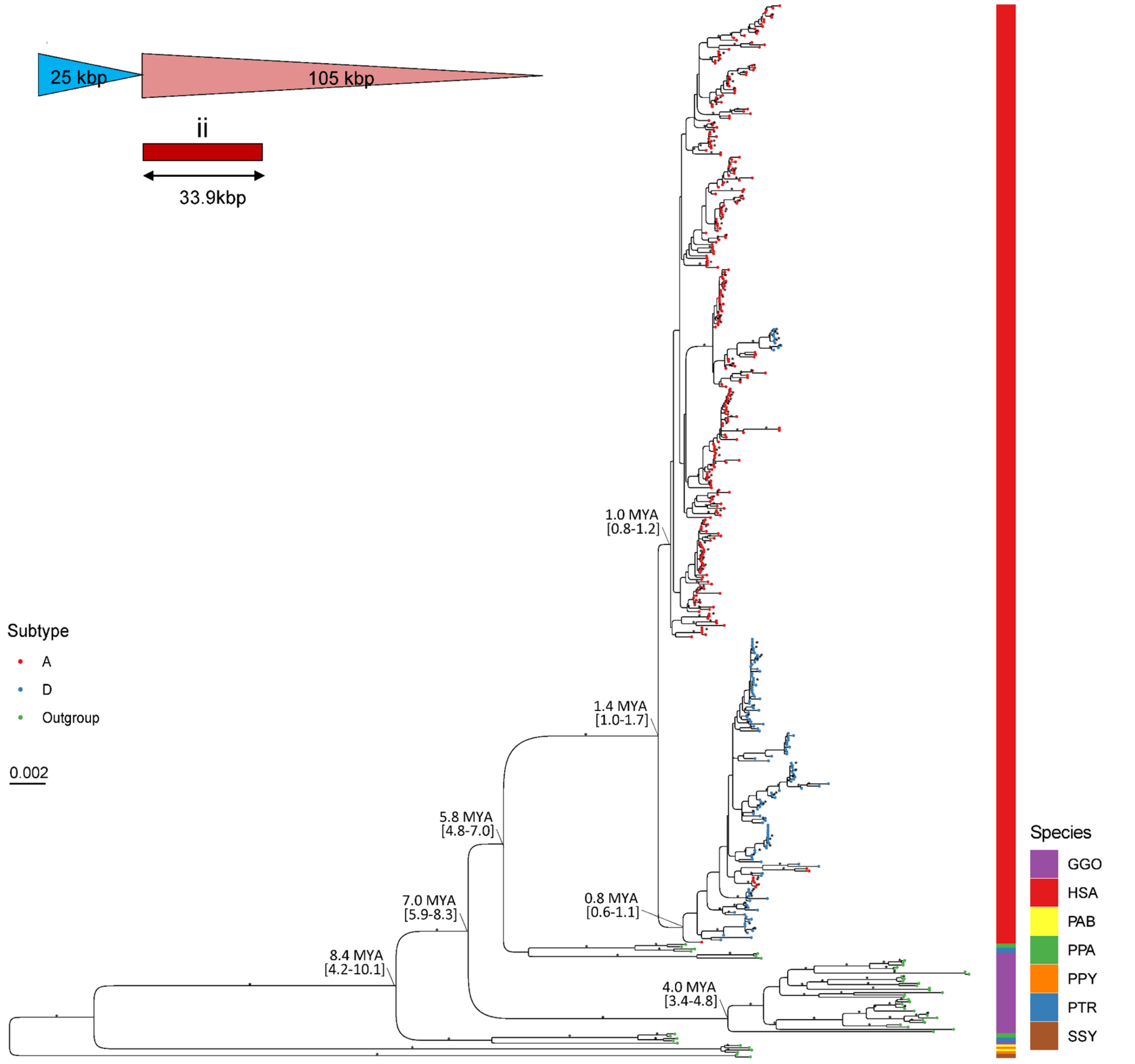

Supplementary Figure 23: Phylogenetic tree of duplicon (ii) of length 33.9 kbp (n=460).
The tree was outgrouped with single-copy macaque ortholog (absent in the tree). The human copies located in LCRA and D are indicated as red and blue, respectively. The corresponding species is indicated on the right annotation track; chimpanzee (PTR), bonobo (PPA), human (HSA), gorilla (GGO), Sumatran orangutan (PAB), Bornean orangutan (PPY) and siamang (SSY). Notable divergence times among human copies are indicated as million years ago (MYA) with the 95% confidence interval. The bootstrap value of >95 is indicated as an asterisk.

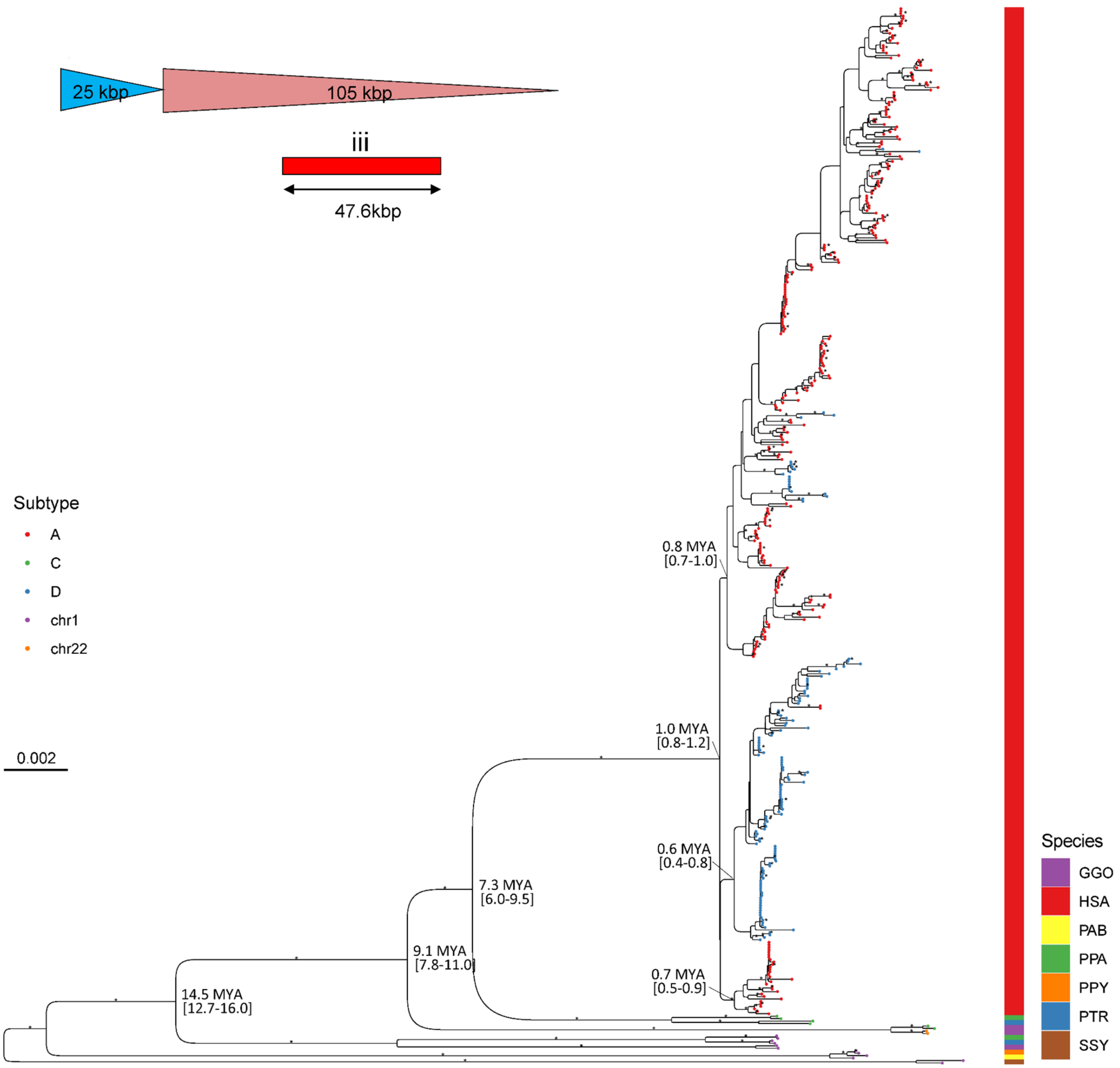

Supplementary Figure 24: Phylogenetic tree of duplicon (iii) of length 47.6 kbp (n=430).
The tree was outgrouped with single-copy macaque ortholog (absent in the tree). The copies located in LCRA, C and D are indicated as red, green and blue, respectively. Additionally, the chromosomal origins (chr1 or 22) of the orthologs are indicated as violet and orange, respectively. The corresponding species is indicated on the right annotation track; chimpanzee (PTR), bonobo (PPA), human (HSA), gorilla (GGO), Sumatran orangutan (PAB), Bornean orangutan (PPY) and siamang (SSY). Notable divergence times are indicated as million years ago (MYA) with the 95% confidence interval. The bootstrap value of >95 is indicated as an asterisk.

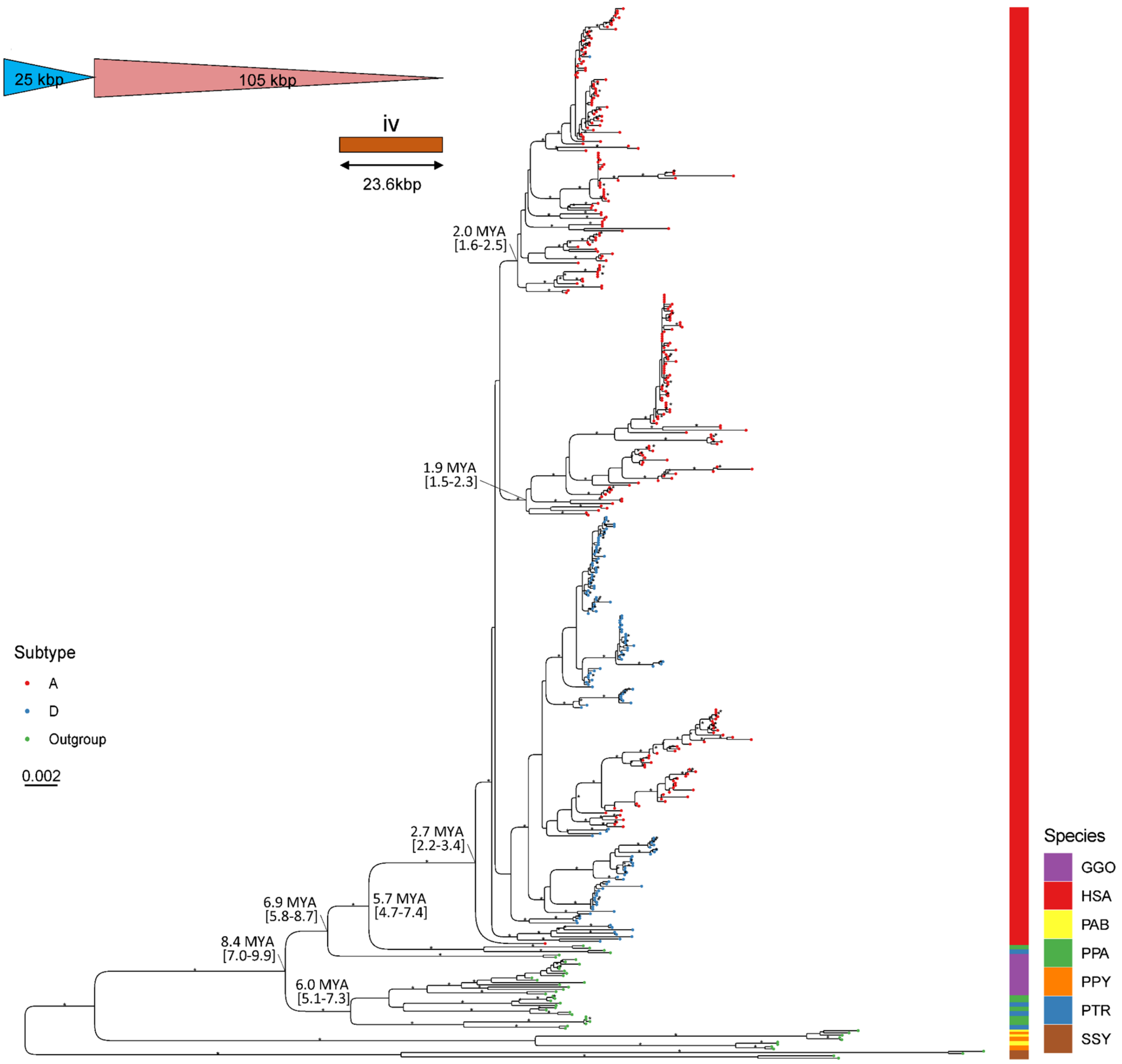

Supplementary Figure 25: Phylogenetic tree of duplicon (iv) of length 23.6 kbp (n=460).
The tree was outgrouped with single-copy macaque ortholog (absent in the tree). The human copies located in LCRA and D are indicated as red and blue, respectively. The corresponding species is indicated on the right annotation track; chimpanzee (PTR), bonobo (PPA), human (HSA), gorilla (GGO), Sumatran orangutan (PAB), Bornean orangutan (PPY) and siamang (SSY). Notable divergence times are indicated as million years ago (MYA) with the 95% confidence interval. The bootstrap value of >95 is indicated as an asterisk.

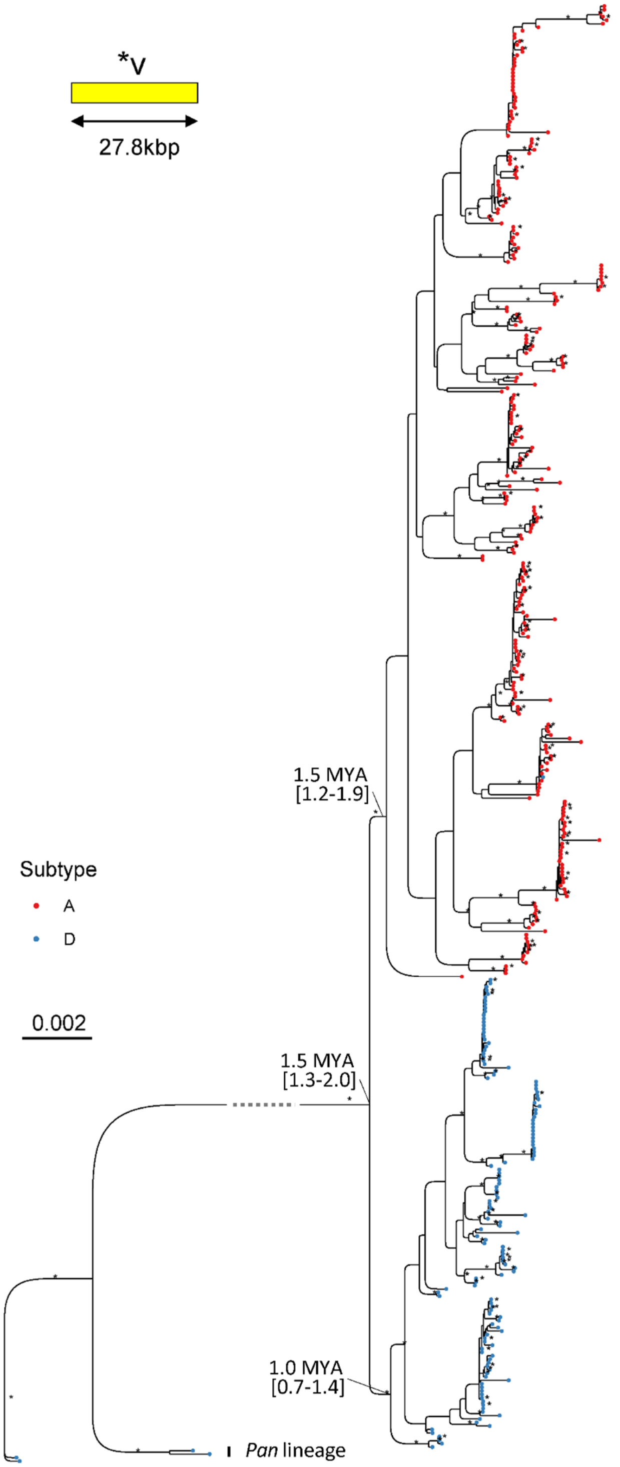

Supplementary Figure 26: Phylogenetic tree of duplicon (v) of length 28 kbp (n=416).
The tree was outgrouped with chimpanzee and bonobo (*Pan* lineage) orthologs. The human copies located in LCRA and D are indicated as red and blue, respectively. Notable divergence times among human copies are indicated as million years ago (MYA) with the 95% confidence interval. The bootstrap value of >95 is indicated as an asterisk.

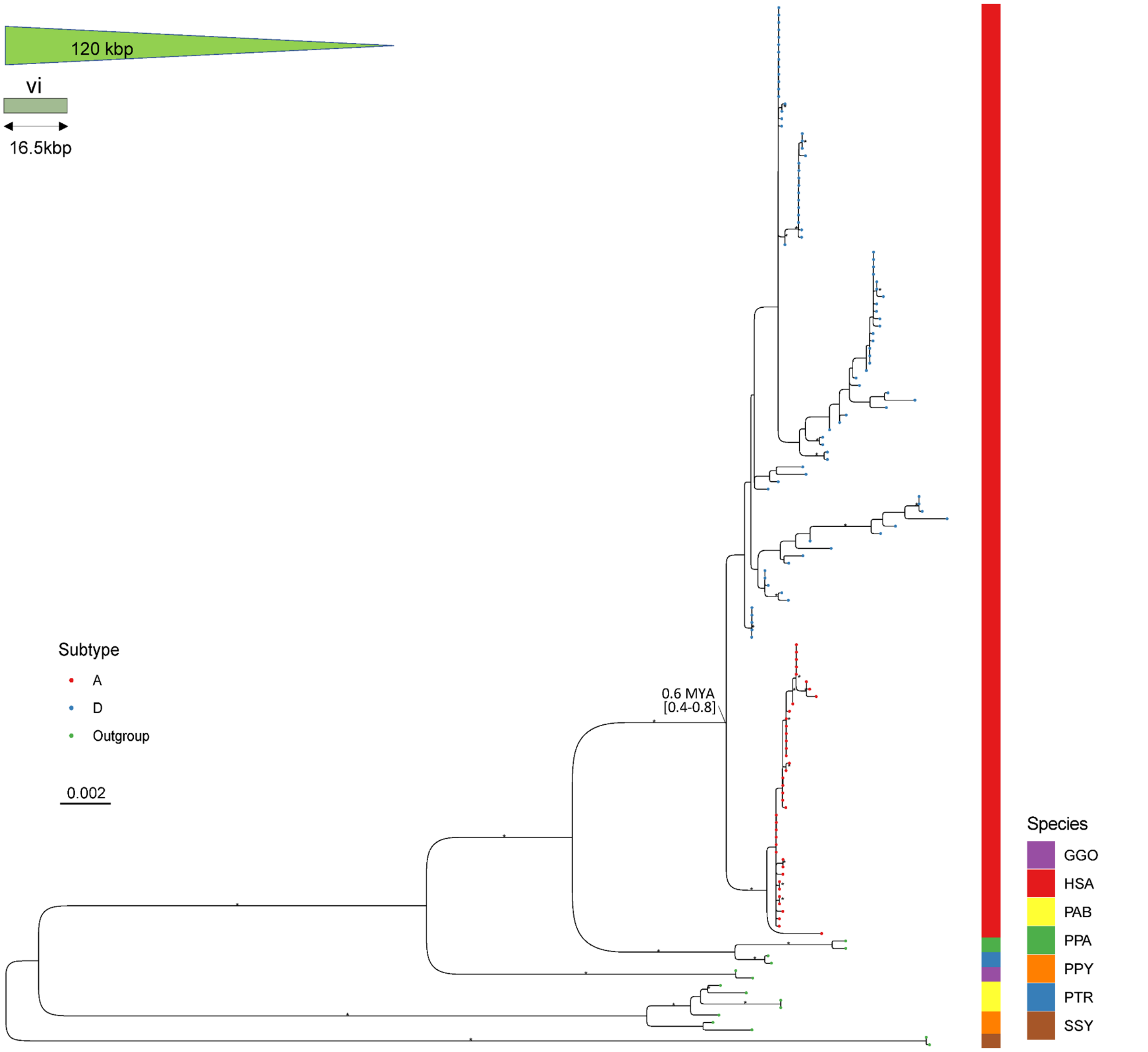

Supplementary Figure 27: Phylogenetic tree of duplicon (vi) of length 16.5 kbp (n=190).
The tree was outgrouped with single-copy macaque ortholog (absent in the tree). The human copies located in LCRA and D are indicated as red and blue, respectively. The corresponding species is indicated on the right annotation track; chimpanzee (PTR), bonobo (PPA), human (HSA), gorilla (GGO), Sumatran orangutan (PAB), Bornean orangutan (PPY) and siamang (SSY). Notable divergence times are indicated as million years ago (MYA) with the 95% confidence interval. The bootstrap value of >95 is indicated as an asterisk.

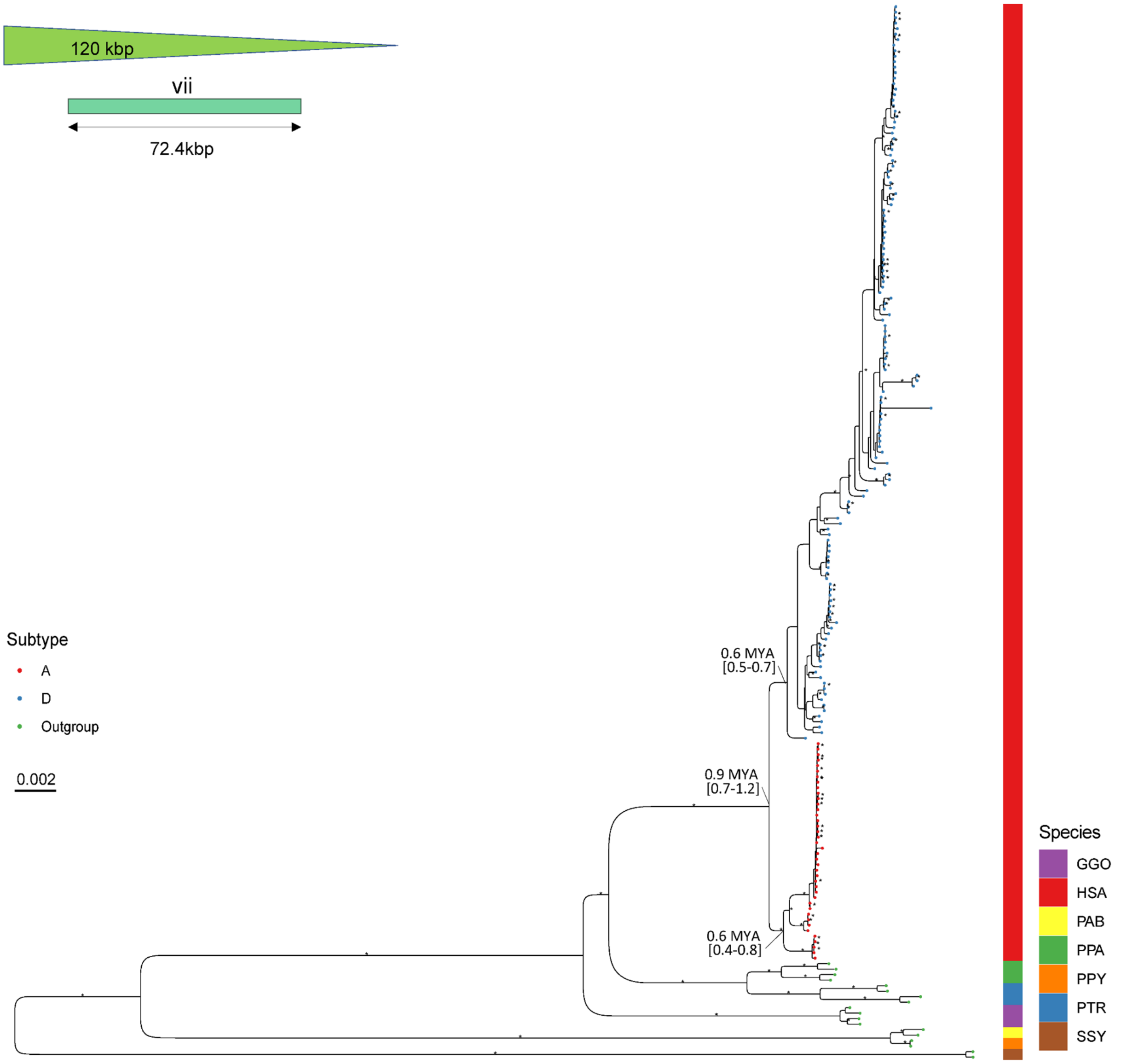

Supplementary Figure 28: Phylogenetic tree of duplicon (vii) of length 72.4 kbp (n=193).
The tree was outgrouped with single-copy macaque ortholog (absent in the tree). The human copies located in LCRA and D are indicated as red and blue, respectively. The corresponding species is indicated on the right annotation track; chimpanzee (PTR), bonobo (PPA), human (HSA), gorilla (GGO), Sumatran orangutan (PAB), Bornean orangutan (PPY) and siamang (SSY). Notable divergence times are indicated as million years ago (MYA) with the 95% confidence interval. The bootstrap value of >95 is indicated as an asterisk.

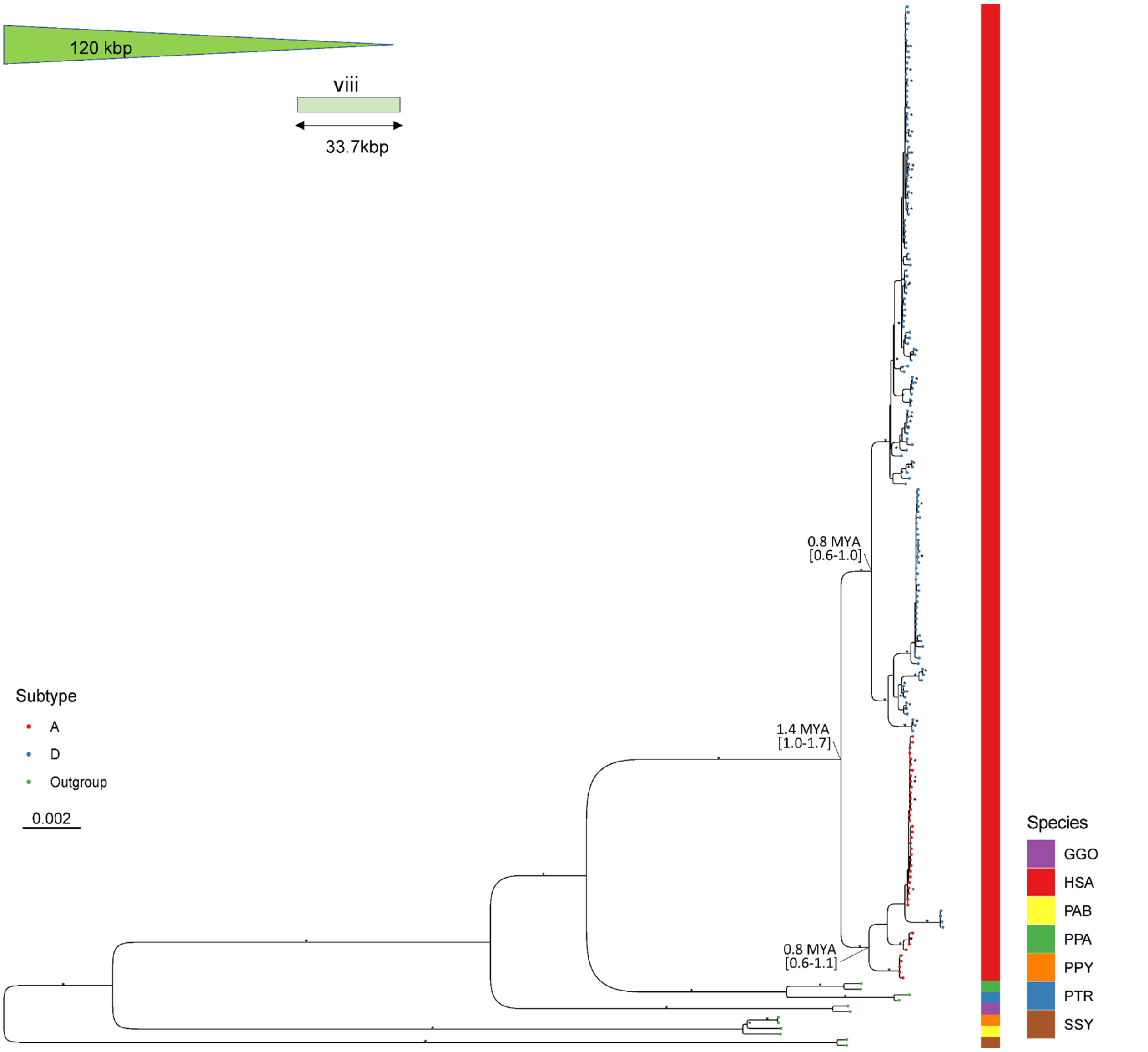

Supplementary Figure 29: Phylogenetic tree of duplicon (viii) of length 33.7 kbp (n=187). The tree was outgrouped with single-copy macaque ortholog (absent in the tree). The human copies located in LCRA and D are indicated as red and blue, respectively. The corresponding species is indicated on the right annotation track; chimpanzee (PTR), bonobo (PPA), human (HSA), gorilla (GGO), Sumatran orangutan (PAB), Bornean orangutan (PPY) and siamang (SSY). Notable divergence times are indicated as million years ago (MYA) with the 95% confidence interval. The bootstrap value of >95 is indicated as an asterisk.

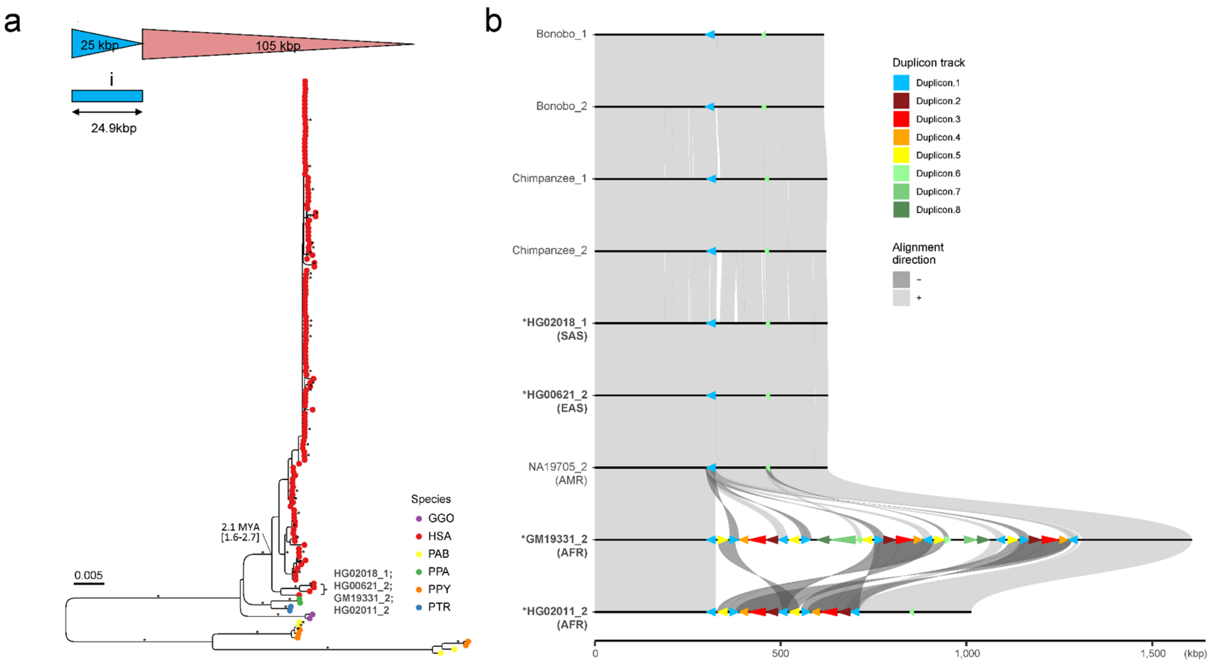

Supplementary Figure 30: Phylogenetic tree of the first proximal copy of duplicon (i) located in LCRA (n=150).

a) The tree was outgrouped with macaque and orangutan orthologs. For the outgroup orthologs, the copies located in LCRA are included. The corresponding species is indicated in different colors; chimpanzee (PTR), bonobo (PPA), gorilla (GGO), Bornean orangutan (PPY), Sumatran orangutan (PAB) and human (HSA). The deepest coalescent time among human copies is indicated as million years ago (MYA) with the 95% confidence interval. The bootstrap value of >95 is indicated as an asterisk. **b**) The first proximal copy of 25 kbp repeat unit located in LCRA among humans indicated by blue arrowhead. Duplicon structures of four putative ancestral haplotypes highlighted in the tree, including two haplotypes, HG02018_1 (haplotype 1) and HG00621_2 (haplotype 2), which show similar structure to chimpanzee and bonobo.

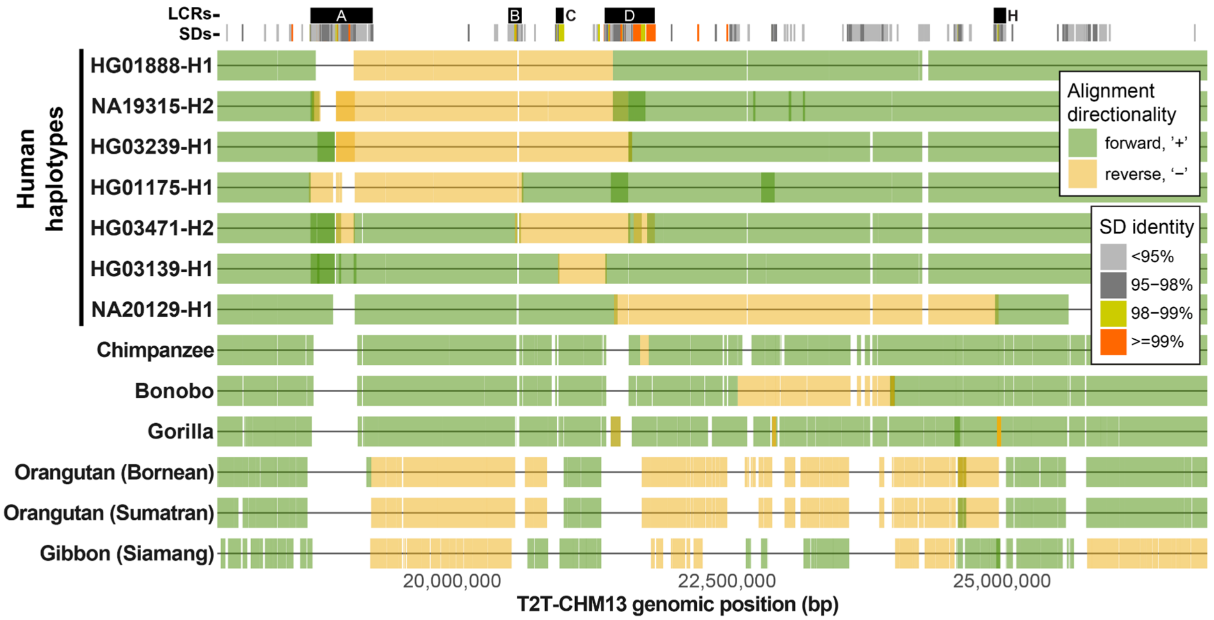

Supplementary Figure 31: Summary of large inversions detected at 22q11.2.

Alignment directionality of fully assembled sequences across the 22q11.2 region for example human haplotypes and nonhuman primates with respect to T2T-CHM13. Forward oriented alignments are shown in green while reverse

oriented alignments in yellow (designating large-scale inversions). On top, there is an SD annotation colored by sequence identity followed by annotation by LCRA-D and H as black rectangles.

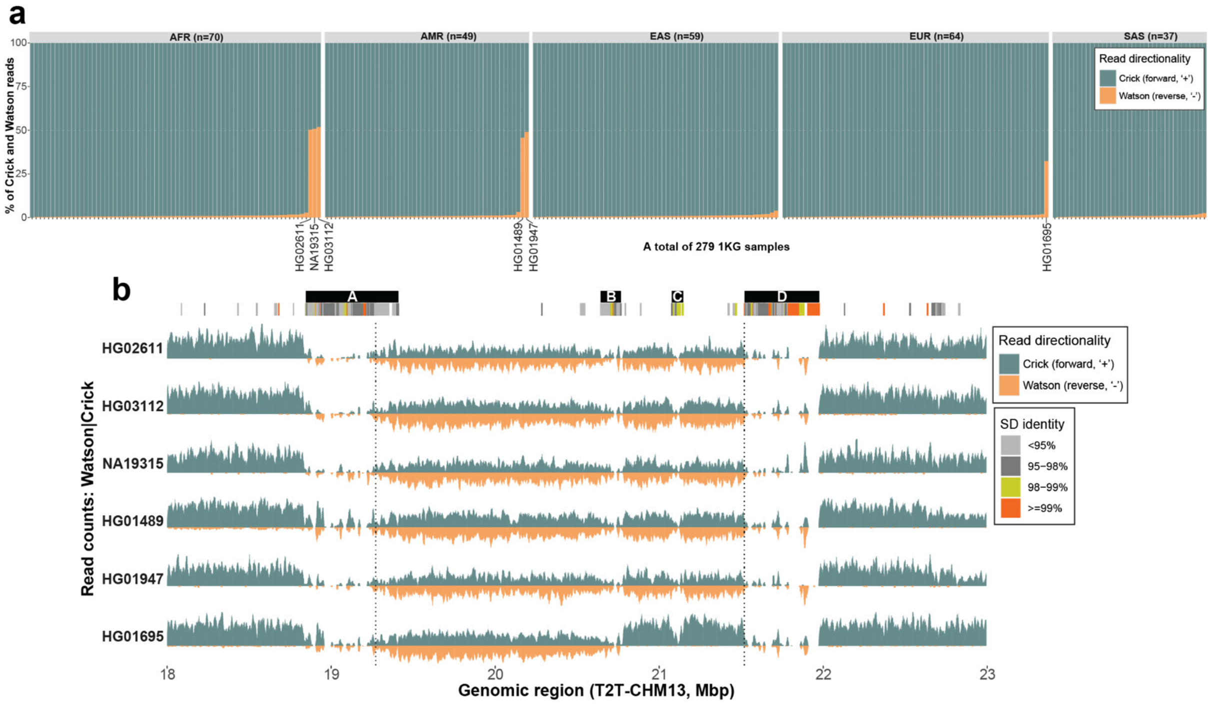

Supplementary Figure 32: Strand-seq-based inversion genotyping.
a) Barplot showing the proportions of forward (Crick - teal) and reverse (Watson - orange) mapping reads to the reference genome for 279 diverse human samples. Samples with about 50% proportion of Watson reads mark heterozygous inversion. Sample (HG01695) with a little lower Watson proportion due to a short A-B inversion.
b) Read-coverage profiles of Strand-seq data over the 22q11.2 region summarized as binned (bin size: 10 kbp step size: 1 kbp) read counts represented as bars above (teal; Crick read counts) and below (orange; Watson read counts) the midline. Dotted lines highlight the inversion region between LCRA and LCRD. Here, equal coverage of Watson and Crick counts represents a heterozygous inversion as only one homologue is inverted with respect to the reference (T2T-CHM13). Above there is an SD annotation and marked boundaries of SD blocks (A to D) as black rectangles.

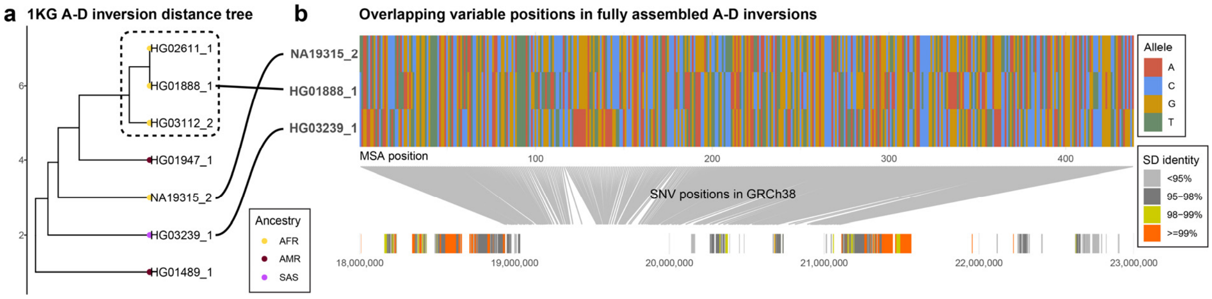

Supplementary Figure 33: Variable positions specific for each unique A-D inversion.
a) UPGMA distance tree distance tree constructed from phased SNVs using assemblies and Strand-seq data. Inversions that occurred on the same haplotype are marked in the dashed rectangle. **b**) Visualization of variable SNV positions among three fully assembled A-D inversions projected on GRCh38 reference.

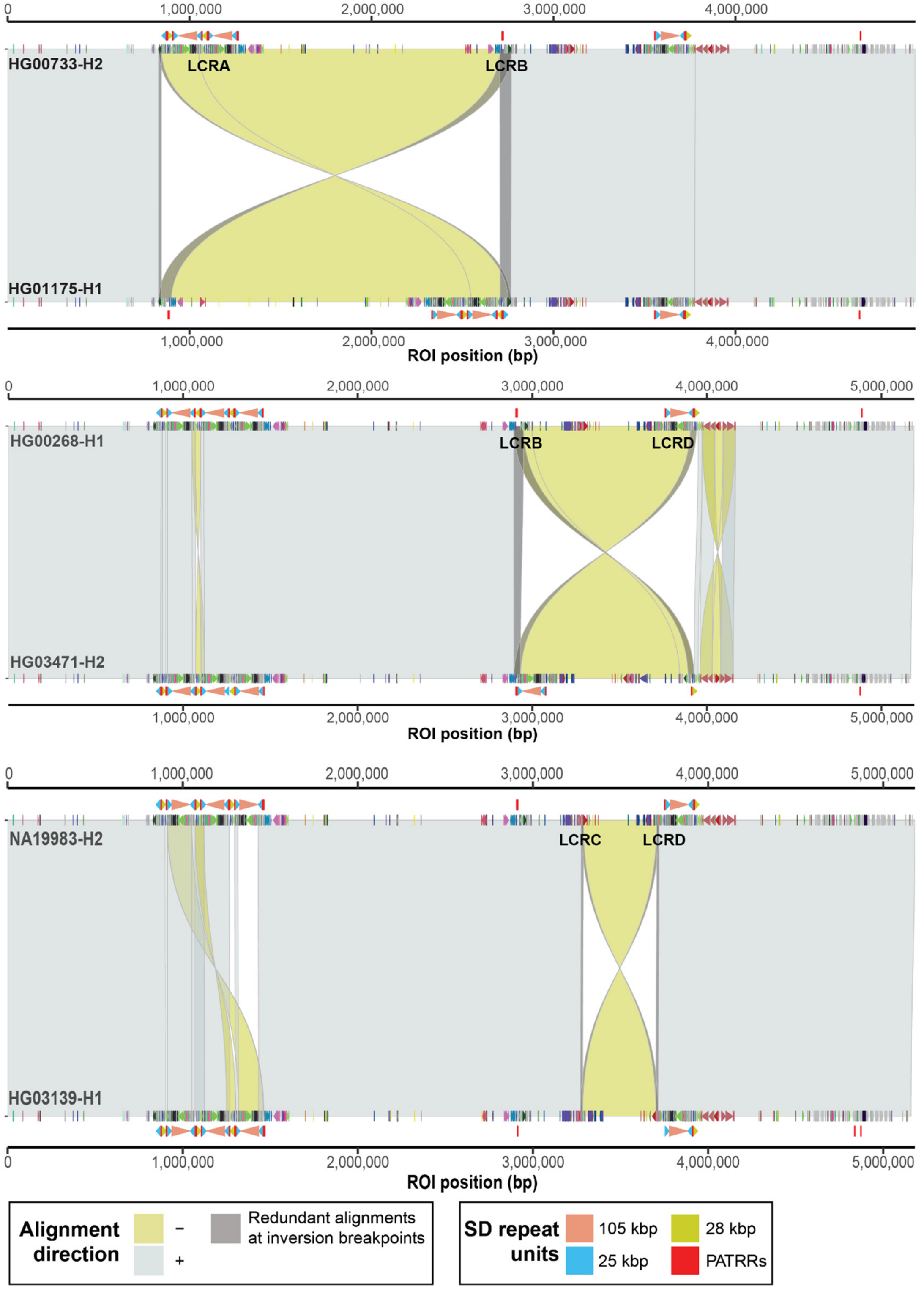

Supplementary Figure 34: Large-scale inversions mediated by various pairs of LCRA-to-D.
Miropeats-style plot showing the alignments (direct '+' - gray, inverted '-' - yellow) between direct (target at the top) and inverted (query at the bottom) haplotypes for inversions between LCRA-B, LCRB-D, and LCRC-D. There is a duplicon annotation specific to query and target sequences shown as directional arrowheads colored by unique duplicon ID. Positions of 105 (red), 25 (blue), and 28 (yellow) kbp long repeat units are shown below the query and above the target sequence along with PATRRs shown as red rectangles. Redundant (overlapping) alignments at the inversion boundaries are highlighted in dark gray color. ROI - region of interest (T2T-CHM13, chr22:18-23Mbp).

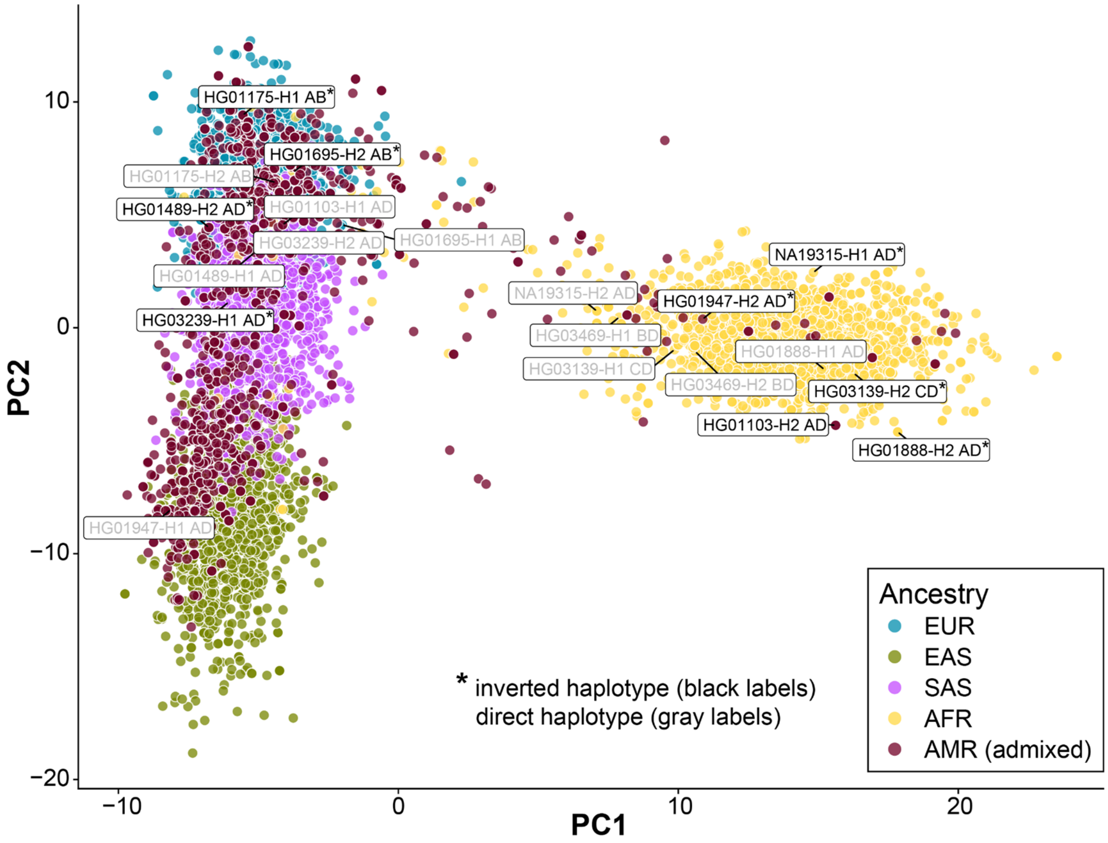

Supplementary Figure 35: Population ancestry of inverted haplotypes.
Ancestry-specific grouping based on PCA. Inverted haplotypes in the 1KG panel were marked based on the concordance with assembly- or Strand-seq-based phasing. Inverted haplotypes are highlighted using black samples labels with asterisks. Each inversion is marked by LCRs in between which it occurred (AB, BD, CD, and AD).

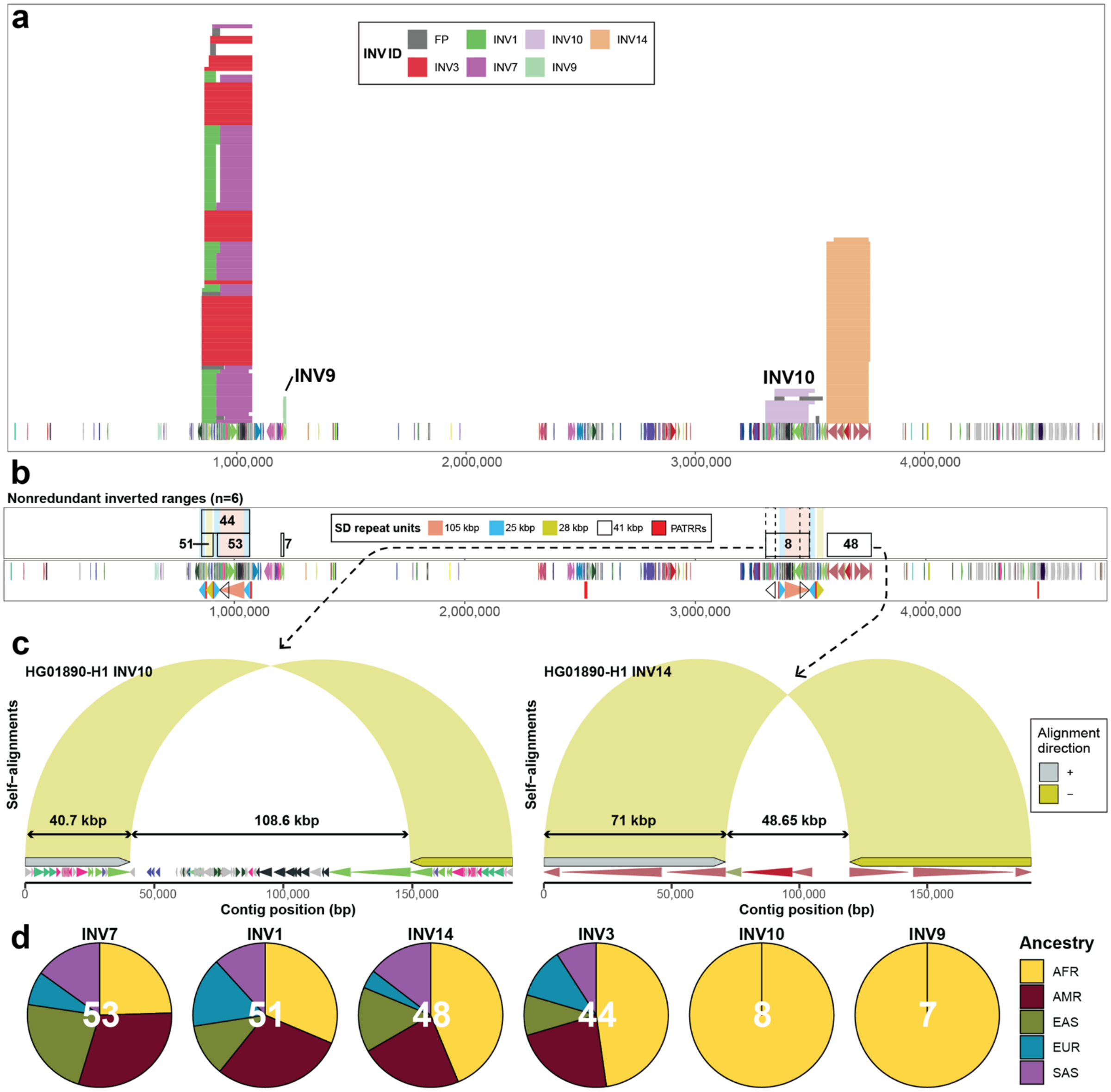

Supplementary Figure 36: Inversion detection within LCRA and D.
a) Visualization of all inverted ranges across all 133 human haplotypes with respect to a single human reference of African ancestry (HG01890-H1) with a single copy of the 105 kbp segment in both LCRA and D. Each inverted alignment defined among all 133 haplotypes is stacked on top of each other and is colored by an alignment group defined by 60% reciprocal overlap (**Methods**). At the bottom there is a duplicon annotation shown as directional arrowheads colored by a unique duplicon ID. **b**) Visualization of nonredundant inversion ranges defined for each alignment group from (a) (**Methods**). Each range is marked with the number that reports the number of inverted alignments in a given group. Below is a duplicon annotation shown as directional arrowheads colored by a unique duplicon ID as well as annotation of 105 (red), 25 (blue) and 28 (yellow) kbp long repeat units shown as directional arrowheads. We also show PATRR positions as red rectangles. **c**) Zoomed-in view of two inversions from LCRD showing the inverted repeats at their flanks (yellow-colored arcs). Below them we show duplicon annotation for a given region shown as directional arrowheads colored by a unique duplicon ID. We also highlight the predicted size of the inversion and its flanking repeats on top of two-directional arrows. **d**) Ancestry frequency for each of the six inverted duplications**.**

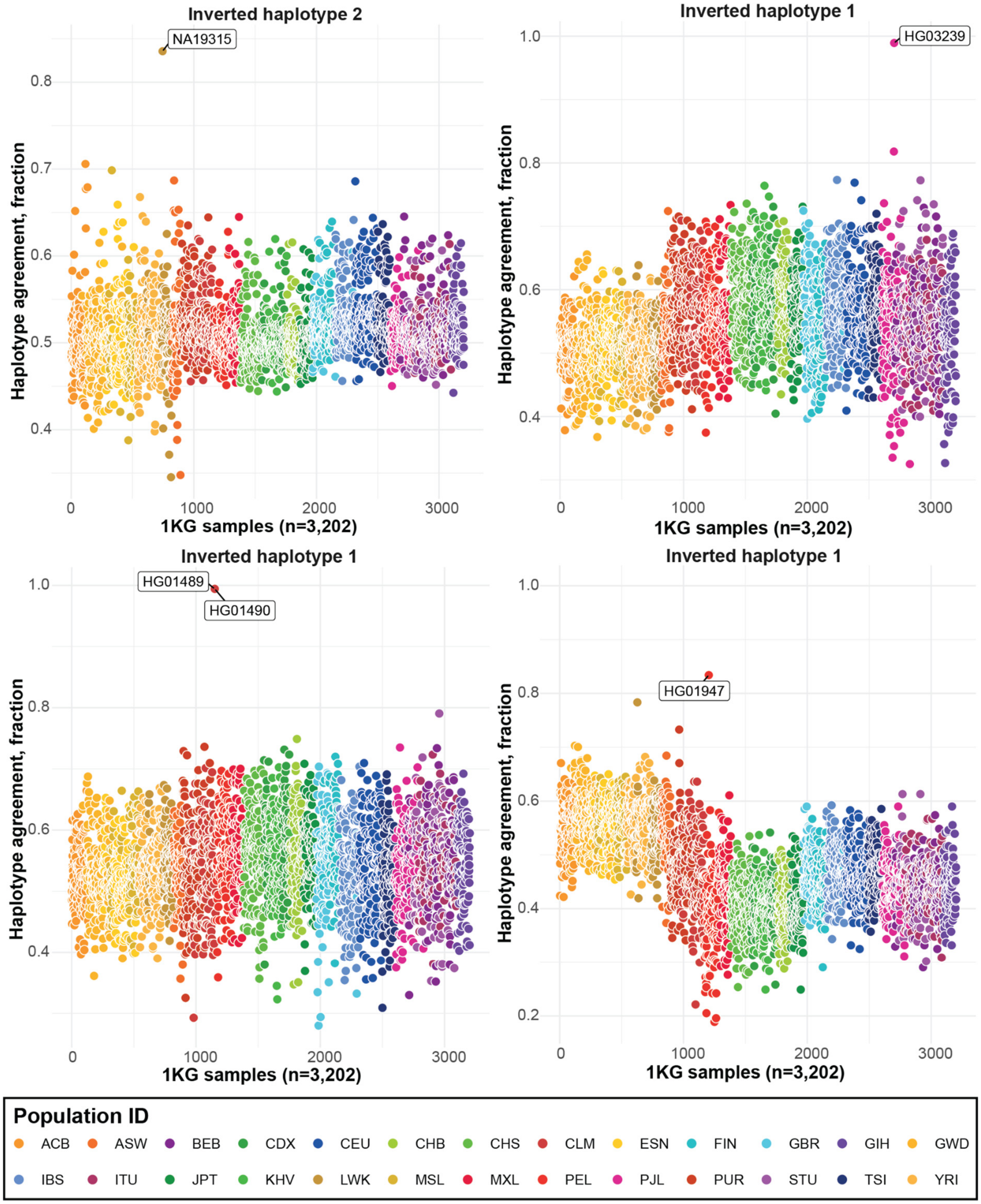

Supplementary Figure 37: LCRA-D inversion genotyping in NA19315, HG03239, HG01489, and HG01947. Haplotype agreement (y-axis, fraction of matching alleles) between inverted haplotype with respect to 1KG sample panel (n=3,202, GRCh38 coordinates). Each dot represents a single sample colored by a population ID of five major ancestries (African - shades of orange, American - shades of red, East Asian - shades of green, European - shades of blue, and Southeast Asian - shades of purple). Given the observed haplotype agreement between inverted haplotypes and 1KG samples, we do not report any putative inversion carrier among the 1KG panel.

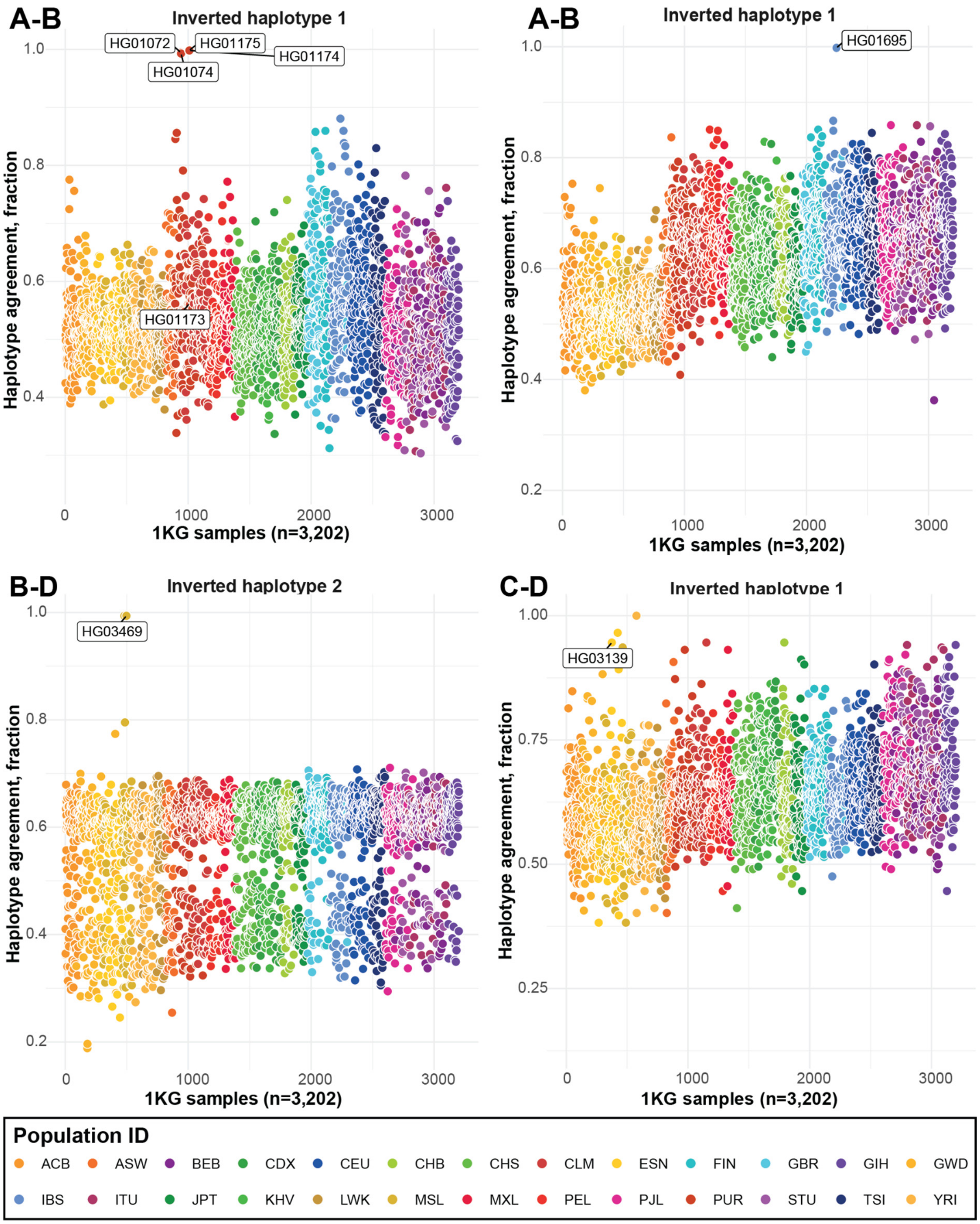

Supplementary Figure 38: LCRA-B, LCRB-D, and LCRC-D inversion genotyping.
Haplotype agreement (y-axis, fraction of matching alleles) between inverted haplotype with respect to 1KG sample panel (n=3,202, GRCh38 coordinates). Each dot represents a single sample colored by a population ID of five major ancestries (African - shades of orange, American - shades of red, East Asian - shades of green, European - shades of blue, and Southeast Asian - shades of purple).

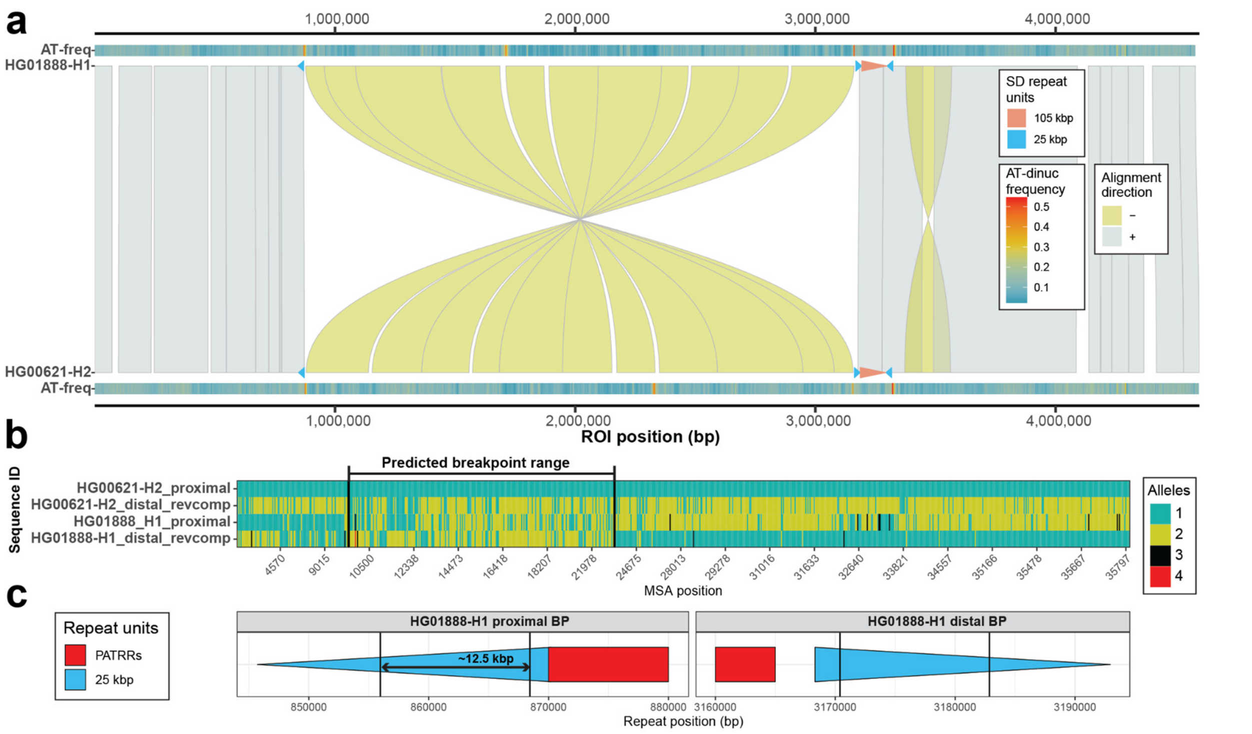

Supplementary Figure 39: Inversion breakpoint mapping in sample HG01888.

a) Miropeats-style plot showing nucmer alignments (direct - gray, '+' and inverted - yellow, '-') between query (bottom) and target (top). Query and target sequences were aligned using (mummer v4.0.0) and its function 'nucmer' with –mum parameter. Alignments of 105 (red) and 25 (blue) kbp long repeat units are shown on top of the query and target sequences. Below the query and above target there is a heatmap projecting the frequency of AT-dinucleotides ('AT-freq') in query and target sequences. **b**) MSA visualization between inversions flanking 25 kbp repeat units, including adjacent PATRRs from direct (HG00621-H2) and inverted (HG01888-H1) haplotypes. Paralog-specific variants (PSVs) from the proximal (dark green) and distal (dark yellow) LCRs are colored separately. Gaps in the MSA are colored white and PSVs not present in the proximal and distal LCRs are shown in black and red, respectively. Vertical solid lines depict detected change points where inverted haplotype (HG01888-H1) matches proximal PSVs (dark green) and then switches to match distal PSVs (dark yellow) of the direct haplotype (HG00621-H2) and vice versa. **c**) Position of the narrowed down breakpoint with respect to proximal (LCRA) and distal (LCRD) 25 kbp repeat unit. ROI - region of interest (T2T-CHM13, chr22:18-23Mbp).

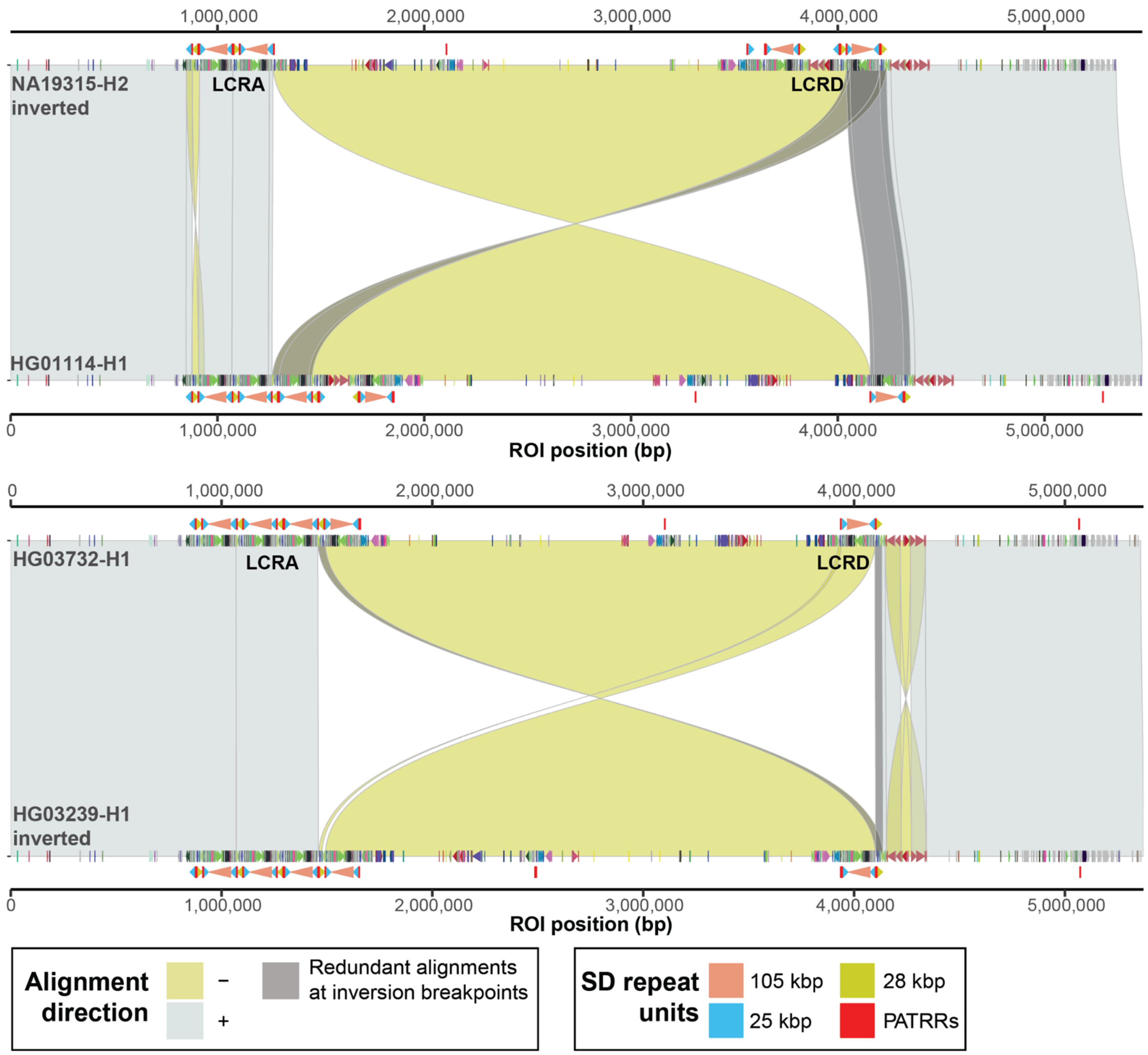

Supplementary Figure 40: A-D inversions in sample NA19315-H2 and HG03732-H1.
Miropeats-style plot showing the alignments (direct '+' - gray, inverted '-' - yellow) between direct and inverted (marked as so in the plot) haplotypes for A-to-D inversions in sample NA19315-H2 and HG03239-H1. There is a duplicon annotation specific to query and target sequences shown as directional arrowheads colored by unique duplicon ID. Positions of 105 (red), 25 (blue), and 28 (yellow) kbp long repeat units are shown below the query and above the target sequence along with PATRRs shown as red rectangles. Redundant (overlapping) alignments at the inversion boundaries are highlighted in dark gray color. ROI - region of interest (T2T-CHM13, chr22:18-23Mbp).

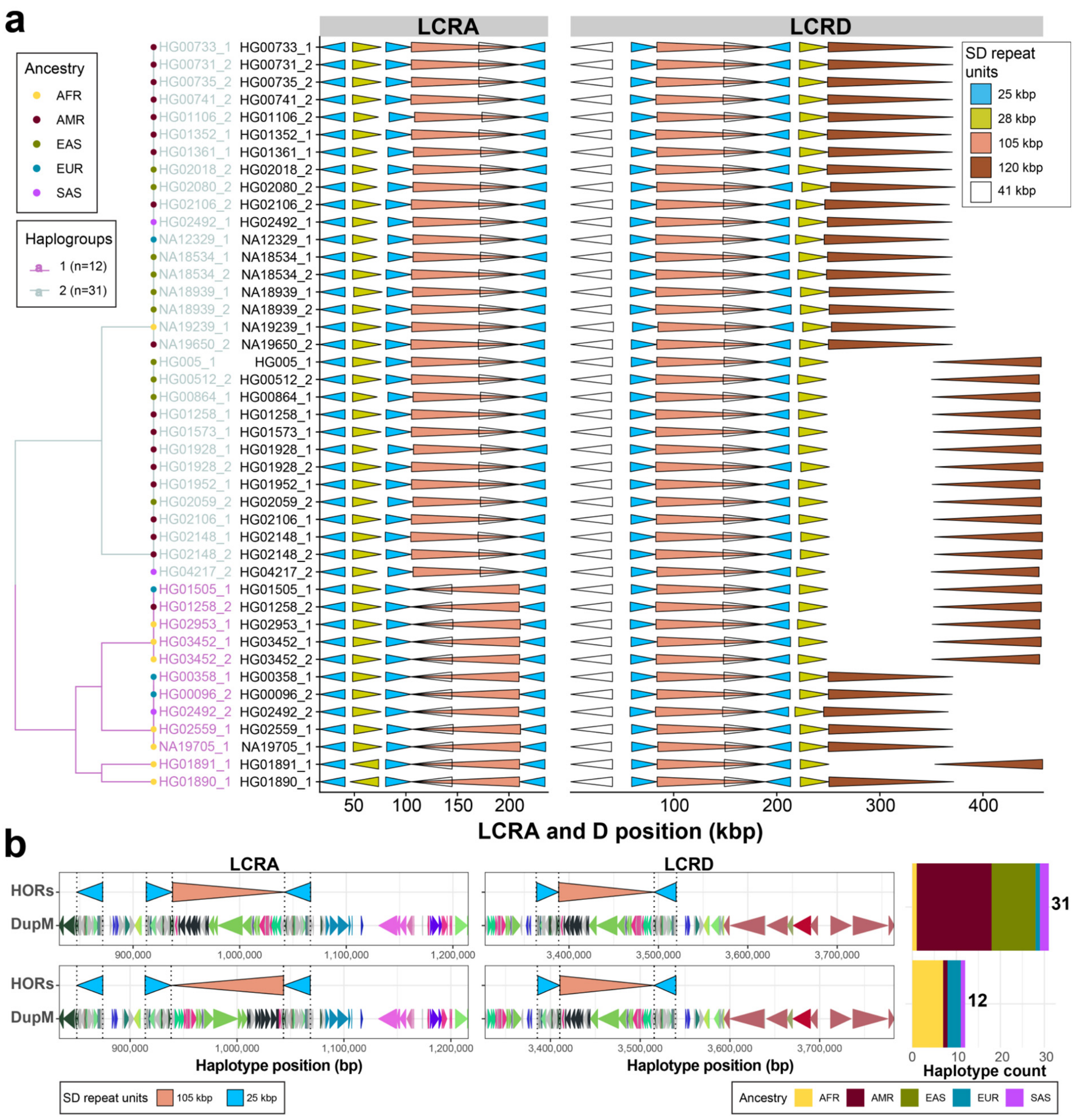

Supplementary Figure 41: Haplotypes with a single copy of 105 kbp segment in LCRA and D (n=43).
a) Left: UPGMA tree constructed based on a distance calculated all pairs of haplotypes (n=43) using their higher-order repeat (HOR) structure. The tree is cut into two major haplogroups ('1' - purple; '2' - gray) and each haplotype is marked by a colored point marking its ancestry. Right: Visualization of HOR annotation for all 43 haplotypes clustered by the UPGMA tree with colored repeat units (see legend). **b**) Summary of all human haplotypes (n=43) with a single copy of 105 kbp repeat unit (red) in both LCRA and D. We show an annotation of 105 (red) and 25 (blue) kbp long SD repeat units (or HORs) as directional arrowheads. Below there is a DupMasker annotation shown as directional arrowheads colored by a unique duplicon ID. Left: there is a stacked barplot showing the haplotype count for the forward (n=31) and reverse (n=12) oriented 105 kbp repeat unit between LCRA and D.

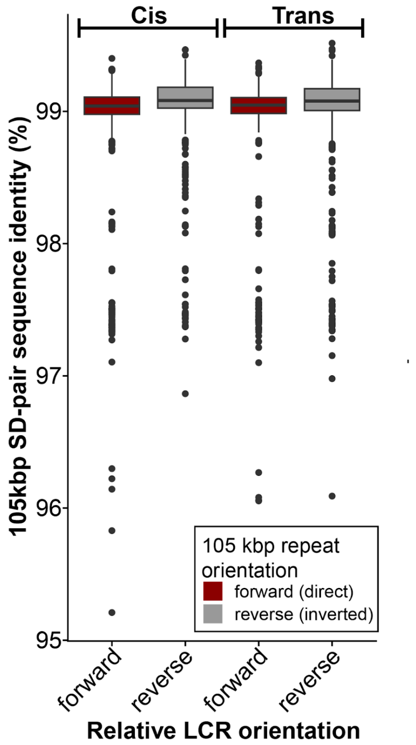

Supplementary Figure 42: Sequence identity of the 105 kbp repeat pairs between LCRA and D.
The boxplot shows the overall sequence identity for all intra (cis) and interchromosomal (trans) pairs of the 105 kbp repeat unit between LCRA and D. Overall reverse-oriented (gray) LCRA and D pairs have median identity 99.1% while those in forward orientation (dark red) have median identity 99%. Boxes represent interquartile range (IQR), including median line; whiskers extend to 25% − 1.5 × IQR and 75% + 1.5 × IQR; outliers are shown as black dots.

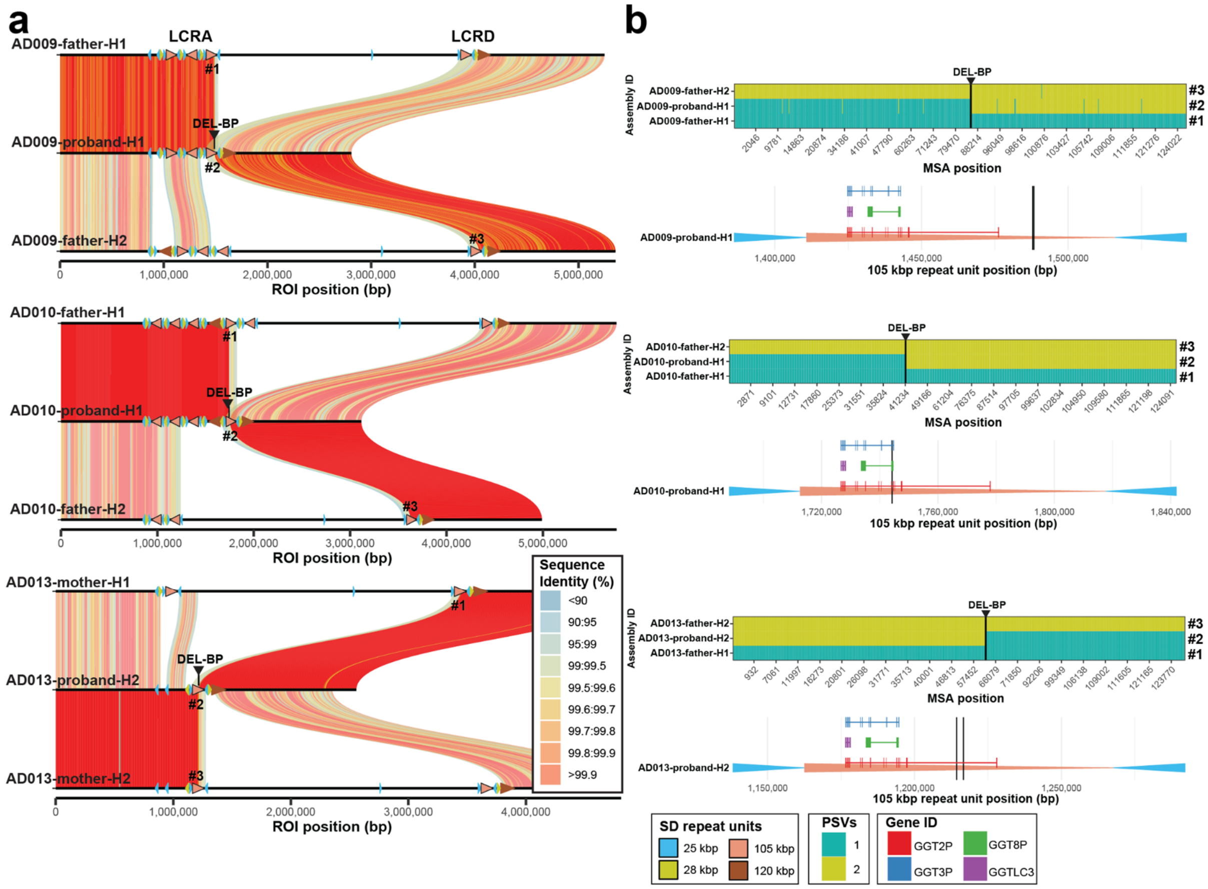

Supplementary Figure 43: Mapping of A-D deletion breakpoints in families 1-3.
a) Visualization of binned (bin size: 10 kbp) sequence identity between probands (middle) and both haplotypes (H1 and H2) of transmitting parents (top and bottom). Alignments are colored by sequence identity and likely inherited portions of parental haplotypes in the proband are highlighted by fully colored alignments (darker shades of red) while others are shown with 50% transparency. On top of each haplotype is an SD repeat annotation depicted as arrowheads colored by repeat units. Refined deletion breakpoints within the 105 kbp repeat units are marked by black arrowheads. We also mark 105 kbp repeat units (#1-3) used to narrow down the deletion breakpoints in panel b. **b**) MSA visualization between the 105 kbp SD repeat units (#1-3) extracted from probands and both parental haplotypes. Only PSVs (1 - green and 2 - yellow) are shown. Vertical solid lines depict detected change points where proband haplotype switches from matching H2 to H1 of the transmitting parent, or vice versa. Below there is a zoomed-in view of the repeat structure at the narrowed down deletion breakpoint. Duplicons are shown as colored arrowheads (105 kbp - light red and 25 kbp - blue). Mapped exons of the GGT gene family are shown and colored by gene ID (see legend). The predicted breakpoint is shown as a vertical black line. ROI - region of interest (T2T-CHM13, chr22:18-23Mbp).

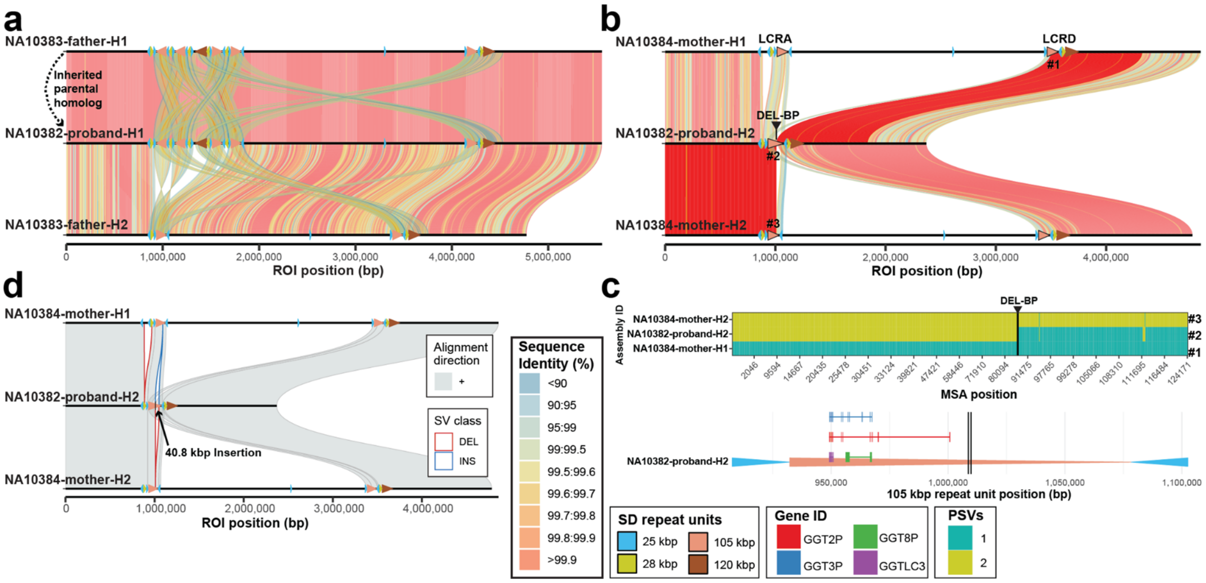

Supplementary Figure 44: Mapping of A-D deletion within a family trio.

a) Visualization of binned (bin size: 10 kbp) sequence identity between proband (NA10382) haplotype 1 (H1) with respect to both haplotypes (H1 and H2) of NA10383 (father). Proband haplotype (H1) is in the middle while parental haplotypes H1 and H2 are at the top and bottom, respectively. Alignments are colored by sequence identity. Given this, we conclude that proband H1 (NA10382-H1) is inherited from father (NA10383-H1) while the proband haplotype carrying the A-D deletion is inherited from both maternal haplotypes (NA10384 H1 and H2). **b**) Same as in panel a we show the sequence identity between proband (NA10382) haplotype 2 (H2) with respect to both haplotypes (H1 and H2) of transmitting parent (NA10384, mother). The proband haplotype is in the middle while parental haplotypes H1 and H2 are at the top and bottom, respectively. Alignments are colored by sequence identity and likely inherited portions of parental haplotypes in the proband are highlighted by fully colored alignments (darker shades of red) while others are shown with 50% transparency. On top of each haplotype is an SD repeat annotation depicted as arrowheads colored by repeat units. Refined deletion breakpoints within the 105 kbp repeat units are marked by black arrowheads. We also mark 105 kbp repeat units (#1-3) used to narrow down the deletion breakpoint in panel c. **c**) MSA visualization between the 105 kbp SD repeat units (#1-3) extracted from probands and both parental haplotypes. Only PSVs (1 - green and 2 - yellow) are shown. Vertical solid lines depict detected change points where proband haplotype switches from matching H2 to H1 of the mother. Below there is a zoomed-in view of the repeat structure at the narrowed down deletion breakpoint. Duplicons are shown as colored arrowheads (105 kbp - light red and 25 kbp - blue). Mapped exons of the GGT gene family are shown and colored by gene ID (see legend). The predicted breakpoint is shown as a vertical black line. **d**) Miropeats-style plot showing alignments (direct - gray, '+') between proband (NA10382) haplotype 2 (H2, middle) with respect to both haplotypes (H1 and H2; top and bottom) of NA10384 (mother). On top of each haplotype is an SD repeat annotation depicted as arrowheads colored by repeat units. The inserted sequence (40.8 kbp) within the 105 kbp repeat unit at the deletion breakpoint is highlighted by blue and red outlines (see back arrow). ROI - region of interest (T2T-CHM13, chr22:18-23Mbp).

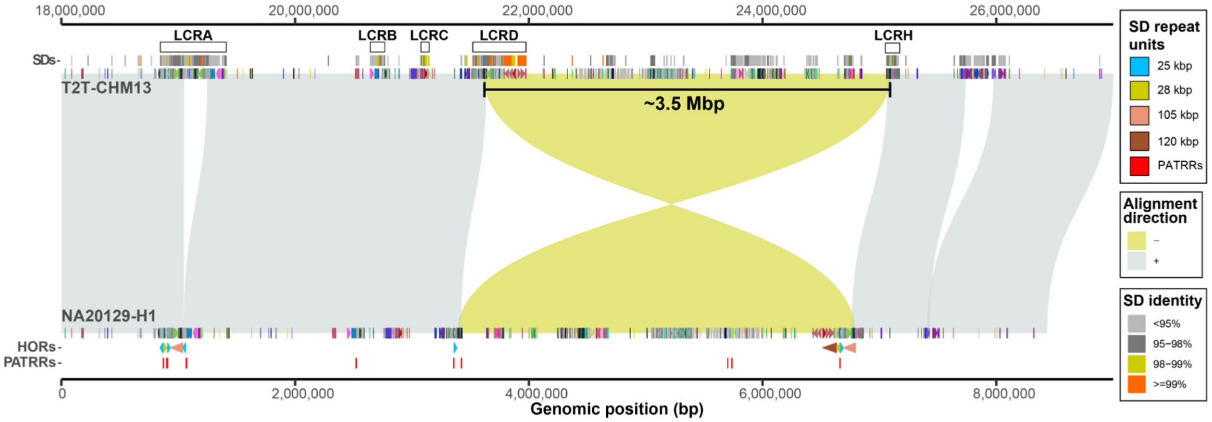

Supplementary Figure 45: Large-scale inversion between LCRD and distal LCR.
Miropeats-style plot showing the alignments (direct '+' - gray, inverted '-' - yellow) between direct (T2T-CHM13 reference) and inverted haplotypes (NA20129-H1) for large 3.5 Mbp inversion that extends from LCRD to a downstream located breakpoint. This inversion is heterozygous, present on haplotype 1 only. There is a duplicon annotation specific to query and target sequences shown as directional arrowheads colored by unique duplicon ID. Positions of 105 (red), 25 (blue), and 28 (yellow) kbp long SD repeat units (or HORs) are shown below the query sequence along with PATRRs shown as red rectangles. SD annotation of the target sequence is shown on top colored by sequence identity. Lastly, on top we show positions of LCRA-to-D and LCRH regions on the target sequence.

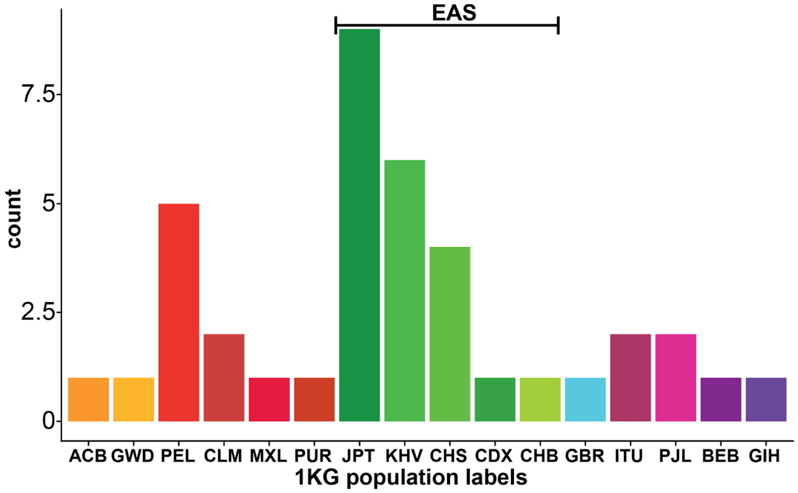

Supplementary Figure 46: Population labels of samples predicted to be 22q11.2DS susceptible.
The height of each bar shows the number of samples marked as predisposed to 22q11.2DS colored by population labels obtained from the 1KG project. The three letter abbreviations are 1KG-specific population labels (x-axis). Shades of green mark individuals of East-Asian ancestry (EAS).

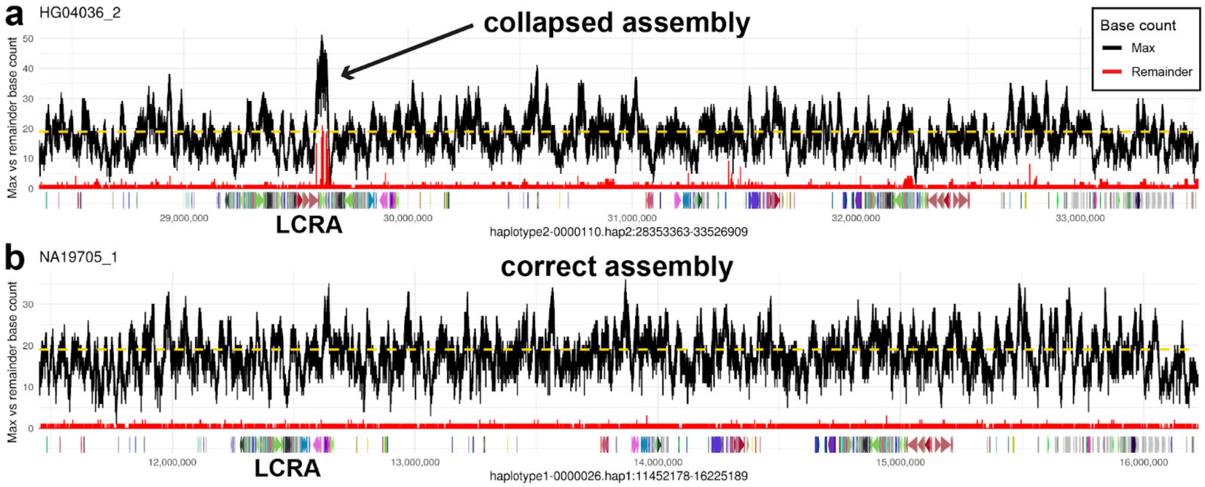

Supplementary Figure 47: Phased assembly validation using NucFreq.

Two examples of read-coverage profiles of aligned PacBio HiFi reads to the two phased genome assemblies of the 22q11.2 region (chr22:1800000-23000000 in T2T-CHM13 coordinates). **a**) A visualization of the most abundant base at each position of HG04036-H2 is shown in black. The second most abundant base is shown in red. Extended regions with observable increased coverage for the most abundant base (‘Max’) often coincide with increased frequency of the second (‘Remainder’) most abundant base (see arrow). Such regions are indicative of a collapsed assembly where not all copies of paralogous duplications are fully resolved. Misassemblies would be represented as an absence of sequence reads mapping across a region. **b**) This visualization shows the same frequency of the most and second most abundant bases along the haplotype from sample NA19705-H1. In this case we consider this assembly to be complete and correct as there are no extended regions of increased frequency of the secondary base (‘Remainder’ base).

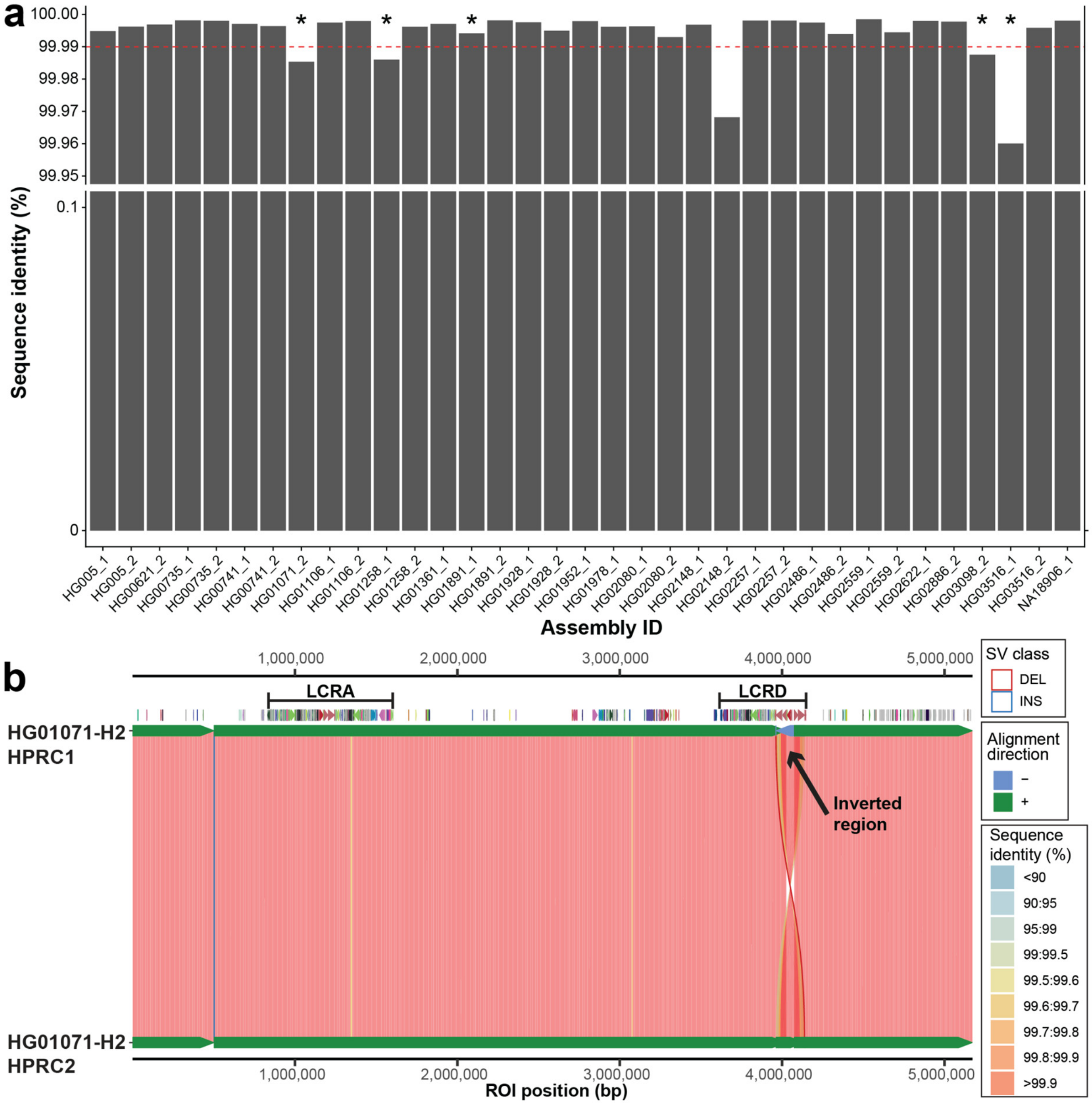

Supplementary Figure 48: Comparison of HPRC1 and HPRC2 assemblies for the same haplotypes.
a) A barplot showing the percent agreement between HPRC1 (release 1) and HPRC2 (release 2) assemblies generated using PacBio HiFi reads only for HPRC1 and both HiFi and ONT reads for HPRC2 assemblies. Haplotypes marked with an asterisk are those where we observed a distal inversion within LCRD (see example in panel b).
b) Visualization of binned (bin size: 10 kbp) sequence identity between the HPRC2 assembly for samples HG01071-H2 aligned to the HPRC1 assembly of the same haplotype. Alignments are colored by sequence identity. On top of the HPRC1 assembly there is a DupMasker annotation shown as colored arrowheads. Continuous LCRA and D regions are also highlighted. There is also a structural variant (SV, ≥50 bp) colored by blue and red outlines for insertions (INS) and deletions (DEL), respectively. Lastly, we mark an inverted region between the HPRC1 and HPRC2 assemblies visible in the distal region of LCRD. ROI - region of interest (T2T-CHM13, chr22:18-23Mbp).

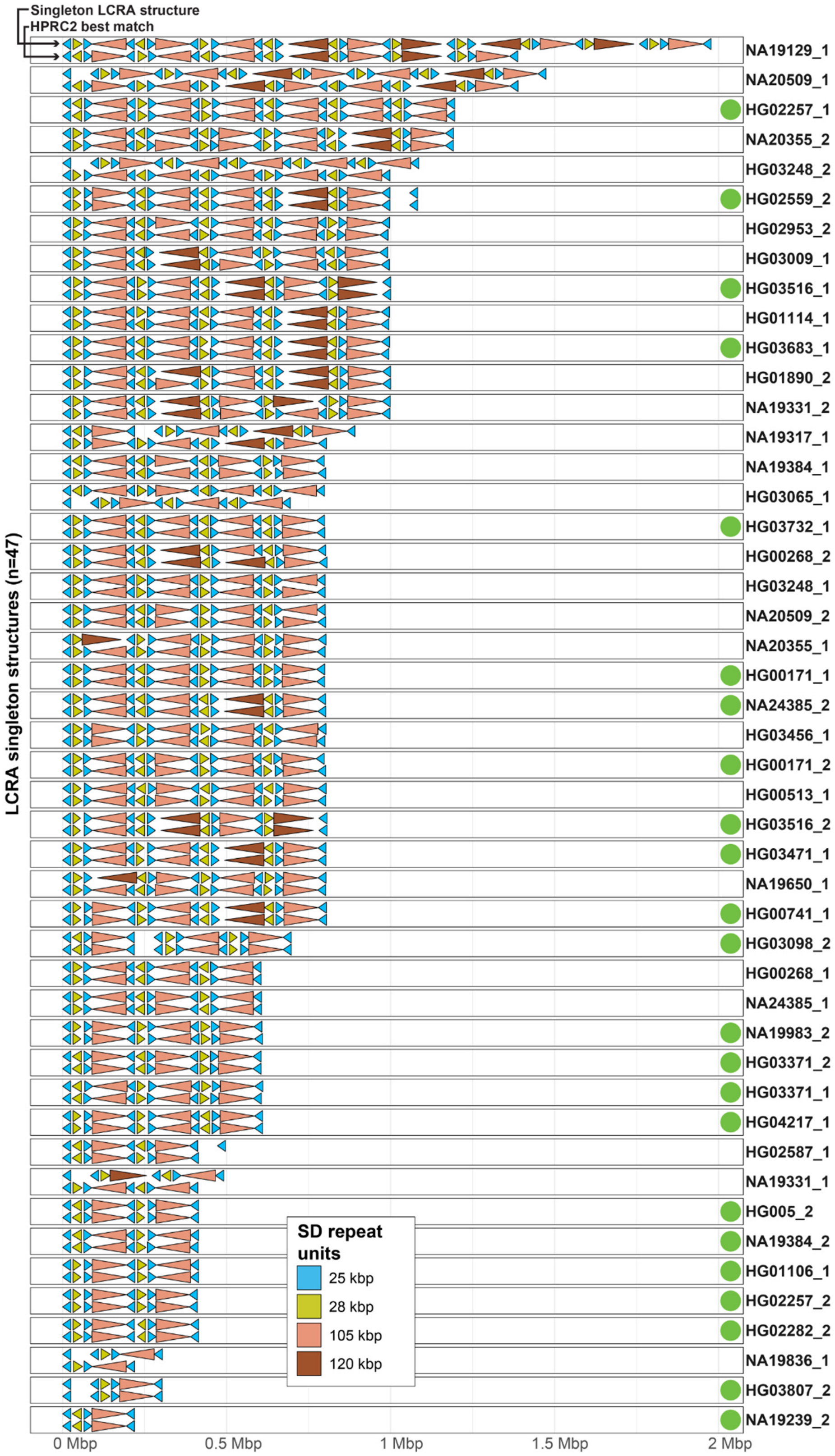

Supplementary Figure 49: Validation of singleton LCRA structures using HPRC2 (release 2) assemblies. Singleton LCRA structures (n=47) observed among 133 noninverted haplotypes are presented in each row (always on the top). Then, for each singleton haplotype, we present the closest haplotype structure observed among HPRC2 assemblies (always at the bottom). Each haplotype structure is presented as directional arrowheads colored by a SD

repeat unit (120 kbp, 105 kbp, 25 kbp, and 28 kbp; see legend; 41 kbp repeat unit not shown for simplicity). Singleton haplotypes whose exact structure was observed among HPRC2 samples are marked by green points.

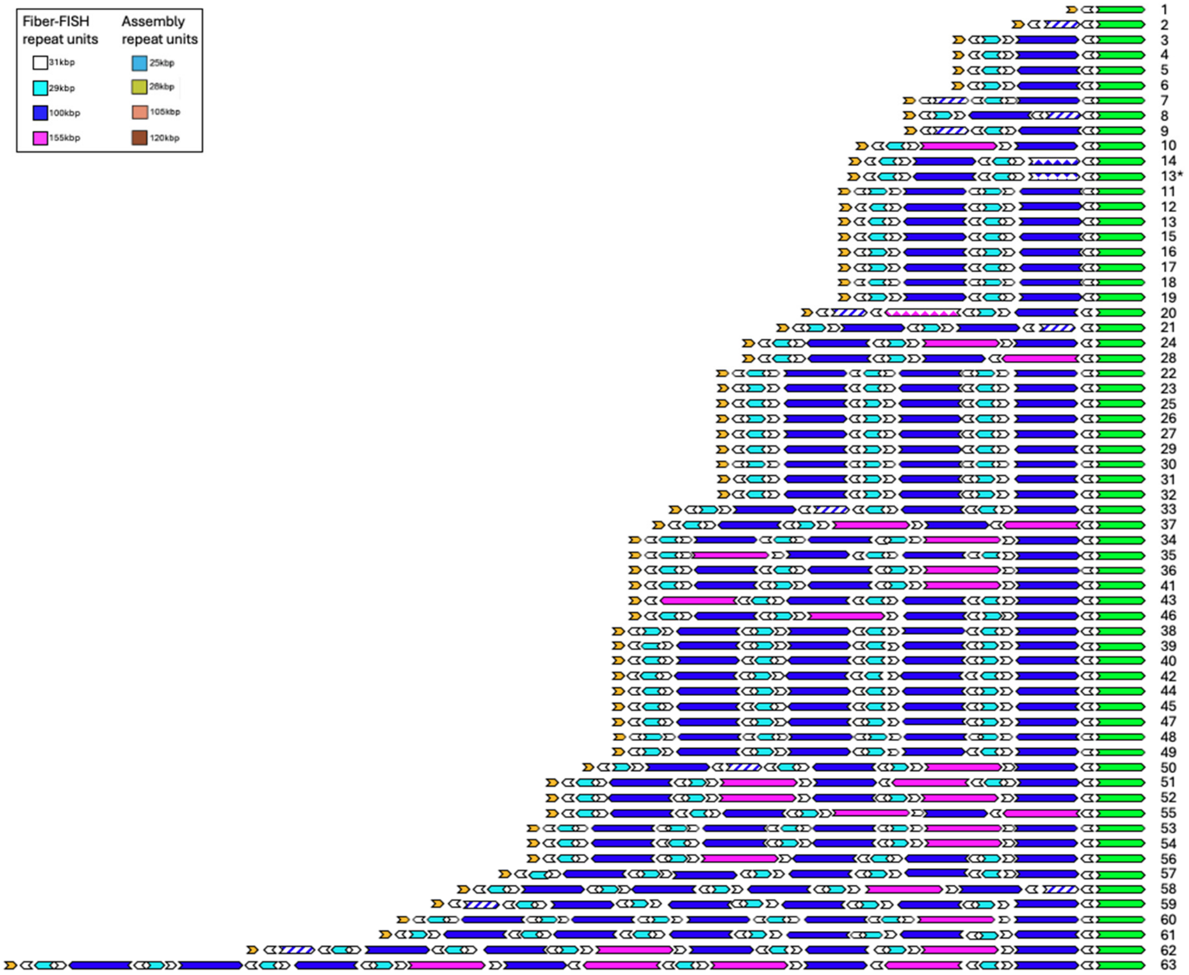

Supplementary Figure 50: Representation of LCRA assemblies according to fiber-FISH duplicons.
Each haplotype has been decomposed in duplicons based on its fiber-FISH probe composition. The yellow, cyan, magenta, blue, green and white duplicons are 25 kbp, 29 kbp, 155 kbp, 100 kbp, 80 kbp and 31 kbp, respectively[3]. LCRA structures defined by fiber-FISH duplicons and assembly are very similar in content, size, and orientation. The corresponding assembly haplogroup is given on the right. The assembly-based SD repeat units, defined in this manuscript, blue (25 kbp), yellow (28 kbp), light red (105 kbp) and brown (120 kbp) match the white (31 kbp), cyan (29 kbp), blue (100 kbp) and magenta (155 kbp) (see the legend). The fiber-FISH-based duplicon with blue stripes matches the 41 kbp duplicon defined within our assemblies (**Fig. 2c**).

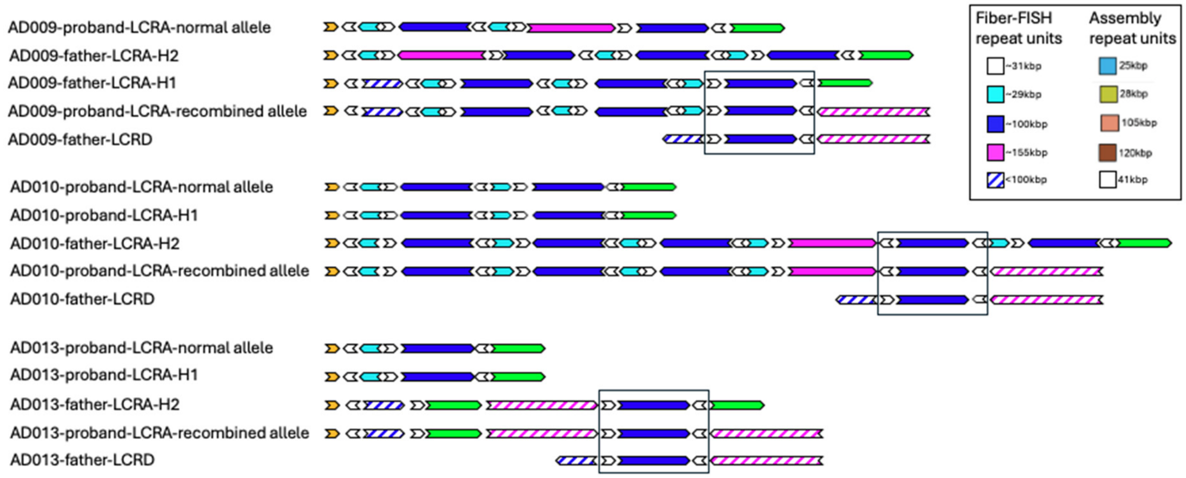

Supplementary Figure 51: Validation of 22q11.2DS families using fiber-FISH.
LCRA and LCRD alleles of proband and parent of origin are represented using the SD repeat units as published by Demaerel et al. (2019)[3]. The region in which the breakpoint occurred is shown for each duo in a black box. The assembled haplotypes have been decomposed in duplicons based on the fiber-FISH probe composition. All haplotypes matched the fiber-FISH defined structure.

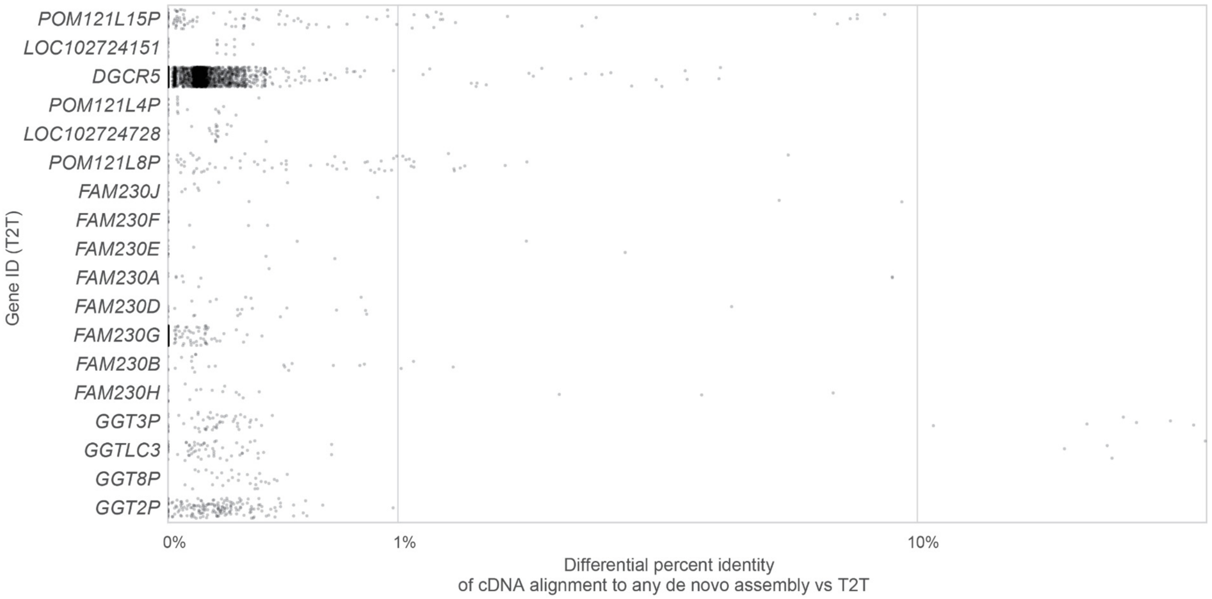

Supplementary Figure 52: Improved alignment of cDNA reads to population assemblies.
The percent identity differential of the mapping of full-length Iso-Seq transcripts from chr22q11.2 *POM121*, *FAM230*, and *GGT* paralogs to T2T-CHM13 compared to population assemblies. Each point represents an individual Iso-Seq read, and the x-axis indicates the improvement in alignment identity using population assemblies instead of the T2T-CHM13 reference.

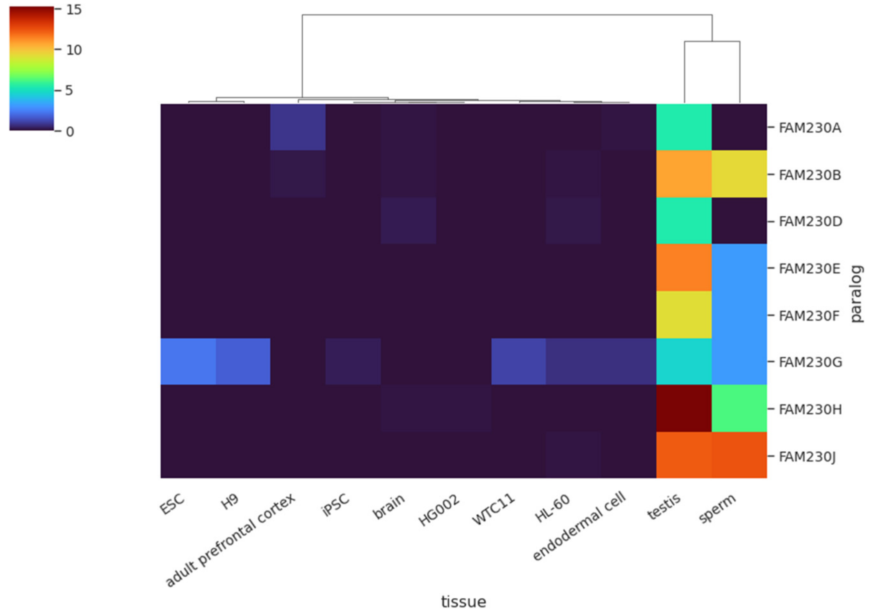

Supplementary Figure 53: Tissue specificity of Iso-Seq expression for *FAM230* paralogs.
Estimated Iso-Seq expression of *FAM230* paralogs as defined in T2T-CHM13, in reads per million. Only tissues/cell types with at least ten reads from the *FAM230* family are shown.

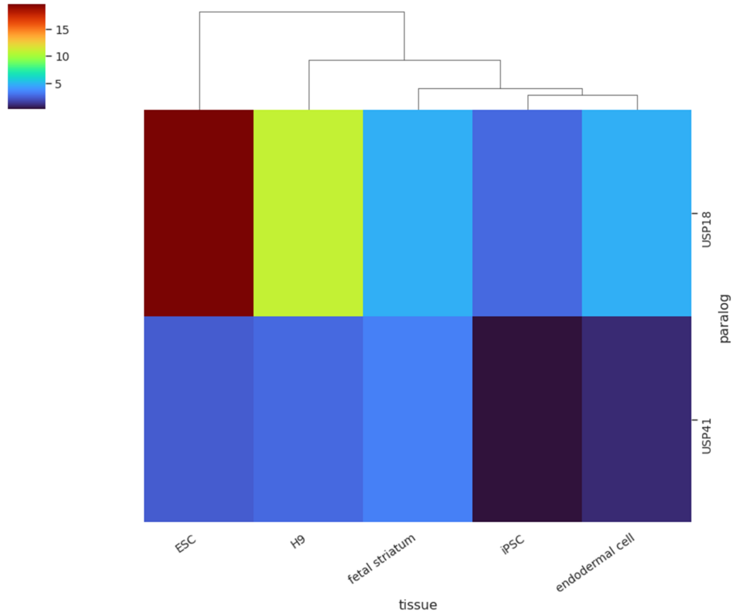

Supplementary Figure 54: Tissue specificity of Iso-Seq expression for *USP18* and *USP41*. Estimated Iso-Seq expression of *USP18/41* paralogs as defined in T2T-CHM13, in reads per million. Only tissues/cell types with at least ten reads from *USP41* are shown.

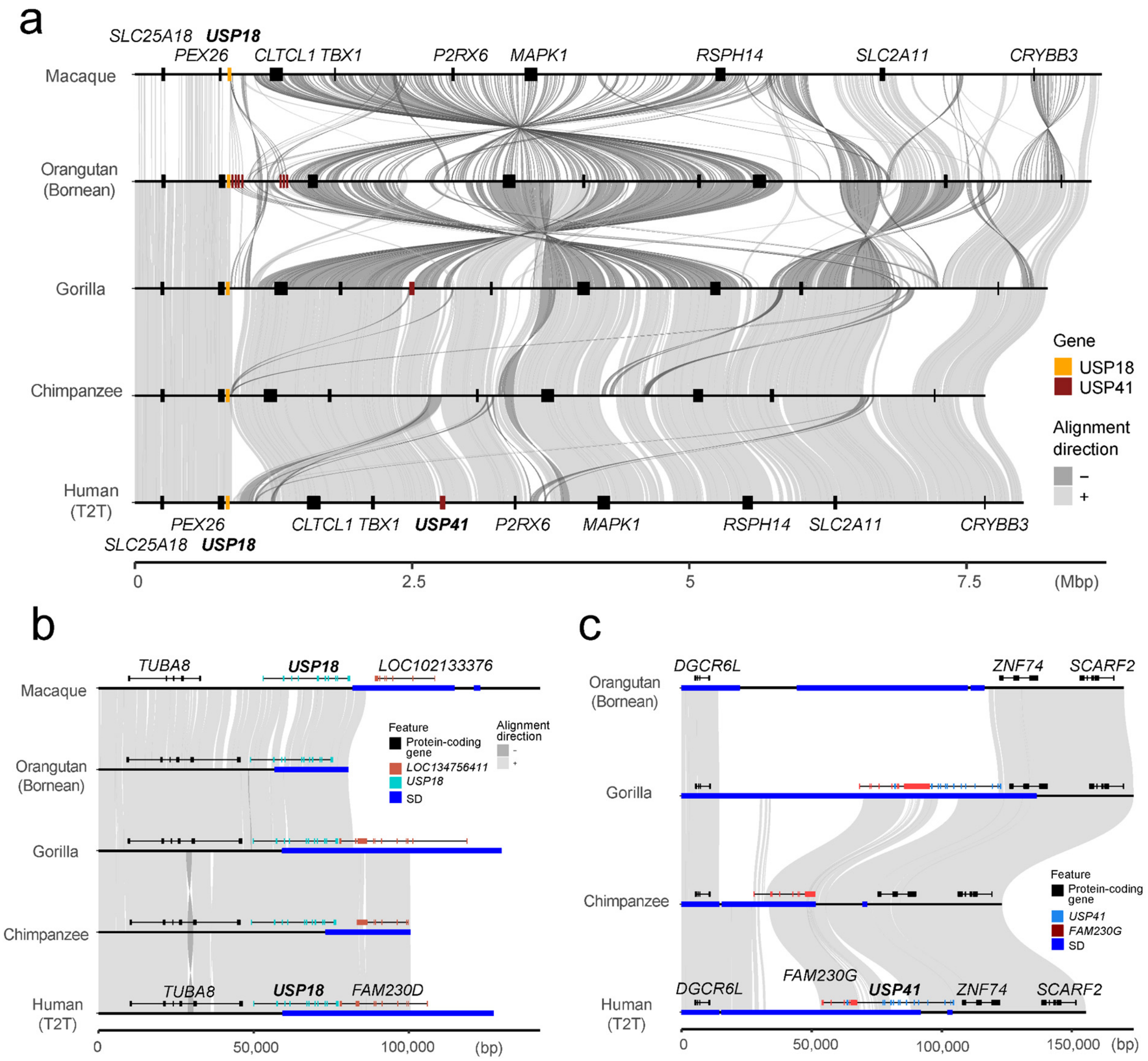

Supplementary Figure 55: Genomic position of *USP18* and *USP41*.
a) Position of all USP gene copies with respect to the 18-26 Mbp sequence of T2T-CHM13 reference genome. Zoomed-in view near **b**) *USP18* conserved across the primate lineage and **c**) *USP41* present only in human and gorilla genomes.

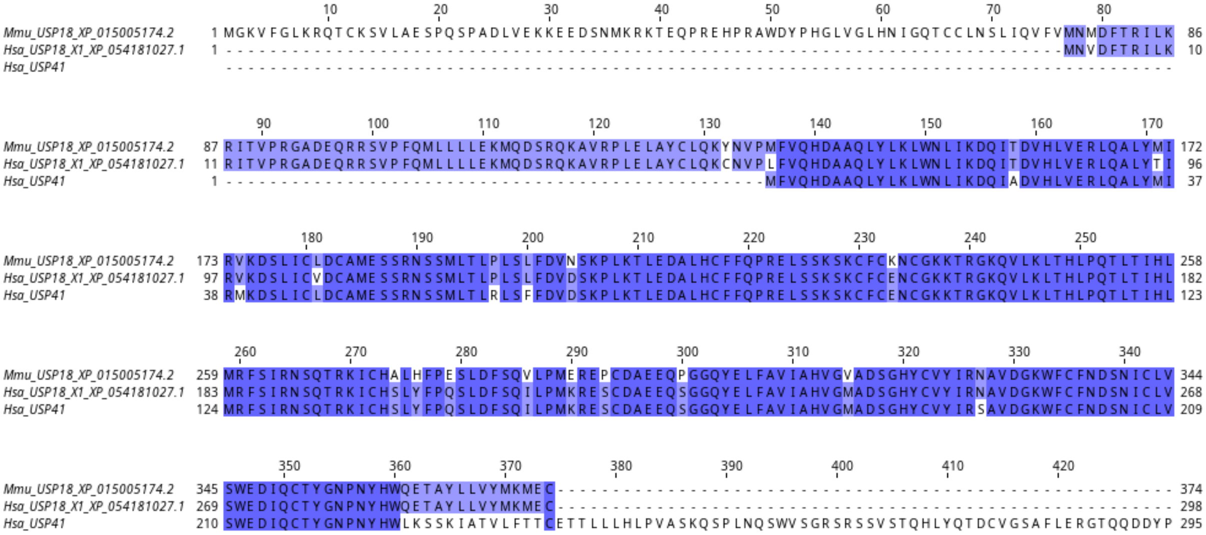

Supplementary Figure 56: MSA of USP41 protein sequence.
Reference annotations of human (HSA) and rhesus macaque (MMU) USP18, compared to human USP41 open reading frame predicted from Iso-Seq.

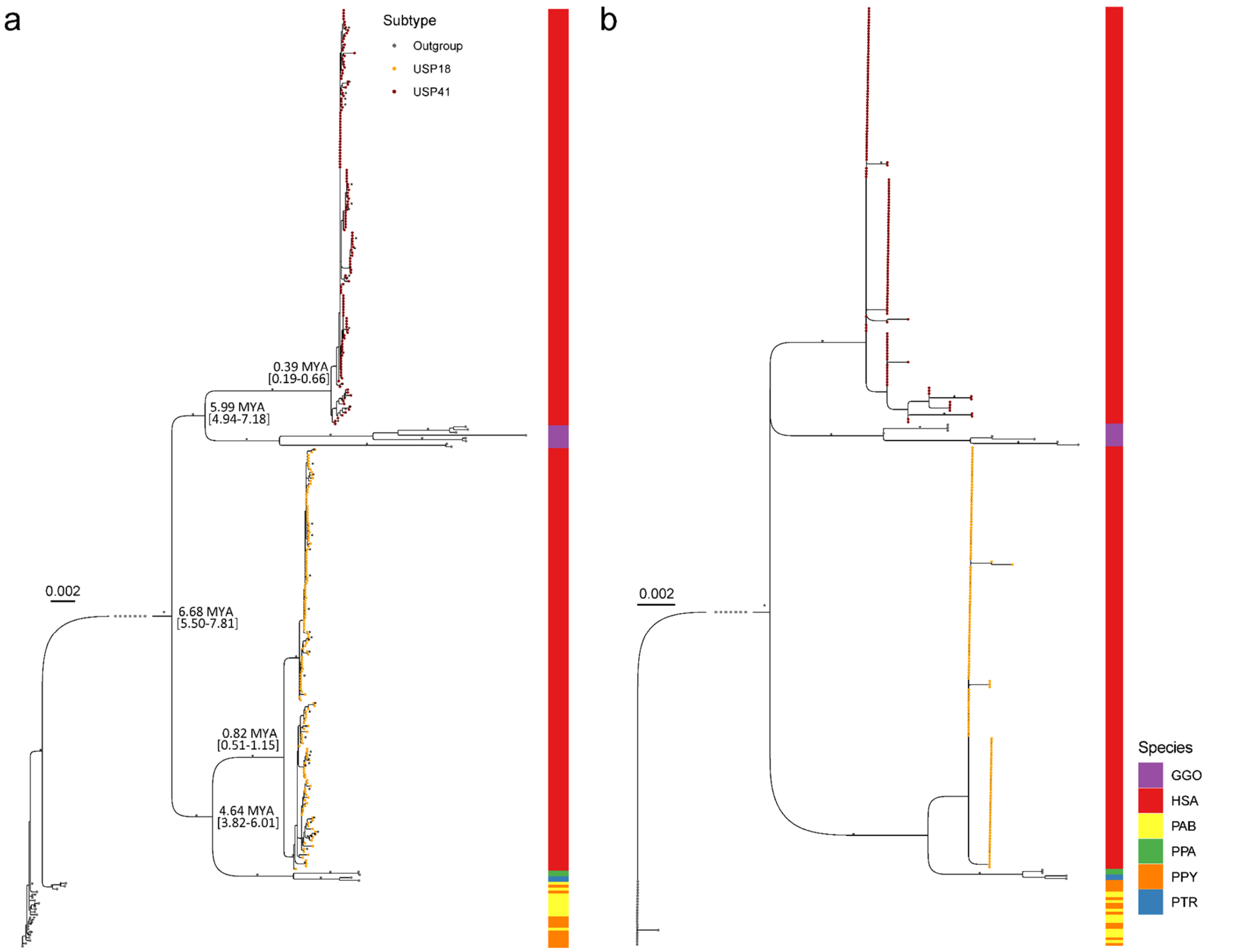

Supplementary Figure 57: Phylogeny of *USP18* and *USP41*.
Maximum likelihood tree of concatenated **a**) intron and **b**) exon sequences of USP gene copies. The tree was outgrouped with orangutan orthologs. The copies corresponding to *USP18* and *USP41* are indicated at the tip nodes of the trees in orange and red, respectively, while the nonhuman orthologs are indicated in gray. The corresponding species is indicated on the right annotation track: chimpanzee (PTR), bonobo (PPA), human (HSA), gorilla (GGO), Sumatran orangutan (PAB) and Bornean orangutan (PPY). Notable divergence times among the copies are indicated as million years ago (MYA) with the 95% confidence interval. The bootstrap value of >95 is indicated as an asterisk.

Models for dN/dS test

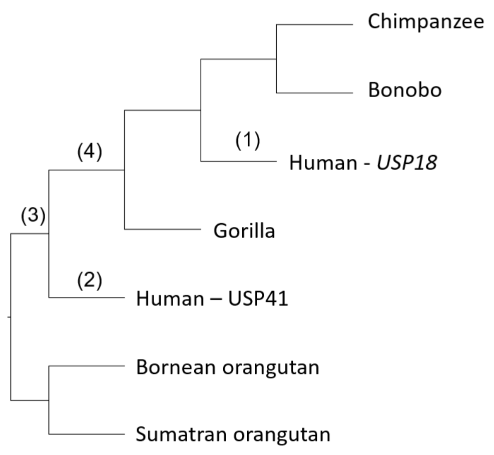

Model	(1)	(2)	(3)	(4)
Dnds	1.0	61.9	2.7	999.0
h0lnL	-950.3	-950.0	-949.6	-949.6
h1lnL	-950.3	-949.9	-949.4	-949.1
LRT	0.000	0.266	0.309	1.080
p-value	1.000	0.606	0.578	0.299
Putative sites under selection (BEB posterior)*	NA	27 P (0.646)<br>30 L (0.640)<br>157 N (0.638)	56 R (0.602)<br>70 H (0.604)<br>71 R (0.591)<br>80 F (0.605)<br>119 T (0.590)<br>121 Q (0.619)<br>130 P (0.609)<br>134 F (0.613)	NA

*site / amino acid / (probability)

Supplementary Figure 58: CODEML branch-site model test (dN/dS) of positive selection for the gene *USP41*. The modules (1-4) indicate the branch from which the dN/dS was estimated. On the right shows test statistics for individual models, including dN/dS (Dnds) ratio, log likelihood of null (neutral–fixing dN/dS to 1; h0lnL) and alternative (non-neutral–allowing dN/dS ratio to vary; h1lnL) hypothesis, log likelihood test statistics (LRT) and p-value derived from the LRT. Putative sites under selection reported are summarized at the bottom row, with none of the sites showing Bayes Empirical Bayes (BEB) posterior probability >0.95.

Supplementary References

1. Vollger, M. R. *et al.* Long-read sequence and assembly of segmental duplications. *Nat. Methods* **16**, 88–94 (2019).
2. Liao, W.-W. *et al.* A draft human pangenome reference. *Nature* **617**, 312–324 (2023).
3. Demaerel, W. *et al.* The 22q11 low copy repeats are characterized by unprecedented size and structural variability. *Genome Res.* **29**, 1389–1401 (2019).
4. Dierckxsens, N., Mansfield, M. J., Plessy, C., Luscombe, N. M. & Vermeesch, J. R. Unlocking the full potential of Oxford Nanopore reads with NOVOLoci. *bioRxiv* (2025) doi:10.1101/2025.08.08.669243.
5. Logsdon, G. A. *et al.* Complex genetic variation in nearly complete human genomes. *Nature* **644**, 430–441 (2025).
6. Dishuck, P. C. *et al.* Structural variation, selection, and diversification of the NPIP gene family from the human pangenome. *Cell Genom.* **5**, 100977 (2025).
7. Tong, M. *et al.* Polymorphisms of the 22q11.2 breakpoint region influence the frequency of de novo constitutional t(11;22)s in sperm. *Hum. Mol. Genet.* **19**, 2630–2637 (2010).
8. Vervoort, L. *et al.* Multiple paralogs and recombination mechanisms contribute to the high incidence of 22q11.2 deletion syndrome. *Genome Res.* **35**, 786–797 (2025).
9. Xie, F.-Y. *et al.* Downstream transcription promotes human recurrent CNV associated AT-rich sequence mediated genome rearrangements in yeast. *iScience* **27**, 111508 (2024).
10. Ohye, T. *et al.* Paternal origin of the de novo constitutional t(11;22)(q23;q11). *Eur. J. Hum. Genet.* **18**, 783–787 (2010).
11. Rubino, E. *et al.* Human ubiquitin-specific peptidase 18 is regulated by microRNAs via the 3'Untranslated region, A sequence duplicated in long intergenic non-coding RNA genes residing in chr22q11.21. *Front. Genet.* **11**, 627007 (2020).
12. Bonacci, T., Bolhuis, D. L., Brown, N. G. & Emanuele, M. J. Mechanisms of USP18 deISGylation revealed by comparative analysis with its human paralog USP41. *bioRxivorg* (2024) doi:10.1101/2024.05.28.596309.
13. Yoon, J.-Y., Seo, S.-U., Woo, S.-M. & Kwon, T.-K. USP41 enhances epithelial-mesenchymal transition of breast cancer cells through Snail stabilization. *Int. J. Mol. Sci.* **24**, 1693 (2023).
14. Kumar, S. *et al.* TimeTree 5: An expanded resource for species divergence times. *Mol. Biol. Evol.* **39**, (2022).
15. Pendleton, A. L. *et al.* Comparison of village dog and wolf genomes highlights the role of the neural crest in dog domestication. *BMC Biol.* **16**, 64 (2018).
16. Cooper, G. M. *et al.* A copy number variation morbidity map of developmental delay. *Nat. Genet.* **43**, 838–846

(2011).

17. Coe, B. P. *et al.* Refining analyses of copy number variation identifies specific genes associated with developmental delay. *Nat. Genet.* **46**, 1063–1071 (2014).

18. Jiang, Z., Hubley, R., Smit, A. & Eichler, E. E. DupMasker: a tool for annotating primate segmental duplications. *Genome Res.* **18**, 1362–1368 (2008).

19. Guo, X. *et al.* Variant discovery and breakpoint region prediction for studying the human 22q11.2 deletion using BAC clone and whole genome sequencing analysis. *Hum. Mol. Genet.* **25**, 3754–3767 (2016).
