# Supplementary material for: Population differences of chromosome 22q11.2 duplication structure predispose differentially to microdeletion and inversion
Source: Nat Commun. 2026 Apr 18;17:3701. doi: 10.1038/s41467-026-71905-y (PMC13103302; doi:10.1038/s41467-026-71905-y)
Supplement: Supplementary file 2 — Description of Additional Supplementary Files [file 41467_2026_71905_MOESM2_ESM.pdf]

Description of Additional supplementary files

Supplementary Data 1: Assembly validation using long-read datasets and HPRC2 (release 2) assemblies.

Supplementary Data 2: Assembly validation using published fiber-FISH and optical genome mapping data.

Supplementary Data 3: Large-scale inversions at chromosome 22q11.2.

Supplementary Data 4: Inverted duplications within LCRA and D at chromosome 22q11.2.

Supplementary Data 5: Definition of predisposed and protected samples based on interchromosomal interactions between LCRA and D at 22q11.2.

Supplementary Data 6: 22q11.2DS breakpoint locations within four families analyzed in this study.

Supplementary Data 7: Assembly statistics for samples sequenced and assembled in this study.

Supplementary Data 8: Long-read statistics for samples sequenced in this study.

Supplementary Data 9: Defined SD block boundaries in T2T-CHM13 used in this study.
