# Supplementary material for: Population differences of chromosome 22q11.2 duplication structure predispose differentially to microdeletion and inversion
Source: Nat Commun. 2026 Apr 18;17:3701. doi: 10.1038/s41467-026-71905-y (PMC13103302; doi:10.1038/s41467-026-71905-y)
Supplement: Supplementary file 12 — Reporting Summary [file 41467_2026_71905_MOESM12_ESM.pdf]

nature portfolio

Corresponding author(s): Evan E Eichler

Last updated by author(s): Mar 23, 2026

Reporting Summary

Nature Portfolio wishes to improve the reproducibility of the work that we publish. This form provides structure for consistency and transparency in reporting. For further information on Nature Portfolio policies, see our Editorial Policies and the Editorial Policy Checklist.

Statistics

For all statistical analyses, confirm that the following items are present in the figure legend, table legend, main text, or Methods section.

n/a	Confirmed	
☐	☒	The exact sample size (*n*) for each experimental group/condition, given as a discrete number and unit of measurement
☐	☒	A statement on whether measurements were taken from distinct samples or whether the same sample was measured repeatedly
☐	☒	The statistical test(s) used AND whether they are one- or two-sided<br>*Only common tests should be described solely by name; describe more complex techniques in the Methods section.*
☒	☐	A description of all covariates tested
☐	☒	A description of any assumptions or corrections, such as tests of normality and adjustment for multiple comparisons
☐	☒	A full description of the statistical parameters including central tendency (e.g. means) or other basic estimates (e.g. regression coefficient) AND variation (e.g. standard deviation) or associated estimates of uncertainty (e.g. confidence intervals)
☐	☒	For null hypothesis testing, the test statistic (e.g. *F*, *t*, *r*) with confidence intervals, effect sizes, degrees of freedom and *P* value noted<br>*Give P values as exact values whenever suitable.*
☒	☐	For Bayesian analysis, information on the choice of priors and Markov chain Monte Carlo settings
☒	☐	For hierarchical and complex designs, identification of the appropriate level for tests and full reporting of outcomes
☒	☐	Estimates of effect sizes (e.g. Cohen's *d*, Pearson's *r*), indicating how they were calculated

Our web collection on statistics for biologists contains articles on many of the points above.

Software and code

Policy information about availability of computer code

Data collection	The software used to collect sequencing data are PacBio SMRT Link (v11.0.1, 12.0, 13.0, and 25.1) and Oxford Nanopore Technologies MinKNOW software (v24.06.15 - 25.03.7).
Data analysis	Publicly available software used in this study include:<br>hifiasm (v0.16.1, v0.19.5, v0.24.0, or v0.25.0), NOVOLoci (v0.5), minimap2 (v2.24, v2.26, and v2.28), rustybam (v0.1.33, 10.5281/zenodo.8106233), DupMasker (v4.1.2-p1), MAFFT (v7.525), Gblocks (v0.91b), IQtree (v2.3.6), Liftoff (v1.6.3), SQANTI3 (v5.2), PAV (v2.3.4).<br>We also used following R packages:<br>SVbyEye (v0.99), ClusterR (v1.3.2), DECIPHER (v3.2.0), GenomicRanges (v1.54.1), stringdist (v0.9.12), phangorn (v2.11.1), rtracklayer (v1.62.0).

For manuscripts utilizing custom algorithms or software that are central to the research but not yet described in published literature, software must be made available to editors and reviewers. We strongly encourage code deposition in a community repository (e.g. GitHub). See the Nature Portfolio guidelines for submitting code & software for further information.

Data

Policy information about availability of data

All manuscripts must include a data availability statement. This statement should provide the following information, where applicable:

- Accession codes, unique identifiers, or web links for publicly available datasets
- A description of any restrictions on data availability
- For clinical datasets or third party data, please ensure that the statement adheres to our policy

All datasets generated in this study are available via European Nucleotide Archive (ENA) (n=15) or Zenodo (n=279). Samples with restricted access (n=9) are available via European Genome-phenome Archive (EGA).

Research involving human participants, their data, or biological material

Policy information about studies with human participants or human data. See also policy information about sex, gender (identity/presentation), and sexual orientation and race, ethnicity and racism.

Reporting on sex and gender	We only report sex and gender with respect to members of the 22q11.2DS patients. We do not comment on any sex and gender biases in our results as those were not relevant to our conclusions.
Reporting on race, ethnicity, or other socially relevant groupings	We report analyses of publicly available human genome sequencing data generated by the 1000 Genomes Project (https://www.internationalgenome.org/home) and their associated genetic ancestry information, as established and described by the 1000 Genomes Project (https://www.internationalgenome.org/category/population/).
Population characteristics	see above
Recruitment	see above
Ethics oversight	Study approval for three family duos (AD009, AD010, and AD013) was obtained from the Medical Ethics Committee of the University Hospital/KU Leuven (S62997) along with approval for data sharing with the University of Washington (S67964).

Note that full information on the approval of the study protocol must also be provided in the manuscript.

Field-specific reporting

Please select the one below that is the best fit for your research. If you are not sure, read the appropriate sections before making your selection.

☒ Life sciences ☐ Behavioural & social sciences ☐ Ecological, evolutionary & environmental sciences

For a reference copy of the document with all sections, see nature.com/documents/nr-reporting-summary-flat.pdf

Life sciences study design

All studies must disclose on these points even when the disclosure is negative.

Sample size	We analyzed whole-genome assemblies from diverse humans generated by the Human Pangenome Reference Consortium (HPRC, release1 and release 2; n=47 and n=232, respectively) and Human Genome Structural Variation Consortium (HGSVC; n=65). We analyzed long-read datasets for an additional two human samples (HG01888 and NA19315) along with short-read Strand-seq datasets (n=292). Lastly, we have processed long-read datasets of clinical samples (n=9).
Data exclusions	We excluded redundant samples assembled in both HPRC and HGSVC datasets and kept only unique sample assemblies.
Replication	N/A
Randomization	N/A
Blinding	N/A

Reporting for specific materials, systems and methods

We require information from authors about some types of materials, experimental systems and methods used in many studies. Here, indicate whether each material, system or method listed is relevant to your study. If you are not sure if a list item applies to your research, read the appropriate section before selecting a response.

Materials & experimental systems

n/a	Involved in the study
☒	☐ Antibodies
☐	☒ Eukaryotic cell lines
☒	☐ Palaeontology and archaeology
☒	☐ Animals and other organisms
☐	☒ Clinical data
☒	☐ Dual use research of concern
☒	☐ Plants

Methods

n/a	Involved in the study
☒	☐ ChIP-seq
☒	☐ Flow cytometry
☒	☐ MRI-based neuroimaging

Eukaryotic cell lines

Policy information about cell lines and Sex and Gender in Research

Cell line source(s)	NHGRI Sample Repository for Human Genetic Research at the Coriell Institute for Medical Research and NIGMS Human Genetic Cell Repository
Authentication	N/A
Mycoplasma contamination	There was no suspicion of mycoplasma contamination so no test was done.
Commonly misidentified lines (See ICLAC register)	N/A

Clinical data

Policy information about clinical studies

All manuscripts should comply with the ICMJE guidelines for publication of clinical research and a completed CONSORT checklist must be included with all submissions.

Clinical trial registration	N/A
Study protocol	N/A
Data collection	N/A
Outcomes	N/A

Plants

Seed stocks	N/A
Novel plant genotypes	N/A
Authentication	N/A
